# Inducing Reactivity by Cluster Strain in Titanium Frameworks

**DOI:** 10.1021/jacs.5c16069

**Published:** 2025-12-24

**Authors:** Eloy P. Gómez-Oliveira, Vitor Fernandes de Almeida, Javier Castells-Gil, Herme G. Baldoví, Felipe Gándara, Neyvis Almora-Barrios, Sergio Tatay, Sergio Navalón, Natalia M. Padial, Carlos Martí-Gastaldo

**Affiliations:** † Functional Inorganic Materials Team, Instituto de Ciencia Molecular (ICMol), 253325 Universitat de València, 46980 València, Spain; ‡ Department of Chemistry, 16774 Universitat Politècnica de València, Camino de Vera, s/n, 46022 València, Spain; § School of Chemistry, 67089 University of Birmingham, Birmingham, B152TT, United Kingdom; ∥ Instituto de Ciencia de Materiales de Madrid (ICMM-CSIC), Sor Juana Inés de la Cruz 3, 28049 Madrid, Spain; ⊥ Current affilation: Departamento de Química Física, Universitat de València, Carrer del Doctor Moliner, 50, 46100 Burjassot, Spain

## Abstract

Despite their potential to control charge separation and redox activity, deliberate strategies to distort metal–oxo clusters in molecular frameworks remain limited. Here we present a proof-of-concept for cluster strain engineering using the titanium–organic framework MUV-10 as a model. Replacing Ca^2+^ with larger alkaline-earth cations (Sr^2+^, Ba^2+^) induces predictable distortions of Ti_2_M_2_ clusters and a cubic-to-tetragonal cell transformation while preserving the overall connectivity. This local strain alters Ti–O coordination geometry, enhances ligand-to-metal charge transfer, and promotes the photogeneration of Ti^3+^ sites, as validated by photocatalytic CO_2_ methanation under standardized conditions. Importantly, the extent of distortion follows the trend anticipated from the Goldschmidt tolerance factor, a classical descriptor from perovskite chemistry, that we repurpose here to rationalize strain in reticular frameworks. Taken together, these findings establish a conceptual link between oxide catalysis and reticular chemistry, highlighting cluster strain as a potential structural switch to modulate redox reactivity in molecular solids.

## Graphical abstract

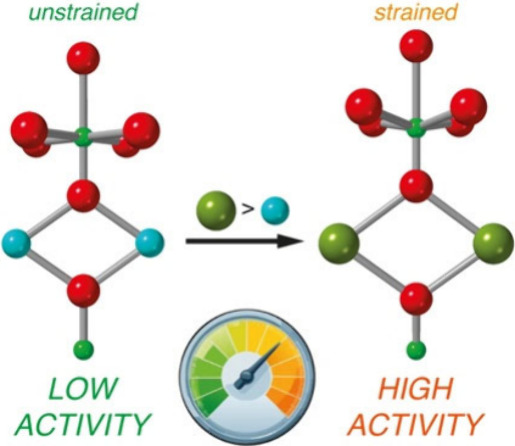

## Introduction

Among the many applications of metal–organic frameworks (MOFs), heterogeneous catalysis stands out as a particularly compelling example of their potential. Their modular architecture, structural diversity, and compositional flexibility allow for the precise positioning of catalytic centers, fine-tuning of electronic environments, and tailoring of channel size, shape, and chemistry all useful to delineate structure–composition–activity correlations that support the rational design of advanced molecular catalysts.^1^ Titanium-based MOFs have emerged as a central platform in molecular photocatalysis, owing to the natural abundance of this metal, its favorable chemical stability against hydrolysis associated with the strong polarization of Ti^4+^ ions, and its ability to generate reactive Ti^3+^ species upon photoexcitation or electroreduction.

Despite their distinct electronic structure compared to classical oxide semiconductors, the development of Ti-based MOFs has been driven by the ambition to create porous and crystalline analogues of TiO_2_.^2,3^ This comparison has shaped the design of these materials, even though their photocatalytic behavior is often limited to localized ligand-to-metal charge transfer (LMCT) processes due to their molecular nature.^4^ Since the initial report of MIL-125,^5^ significant progress has been made in addressing the challenges posed by the solution chemistry of titanium for a more systematic understanding of its ability to form extended frameworks as exemplified by MIL-177/100/101,^6–8^ COK-69/47,^9,10^ PCN-415/416,^11^ MOF-901/217,^12,13^ DGIST-1,^14^ MUV-10/11/12/35/101/301,^15–20^ MIP-207/208/209,^21–23^ ACM-1/4,^24,25^ NU-1012 ^26^ or SNNU-90/92.^27^ The modular nature, structural diversity, and compositional versatility of these porous molecular architectures have been central to optimizing their photocatalytic performance whether through visible-light sensitization, enhanced charge transfer, controlled substrate interaction, or fine-tuning of their electronic energy levels to match the redox potentials required for processes such as organic pollutant degradation, water splitting, CO_2_ reduction, or photoredox transformations.^28–30^ Although numerous strategies are used to tailor their photocatalytic activity, including linker functionalization, metal substitution, the incorporation of visible-light sensitizers or cocatalysts, and the construction of heterojunctions or hybrid systems by combining MOFs with other functional materials, deliberate use of local distortion around titanium centers has not yet been considered. This contrasts with classical oxide photocatalysts such as TiO_2_, BiVO_4_, or SrTiO_3_, where distortions introduced by doping with larger cations are known to generate active sites, induce local electric fields, and enable symmetry-breaking electronic transitions that can enhance light absorption, facilitate energy transfer, and improve charge separation.^31,32^

Here, we introduce local strain engineering in titanium frameworks as a conceptual approach to enhancing photocatalytic activity (Figure 1). By progressively increasing the ionic radius of nontitanium metal centers in the MUV-10 family, we induce a controlled distortion of Ti–O coordination sites, resulting in a cubic-to-tetragonal transformation of the framework. We show that this controlled structural distortion enhances LMCT, promotes the generation of Ti^3+^ active sites, and enhances photocatalytic CO_2_ methanation. These findings establish cluster strain as a previously unexplored structural variable in reticular chemistry with direct consequences for the activation of redox active titanium centers.

**Figure 1:**
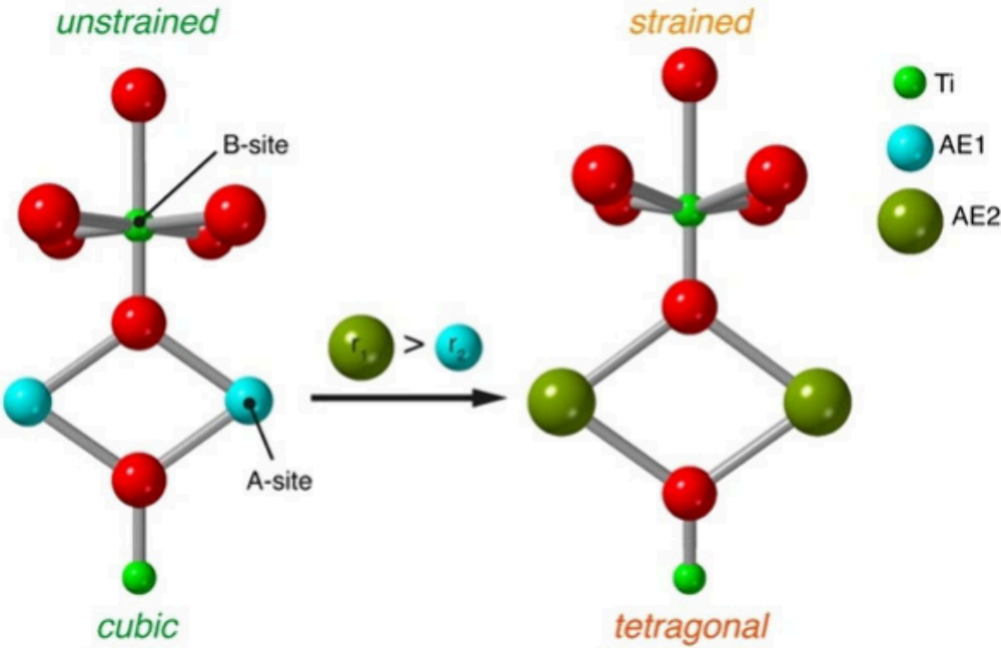
Schematic representation of the local strain induced in the Ti_2_M_2_ cluster core of MUV-10 upon substitution of A-site alkaline-earth (AE) cations with larger ionic radii. The substitution distorts the original geometry around the Ti octahedra (B-sites). The diagram highlights the causal relationship between the ionic radius increase (r_1_ > r_2_) and structural symmetry breaking of the cell from cubic to tetragonal.

## Results and Discussion

### Structural Tuning of MUV-10 Frameworks via A-Site Cation Substitution

The structure of MUV-10 is based on bimetallic Ti_2_M_2_ tetramers in which Ti^4+^ octahedra (B-sites) are interconnected via *μ*_*2*_-carboxylate and *μ*_*3*_-oxo bridges to Ca^2+^ ions (A-sites) arranged in a capped trigonal prismatic coordination environment (Figure 1).^15^ According to our previous work, substitution with other first-row divalent transition metals is compatible only with high-spin *d* ^5^ Mn^2+^ which preserves the original cluster geometry. In this context, we have shown that postsynthetic metal exchange of Ca^2+^ in MUV-10 crystals with other 3d metals triggers a transformation of the cluster core, leading to the formation of zeotype structures such as MUV-101 ^33^ and MUV-301 ^19^ built from TiM_2_ oxo trimers.

Here, we hypothesized that homovalent substitution with larger alkaline-earth metals could preserve the coordination motif while introducing a size-induced distortion. Analogous to the mismatch between A- and B-site cations in distorted perovskites, this substitution was expected to generate local strain within the metal–oxo cluster, altering metal–oxygen bond angles and distances and modifying the electronic structure with direct consequences for photocatalytic activity. To test this hypothesis, the obvious choice was to replace A-site Ca^2+^ (1.06 Å) with Sr^2+^ (1.18 Å) and Ba^2+^ (1.47 Å).^34^ Ra^2+^ (1.48 Å) was excluded due to its radioactivity.

The synthetic conditions originally reported by our group for the preparation of MUV-10­(Ca/Mn)^15^ led to poorly crystalline Sr and Ba analogues, with insufficient order to allow structural refinement and analysis of cluster distortion. This observation is consistent with previous reports by Brozek and co-workers, who also encountered limited crystallinity in isostructural MUV-10­(M) (M = Ca, Sr, Ba) frameworks under similar conditions.^35^ These findings suggest a greater difficulty in incorporating the larger alkaline-earth metals into the framework, likely due to their increased ionic radii. To circumvent this limitation, we increased the concentration of the modulator and used a preformed titanium cluster as a metal source to stabilize Ti^4+^ in solution and suppress the formation of amorphous phases. Specifically, doubling the amount of acetic acid and replacing titanium isopropoxide with [Ti_6_O_6_(O^*i*^Pr)_6_(4-tbbz)_6_] (tbbz = 4-*tert*-butylbenzoic acid)^7^ enabled the synthesis of highly crystalline MUV-10­(Sr) and MUV-10­(Ba) (Figure 2). These optimized conditions were also compatible with the preparation of the Ca-based analogue. Further details are provided in Supplementary Section S2.1.

**Figure 2:**
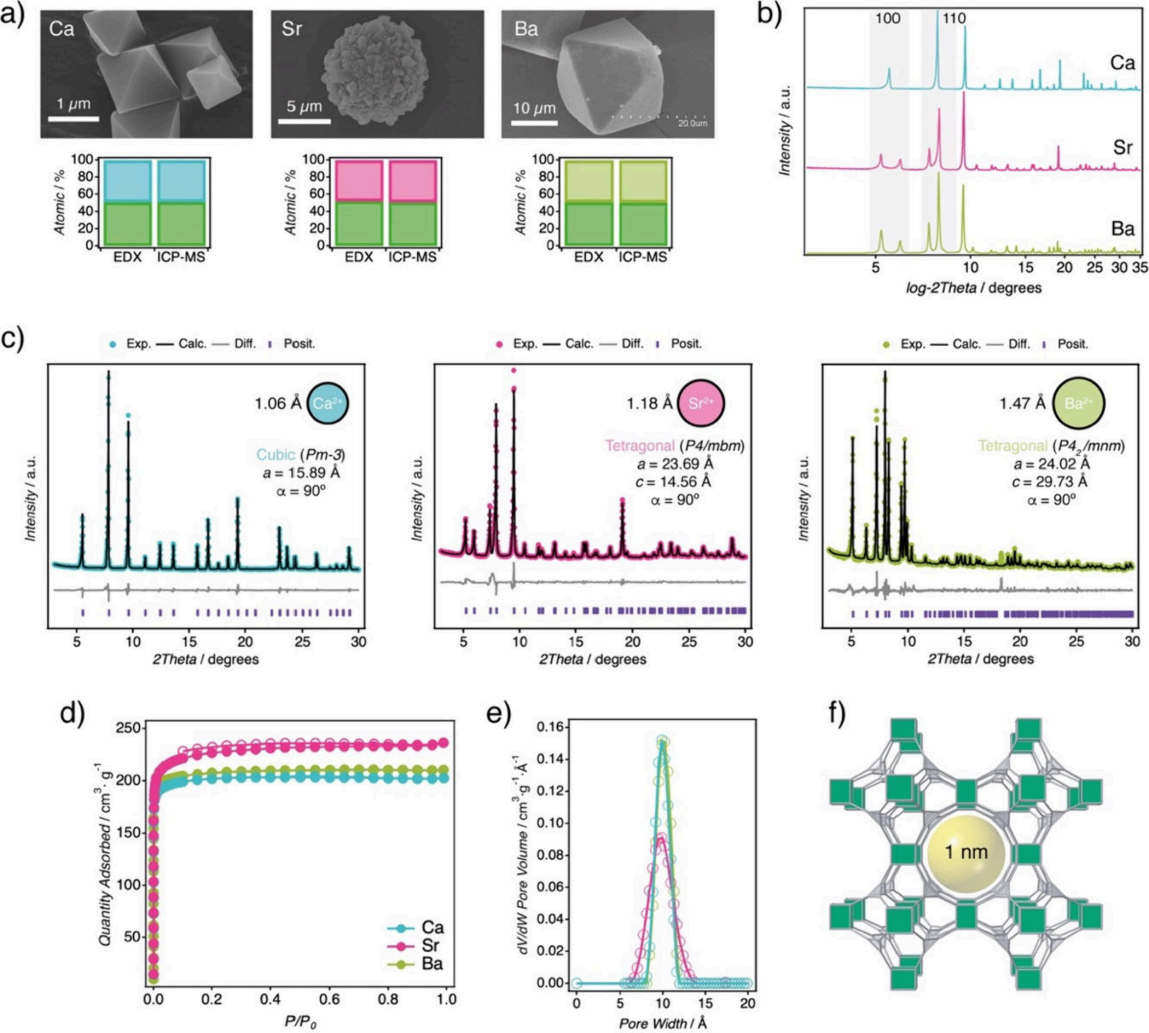
A-site substitution in MUV-10 frameworks. a) SEM images and elemental analysis (EDX and ICP-MS) of MUV-10­(Ca), MUV-10­(Sr), and MUV-10­(Ba). MUV-10­(Sr) forms spherical aggregates composed of intergrown nanocrystals with visible twinning, in contrast to the faceted morphologies observed for the Ca and Ba analogues. b) PXRD patterns showing the splitting of the 100 and 110 reflections in Sr and Ba phases, indicative of symmetry lowering relative to the parent cubic structure. c) Le Bail refinements and unit cell parameters confirming the cubic-to-tetragonal distortion in the Sr and Ba phases (see Supplementary Section S.2.7 for details). d) N_2_ adsorption (filled symbols) and desorption (open symbols) isotherms at 77 K confirm the microporous character of all materials. e) Experimental pore size distributions centered at ∼1 nm. f) Structural model highlighting the channel apertures present in MUV-10.

Although all materials could be isolated as crystalline solids in Figure 2a, well-defined micrometric octahedral crystals were only observed for MUV-10­(Ca) and MUV-10­(Ba). In contrast, the Sr derivative crystallized as spherical microspheres composed of aggregated, intergrown nanocrystals, displaying clear signs of twinning. Scanning electron microscopy–energy dispersive X-ray spectroscopy (SEM–EDX) analysis combined with inductively coupled plasma mass spectrometry (ICP–MS) of acid-digested samples confirmed the presence of Ti and the corresponding A-site metals in stoichiometries consistent with the expected Ti_2_M_2_ cluster. X-ray photoelectron spectroscopy (XPS) revealed the presence of Ti^4+^ species accompanied by Ca^2+^, Sr^2+^ or Ba^2+^ ones and expected signals from trimesate organic ligand (Supplementary Section S2.5). Powder X-ray diffraction (PXRD) patterns reveal a splitting of 100 and 110 reflections characteristic of MUV-10­(Ca) in the Ba and Sr analogues (Figure 2b), indicating a deviation from cubic symmetry. This splitting provides the first evidence of a symmetry-lowering structural response associated with the increasing ionic radius of the nontitanium metals. Le Bail refinement of high-resolution PXRD patterns confirmed this distortion (Figure 2c). While MUV-10­(Ca) adopts a cubic structure (space group *Pm*3̅) with a cell parameter of *a* = 15.89 Å, substitution with Sr^2+^ and Ba^2+^ induces a cubic-to-tetragonal distortion. The resulting materials crystallize in the tetragonal space groups *P*4/*mbm* and *P4*_*2*_/*mnm*, with unit cell parameters of *a* = 23.84 and 24.02 Å and *c* = 14.76 and 29.73 Å, respectively. This structural expansion reflects the accommodation of larger A-site cations within the framework. Crystals suitable for single-crystal X-ray diffraction (SCXRD) analysis were obtained only in the case of MUV-10­(Ba). The structural solution and refinement of an as-synthesized crystal (Table S4) confirmed the unit cell dimensions previously derived from PXRD data, notably showing a doubling of the *c* parameter compared to MUV-10­(Sr). Careful inspection of the structure indicates that this change in cell volume and symmetry is associated with the presence of solvent molecules in the vicinity of the inorganic secondary building units, which influence the local geometry and packing. N_2_ adsorption–desorption isotherms confirm that all materials retain the microporous nature of MUV-10, showing type-I, nonhysteretic profiles and Brunauer–Emmett–Teller (BET) surface areas in the range of 900–1.000 m^2^·g^–1^ (Figure 2d). The corresponding pore size distribution (PSD) curves are consistent with ∼1 nm cavities typical of this framework. The Sr analogue displays a broader PSD, likely due to its polycrystalline morphology and the contribution of interparticle voids between aggregated nanocrystals (Figure 2e).

### Geometric Distortion and Cluster Strain across the MUV-10­(M) Series

As shown in Figure 3a, the structures of MUV-10­(Sr) and MUV-10­(Ba), determined by Rietveld refinement and single-crystal analysis, respectively (Supplementary Section S3), confirm the formation of isoreticular frameworks exhibiting the cubic-to-tetragonal distortion anticipated from PXRD data.

**Figure 3:**
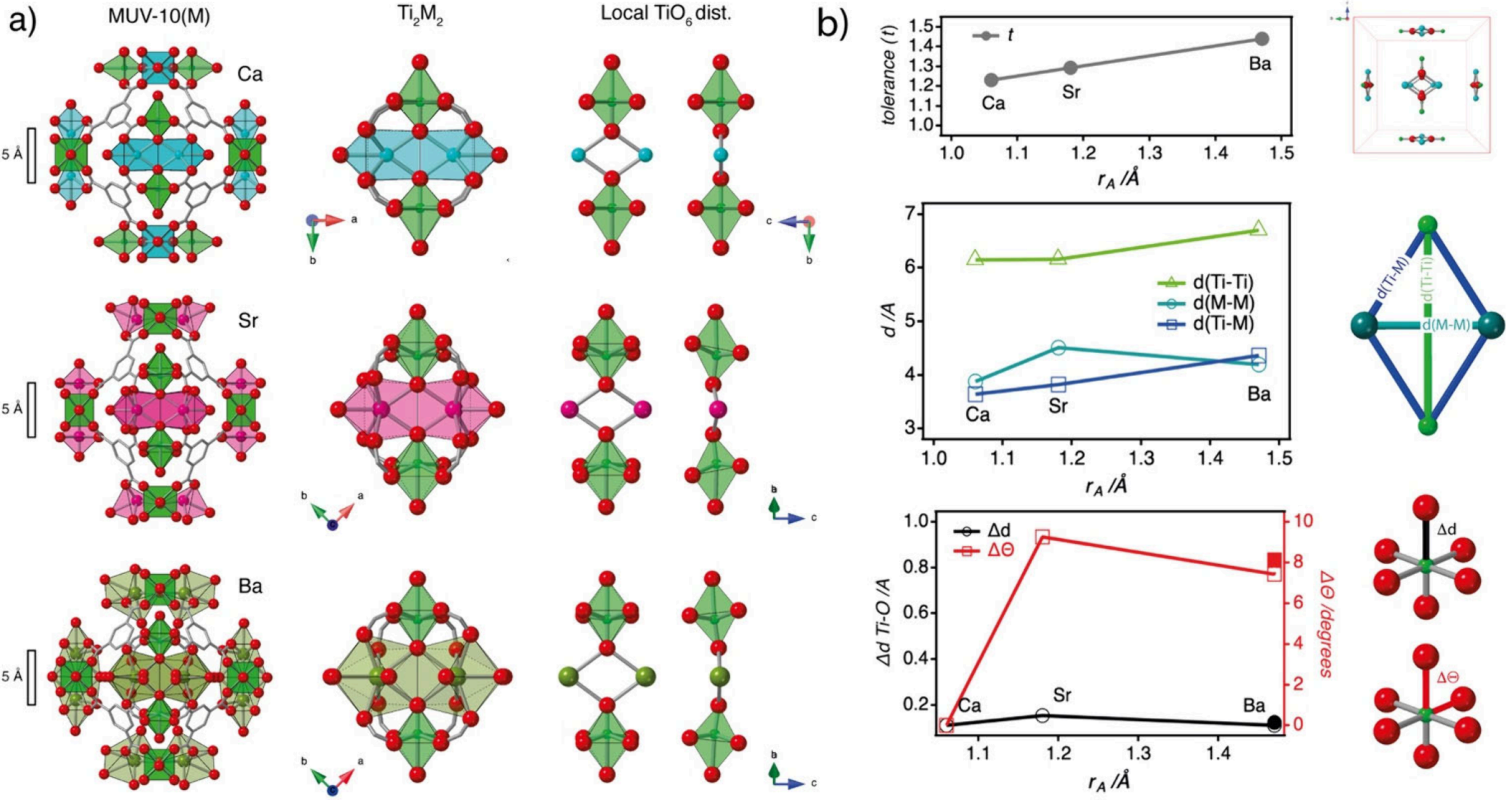
Quantifying local distortion and symmetry breaking. a) Structural comparison of MUV-10­(Ca), MUV-10­(Sr), and MUV-10­(Ba), showing the extended framework (left), the central Ti_2_M_2_ cluster (middle), and the local TiO_6_ environment (right). The increasing ionic radius of the A-site cation induces a progressive distortion of the regular TiO_6_ octahedra at the B-site, which results in a cubic-to-tetragonal transformation of the crystallographic unit cell. b) Quantitative analysis of structural parameters as a function of A-site ionic radius (r_A_): *Top*: Simplified tolerance factor (*t*) increases with the A-site radius. *Middle*: Interatomic distances within the Ti_2_M_2_ unit (Ti–Ti, M–M, and Ti–M). *Bottom*: Octahedral distortion metrics: Δd (Ti–O bond length deviation) and Δθ (angular deviation from 90° O–Ti–O angles) are represented by empty symbols. Filled symbols correspond to MUV-10­(Ba) after thermal evacuation at 100 °C, showing that structural distortion is controlled by the A-site cation. The schematics at right illustrate how distortion is measured based on bond geometry and intermetallic metrics. MUV-10­(Ca) parameters are derived from CCDC1819267; structures for Sr and Ba are provided in this work.

To rationalize this symmetry lowering, we adapted the geometric concept of Goldschmidt’s tolerance factor, commonly used to rationalize structural distortions in perovskite oxides (Supplementary Section S3.3).^36^ In our framework, the simplified tolerance factor (*t*) is defined as *t* = (*r*_*A*_ + *r*_*X*_)/[2·(*r*_*B*_ + *r*_*X*_)]^1/2^ where r_A_, r_B_ and r_X_ are the ionic radii of the A-site cation, B-site cation (Ti^4+^) and bridging anion (O^2–^) (Figure 3b, top). The calculated *t* values increase from 1.23 (Ca) to 1.29 (Sr) and 1.44 (Ba), correlating with a progressive deviation from cubic symmetry driven by the larger ionic radii of the alkaline-earth cations.

Despite this distortion, the local Ti coordination remains octahedral across the series. However, the A-site metals exhibit increasing coordination numbers (6–9), often involving disordered water molecules. As shown in Figure 3b (middle), the Ti–M distance increases steadily from 3.64 Å (Ca) to 4.37 Å (Ba), while Ti–Ti separations remain nearly constant for Ca and Sr (∼6.15 Å) and expand only in Ba (6.70 Å). M–M distances show a trend with ionic radius that follows the changes in Ti–Ti and Ti–M distances, with Sr exhibiting the largest separation (4.51 Å). To quantify local distortion at the Ti site, we calculated the bond length deviation (Δd) and angular distortion (Δθ) from ideal TiO_6_ geometry (Figure 3b, bottom). While Δd remains almost unchanged across the series, Δθ increases sharply for Sr and Ba, exceeding 7.4°, indicating significant octahedral tilting induced by A-site expansion. This angular distortion stems from the out-of-plane displacement of carboxylate oxygen atoms and reflects the buildup of local strain within the Ti_2_M_2_ cluster.

At this point, it is important to consider whether the observed distortion could arise not only from the intrinsic nature of the A-site cation but also from differences in the solvation state of the framework. To address this question, we performed an additional structural analysis of MUV-10­(Ba) after thermal evacuation at 100 °C. Remarkably, the crystal structure of the activated sample also adopts the tetragonal *P*4/*mbm* space group, with unit cell parameters *a* = 23.42 Å and *c* = 14.47 Å (Supplementary Section S3.2, Table S4), corresponding to a unit cell volume nearly half that of the as-synthesized material. This reduced cell is comparable to that of MUV-10­(Sr), indicating that the doubled *c* parameter observed in the solvated Ba analogue originates from packing effects related to solvent inclusion. Moreover, the refined structure exhibited only minor changes in the distortion metrics relative to the as-synthesized material, with Δd = 0.125 Å and Δθ = 8.16° (filled symbols in Figure 3b, bottom). These results confirm that the characteristic octahedral distortions and symmetry lowering persist upon solvent removal, supporting the idea that the ionic radius of the A-site cation is the primary factor governing cluster strain in the MUV-10 series.

### Strain-Driven Modulation of Ti^3+^ Photogeneration

Having established that the incorporation of larger alkaline-earth metals induces local distortion in the MUV-10­(M) series, we next examined how these structural changes affect CO_2_ adsorption, optical properties, and electronic structure, all factors relevant to the photocatalytic hydrogenation of CO_2_.^30^

The CO_2_ adsorption isotherms collected at 298 K display a gradual uptake with pressure (Figure 4a). The absence of a sharp low-pressure adsorption indicates relatively weak guest–host interactions, with no evidence of chemisorption or strong electrostatic interactions. Nonetheless, all materials exhibit competitive uptake values at room temperature, with Sr and Ba surpassing 3.5 mmol·g^–1^ at 1 bar, comparable to benchmark adsorbents as HKUST-1­(Cu) and MIL-101­(Cr).^37^ Compared to the undistorted Ca analogue, the distorted Sr and Ba phases show slightly higher isosteric heats of adsorption at low coverage (*Q*_*st*_ > 30 kJ·mol^–1^), suggesting that symmetry breaking at Ti­(IV) sites enhances the polarizability of the adsorption sites, thereby strengthening the CO_2_-framework interactions.

**Figure 4:**
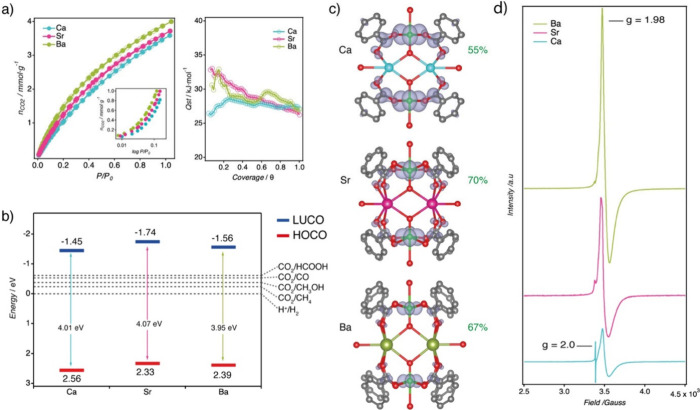
CO_2_uptake, orbital contributions and Ti^3+^photogeneration in MUV-10­(M). a) CO_2_ adsorption isotherms for the MUV-10­(M) series at 298 K. The inset highlights the low-pressure region in logarithmic scale. The isosteric heat of adsorption (Q_st_) was derived from isotherms collected at multiple temperatures. b) Experimental band edge positions (HOCO and LUCO) and optical band gaps (Eg), aligned to the NHE scale. c) Electron density distribution of the LUCO, highlighting the increased Ti orbital character upon A-site substitution. d) EPR spectra after UVA irradiation confirming Ti^3+^ formation and modulation of LMCT efficiency with cluster strain.

Optical band gaps (*E*_*g*_) were estimated from UV–vis diffuse reflectance data using the Kubelka–Munk function (Supplementary Section S4.2.). Only minor variations were observed across the series, with values near 4.0 eV, confirming that light absorption remains restricted to the UV region and is predominantly governed by the organic linker. To probe the frontier orbital energetics, we experimentally determined the positions of the highest occupied crystal orbital (HOCO) and lowest unoccupied crystal orbital (LUCO) using ultraviolet photoelectron spectroscopy (UPS, Figure 4b). The lowest unoccupied crystal orbital (LUCO) energy edge was determined by subtracting the HOCO value from the optical band gap. Importantly, metal substitution and the associated distortion in the Sr and Ba analogues induce a systematic shift of both HOCO and LUCO toward more negative potentials. Although the LUCO levels in all MOFs lie well above the thermodynamic potential for CO_2_-to-CH_4_ reduction (−0.24 V vs. NHE), MUV-10­(Sr) with −1.74 eV shows the largest thermodynamic driving force of the series for photoactivated multielectron CO_2_ reduction.

While band edge diagrams provide a thermodynamic view of the MUV-10­(M) systems, they do not capture the kinetic complexity of LMCT processes required to generate catalytically active Ti^3+^ centers. These photoreduced sites often represent the efficiency-limiting step in photocatalytic MOFs.^4,38^ To better understand these processes, we calculated the electronic structures using density functional theory (DFT), based on the experimental geometries of the MUV-10­(M) series (see Supplementary Section S7 for details). The projected density of states (DOS) reveals clear differences in the orbital contributions to the LUCO across the series. As shown in Figure 4c, the structural distortion induced by A-site substitution modifies the local electronic environment of the Ti centers, shifting the nature of the LUCO from a relatively balanced ligand/metal contribution in MUV-10­(Ca) of (45:55%) to a predominantly metal-centered orbital in MUV-10­(Sr) (70%) and MUV-10­(Ba) (67%). This trend suggests that local symmetry breaking enhances LMCT character, potentially facilitating the more efficient photoreduction of Ti^4+^ to catalytically active Ti^3+^ species. This effect is only captured when using the experimentally determined, distorted structures as input for DFT calculations. In contrast, previous computational studies based on undistorted, cubic MUV-10­(Ca)-like structures for the same derivatives predicted only minimal changes in electronic structure upon substitution.^35^

To validate this prediction, we recorded electron paramagnetic resonance (EPR) spectra of MUV-10­(M) samples dispersed in deoxygenated acetonitrile and sealed in quartz tubes, after irradiation with a Kessil PR-160L lamp (370 nm, 100% LED intensity) for 12 h at room temperature (Figure 4d). In all cases, the spectra reveal the formation of Ti^3+^ species, evidenced by an asymmetric singlet at 0.35 T (g ≈ 1.98), consistent with photoinduced one-electron reduction of Ti^4+^. A second resonance at g = 2.00, attributed to a ligand-centered radical, is also observed. Notably, this radical signal is most intense for MUV-10­(Ca) and progressively diminishes for the Sr and Ba analogues despite comparable measurement conditions and sample quantities (Supplementary Section S.8). This trend suggests that as structural strain increases, LMCT becomes more efficient, favoring the generation of Ti^3+^ species at the expense of the ligand radical. In parallel, the narrowing of the Ti^3+^ signal for a more isotropic shape with increasing strain may indicate the suppression of spin–spin coupling between the Ti center (S = 1/2) and the ligand radical (S = 1/2), consistent with a more complete and spatially decoupled electron transfer process in the distorted frameworks. Taken together, these findings suggest that the structural distortion not only promotes photogenerated Ti^3+^ centers through enhanced LMCT but also increases the local polarizability of Ti^4+^ sites by shifting the LUCO toward a greater metal character. This electronic modulation may explain the stronger CO_2_-framework interactions observed in the distorted analogues.

Altogether, these results confirm that Ti^4+^ is the only redox-active metal in the MUV-10­(M) frameworks. The alkaline-earth cations are closed-shell d^0^ metals and cannot participate in photoredox processes. Their role is to introduce structural strain, which increases LMCT efficiency and enhances the formation of photogenerated Ti^3+^ centers upon distortion.

### Boosting CO_2_ Methanation through Cluster Strain

We next evaluated the photocatalytic performance of the MUV-10­(M) series in the hydrogenation of gaseous CO_2_ under simulated sunlight. This demanding eight-electron reduction, which requires both efficient charge separation and thermal activation,^39^ offers a suitable platform to investigate how structural distortion affects charge separation and catalytic efficiency. To enable direct comparison with literature,^40–43^ we employed standardized reaction conditions: photodeposition of RuO_*x*_ nanoparticles (1 wt % Ru) onto polycrystalline MUV-10­(M), and catalytic tests performed with a 4:1 H_2_:CO_2_ gas mixture at 1.5 bar in a quartz photoreactor heated to 200 °C. Upon thermal equilibration, the system was continuously irradiated with simulated sunlight (150 W Hg–Xe lamp, AM 1.5G filter, 450 mW·cm^–2^), and reaction progress was monitored by gas chromatography (GC) analysis of the gas phase. Control reactions using pristine MOFs yielded less than 15 μmol·g^–1^ CH_4_ after 22 h.

As shown in Figure 5a, RuOx@MUV-10­(Sr) exhibited the highest photocatalytic activity, outperforming its Ca and Ba counterparts. Characterization by SEM, PXRD, EDX, XPS, TEM and ICP-MS (Supplementary Section S9) confirmed the formation of small plasmonic RuO_2_ nanoparticles (0.84 ± 0.09 nm; 1 wt % Ru) in all samples, indicating that the framework composition, rather than Ru loading or dispersion, is the critical factor in determining performance. Complementary EPR measurements confirmed that Ti^3+^ species are not generated during RuO_*x*_ photodeposition but are only generated upon light irradiation of the MOFs (Figure S27). RuO_*x*_@MUV-10­(Sr) reached a CO_2_ conversion of 68% after 22 h, with high selectivity to CH_4_ (99.3%; 28.5 mmol·g^–1^) and minor C_2_H_6_ formation (0.7%). Under identical conditions in the dark, CH_4_ production was halved (13.8 mmol· g^–1^), confirming the photocatalytic nature of the process. The consistency of these results was confirmed using a second RuO_*x*_@MUV-10­(Sr) sample prepared from an independent batch, which showed matching structural features and photocatalytic performance (Figure S41). Control experiments using pristine MUV-10­(M) solids further confirmed that the observed activity trend is intrinsic to the framework (Figure S42). A similar activity profile was also observed for nanosized MUV-10­(Sr) samples, ruling out the effect of crystal morphology over performance (Figure S44). A complementary reaction using ^13^CO_2_ confirmed that ^13^CH_4_ (*m*/*z* = 17) originates solely from the gaseous feed, as confirmed by GC–MS (Figure S30a), with no contribution from MOF decomposition. XPS analysis of the spent RuO_*x*_@MUV-10­(Sr) catalyst revealed a partial reduction of Ru^4+^ to metallic Ru^0^ under photocatalytic conditions. *In situ* XPS under H_2_ at 200 °C confirmed this behavior. To assess the impact of RuO_*x*_ reduction on charge separation efficiency, we performed transient photocurrent and electrochemical impedance spectroscopy (EIS) measurements on postreaction samples. RuO_*x*_@MUV-10­(Sr) displayed the highest photocurrent density (Figure 5b) and lowest charge transfer resistance (Figure S25) across the series, followed by the Ba and Ca analogues, in agreement with the observed activity trend.

**Figure 5:**
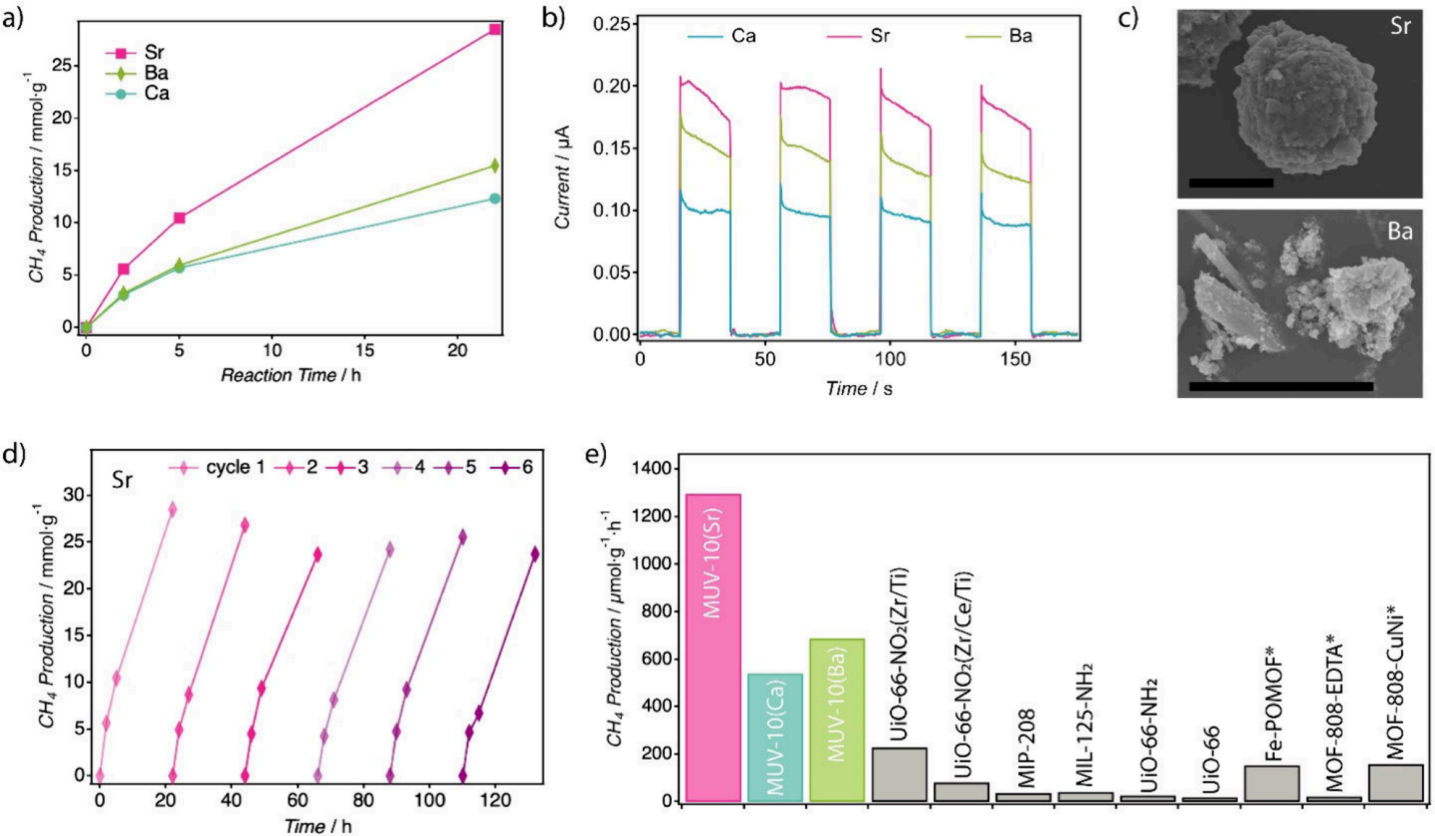
Photocatalytic CO_2_ methanation in the MUV-10­(M) series. a) Time-dependent CH_4_ production over RuO_*x*_@MUV-10­(M) photocatalysts (M = Ca, Sr, Ba) under simulated sunlight irradiation at 200 °C. b) Transient photocurrent responses in postcatalytic samples indicating photoinduced charge separation efficiency, in agreement with photocatalytic trends. c) SEM images of postreaction RuO_*x*_@MUV-10­(Sr) and RuO_*x*_@MUV-10­(Ba), highlighting partial structural degradation in the latter (scale bars: 10 μm). d) Reusability tests for RuO_*x*_@MUV-10­(Sr) over six consecutive CH_4_ production cycles. e) Benchmark comparison of CH_4_ production rates selected MOF-based photocatalysts under comparable conditions (see Table S4 for full data set and references), showing the superior performance of the strained MUV-10­(Sr) analogue. Entries marked with an asterisk (*) correspond to systems employing different cocatalysts and/or sacrificial agents.

The low activity observed for RuO_*x*_@MUV-10­(Ba) cannot be attributed to structural or electronic differences, as these remain comparable to the also-strained Sr analogue based on the experimental and computational data shown above. The origin of this discrepancy instead appears to lie in the lower stability of the Ba analogue. SEM and PXRD analyses revealed partial degradation of MUV-10­(Ba) after catalysis (Figure 5c and Section S9.3). Independent incubation of MUV-10­(Ba) in water led to similar degradation after 24 h, suggesting that the larger ionic radius of Ba^2+^ introduces excessive cluster strain, which compromises framework integrity. Additional tests confirmed that only MUV-10­(Ba) undergoes morphological degradation and Ba leaching upon exposure to ambient air or water, in contrast to the Ca and Sr analogues (Figure S43). This trend is consistent with the tolerance factor analysis (Figure 3b, top), and mirrors the behavior observed in distorted perovskite oxides, where oversized A-site cations are known to destabilize the lattice.^36^ This combination of strain and intrinsically weaker Ba–O bonds, due to the larger ionic radius and higher polarizability of Ba^2+^, makes them more prone to hydrolytic cleavage.

Cycling experiments confirmed the stability and reusability of RuO_*x*_@MUV-10­(Sr). PXRD and TEM (Figures S33 and S40) analyses revealed no structural degradation over six consecutive runs, each yielding >20 mmol·g^–1^ of CH_4_ after 22 h (Figure 5d). The material achieves a production rate of 1.26 mmol·g^–1^·h^–1^, with activity enhancements ranging from 4- to 50-fold relative to previously reported RuO_*x*_-based MOFs such as UiO-66­(Zr/Ti)-NO_2_ ^41^ and MIP-208­(Ti),^22^ under comparable conditions (Figure 5e, Table S7). Apparent quantum yields (AQY) of 1.32% at 400 nm and 0.87% at 600 nm were recorded for RuO_*x*_@MUV-10­(Sr), outperforming leading MOF photocatalysts such as RuO_*x*_@UiO-66­(Zr/Ti)-NO_2_ (0.25% and 0.01%, respectively, under the same conditions).^41^ To the best of our knowledge, strained MUV-10­(Sr) displays the highest reported activity for CO_2_-to-CH_4_ conversion among MOFs, confirming how cluster strain can translate to enhanced catalytic performance.

## Conclusion

This study demonstrates a proof-of-concept for cluster strain engineering in titanium–organic frameworks. By introducing controlled cation-size mismatch into the MUV-10 family, we create local distortions in Ti_2_M_2_ clusters that modulate the Ti–O geometry, enhance ligand-to-metal charge transfer, and facilitate the photogeneration of Ti^3+^ centers. Photocatalytic CO_2_ methanation, used here as a test reaction under standardized conditions, illustrates how these structural effects can be translated into reactivity.

More broadly, compared to conventional approaches for tuning MOF photoreactivity,^29,44,45^ this work shows that cluster strain is an unexplored structural variable to influence metal reactivity by modulating their local coordination environment. Rather than altering light absorption or band alignment, this approach directly addresses the kinetic bottleneck of LMCT. Importantly, the observed distortions follow the trend anticipated from the Goldschmidt tolerance factor,^46^ a classical metric in perovskite chemistry here repurposed to rationalize strain in a molecular framework. This conceptual link bridges oxide catalysis and reticular chemistry, highlighting cluster strain as a potential structural switch for redox activation and as a new design variable for controlling framework reactivity.

## Supplementary Material



## Notes

Supporting Information includes additional experimental details, materials and methods, characterization data, figures referenced in the main text, and crystallographic data for the structures for CCDC 2473912 (MUV-10­(Ba)-fresh) and CCDC 2473910 (MUV-10­(Ba)-evacuated). Additional data sets generated and analyzed during the current study are available from the corresponding authors upon reasonable request. (PDF)

## #

Current affilation: Departamento de Química Inorgánica, Universidad de Granada, Avenida Fuentenueva S/N, Granada 18071, Spain
