# Supplementary material for: Inducing Reactivity by Cluster Strain in Titanium Frameworks
Source: J Am Chem Soc. 2025 Dec 24;148(1):734–42. doi: 10.1021/jacs.5c16069 (PMC12814332; doi:10.1021/jacs.5c16069)
Supplement: Supplementary file 1 [file ja5c16069_si_001.pdf]

# Supplementary Information

## Inducing Reactivity by Cluster Strain in Titanium Frameworks

Eloy P. Gómez-Oliveira,[a]$ Vitor Fernandes de Almeida,[b]$ Javier Castells-Gil,[c] Herme G. Baldoví,[b] Felipe Gándara,[d] Neyvis Almora-Barrios,[a,e] Sergio Tatay,[a] Sergio Navalón,[b]* Natalia M. Padial[a,f]* and Carlos Martí-Gastaldo[a]*

[a] Functional Inorganic Materials Team, Instituto de Ciencia Molecular (ICMol), Universitat de València, 46980 València, Spain.

[b] Department of Chemistry, Universitat Politècnica de València. Camino de Vera, s/n, 46022 València, Spain.

[c] School of Chemistry, University of Birmingham. Birmingham, B152TT, United Kingdom.

[d] Instituto de Ciencia de Materiales de Madrid (ICMM-CSIC). Sor Juana Inés de la Cruz 3, 28049 Madrid, Spain.

[e] Current affilation: Departamento de Química Física, Universitat de València. Carrer del Doctor Moliner, 50, 46100 Burjassot, Spain.

[f] Current affilation: Departamento de Química Inorgánica, Universidad de Granada, Avenida Fuentenueva S/N, Granada 18071, Spain.

Table of Contents


***S.1. GENERAL CONSIDERATIONS: REAGENTS AND CHARACTERISATION METHODS............. 4***

*S.1.1. MATERIALS AND REAGENTS ........................................................................... 4*

*S.1.2. PHYSICAL AND CHEMICAL CHARACTERIZATION ........................................................ 5*

***S.2. SYNTHESIS AND CHARACTERIZATION OF THE MUV-10(M) MATERIALS ........................ 7***

*S.2.1. SYNTHESIS OF MUV-10(M) MATERIALS ............................................................. 7*

*S.2.1.1. SYNTHESIS OF MUV-10(Ca) ........................................................................ 7*

*S.2.1.2. SYNTHESIS OF MUV-10(Sr) ........................................................................ 7*

*S.2.1.3. SYNTHESIS OF MUV-10(Ba) ........................................................................ 7*

*S.2.2. CHARACTERIZATION OF MUV-10(Sr) ................................................................ 8*

*S.2.3. CHARACTERIZATION OF MUV-10(Ba) ................................................................ 9*

*S.2.4. COMPARISON OF MUV-10(M) ....................................................................... 10*

*S.2.5. X-RAY PHOTOELECTRON SPECTROSCOPY SPECTRA ................................................. 11*

*S.2.6. $CO_2$ ADSORPTION AND ISOSTERIC ANALYSIS ........................................................ 16*

*S.2.7. LEBAIL REFINEMENT OF MUV-10(M) FAMILY ....................................................... 17*

***S.3. STRUCTURE ANALYSIS ................................................................................. 18***

*S.3.1. MUV-10(Sr) .............................................................................................. 18*

*S.3.2. MUV-10(Ba) .............................................................................................. 20*

*S.3.3. TOLERANCE FACTOR CALCULATIONS ............................................................... 21*

*S.3.4. ANGULAR DISTORTION METRICS ANALYSIS ....................................................... 22*

***S.4. PHOTOCHARACTERIZATION ........................................................................... 23***

*S.4.1. UV-VIS DIFFUSE REFLECTANCE SPECTROSCOPY .................................................. 23*

*S.4.2. EXPERIMENTAL OPTICAL BAND-GAP CALCULATION .............................................. 24*

*S.4.3. ULTRAVIOLET PHOTOELECTRON SPECTROSCOPY SPECTRA. ..................................... 25*

***S.5. PHOTODEPOSITION OF RUTHENIUM ................................................................. 26***

*S.5.1. PHOTODEPOSITION METHOD ........................................................................ 26*

*S.5.2. CHARACTERIZATION AFTER DEPOSITION .......................................................... 27*

***S.6. PHOTOCHARACTERIZACION AFTER Ru PHOTODEPOSITION ..................................... 28***

*S.6.1. X-RAY PHOTOELECTRON SPECTROSCOPY SPECTRA ................................................. 28*

*S.6.2. TRANSMISION ELECTRONIC MICROSCOPY ......................................................... 33*

*S.6.3. UV-VIS DIFFUSE REFLECTANCE SPECTROSCOPY ........ 36*

*S.6.4. PHOTOCURRENT EXPERIMENTS........ 37*

*S.6.5. NYQUIST PLOT ........ 38*

***S.7. COMPUTATIONAL METHODS ........ 39***

*S.7.1. ELECTRONIC STRUCTURE OF MUV-10(X) ........ 39*

***S.8. ELECTRON PARAMAGNETIC RESONANCE (EPR) ........ 40***

***S.9. PHOTOCATALYSIS EXPERIMENTS ........ 42***

*S.9.1.1 METHODOLOGICAL DETAILS ........ 42*

*S.9.1.2 PRODUCT ANALYSIS ........ 42*

*S.9.2. PHOTOCATALYTIC SABATIER REACTION RESULTS........ 43*

*S.9.3. CHARACTERIZATION AFTER PHOTOCATALYTIC EXPERIMENTS........ 44*

*S.9.4. REUSES CYCLES........ 45*

*S.9.5. XPS BEFORE AND AFTER PHOTOCATALYTIC SABATIER REACTION........ 47*

*S.9.6. IN SITU XPS BEFORE AND AFTER $H_2$ TREATMENT........ 50*

*S.9.7. TRANSMISION ELECTRONIC MICROSCOPY AFTER REUSES CYCLES........ 53*

*S.9.8. REPRODUCIBILITY OF RuOx PHOTODEPOSITION AND PHOTOCATALYTIC PERFORMANCE ........ 54*

*S.9.9. PHOTOCATALYTIC ACTIVITY OF PRISTINE MUV-10(M) FRAMEWORKS. ........ 55*

*S.9.10. STABILITY TESTS IN AIR AND WATER. ........ 56*

*S.9.11. PARTICLE SIZE EFFECTS ON MUV-10(Sr) PHOTOCATALYTIC PERFORMANCE........ 57*

***S.10. BENCHMARKING OF PHOTOCATALYTIC $CO_2$ METHANATION PERFORMANCE ........ 58***

***S.11. REFERENCES ........ 59***

# S.1. GENERAL CONSIDERATIONS: REAGENTS AND CHARACTERISATION METHODS

## S.1.1. MATERIALS AND REAGENTS

All purchased reagents and solvents were used without any previous purification. Commercially available chemicals were obtained from Alfa Aesar, BLDpharm, Merk, and Scharlab. 1,3,5-benzenetricarboxylic acid (98%) were purchased from Alfa Aesar. 4-*tert*-butylbenzoic acid (4-tbbz, >99%), calcium (II) chloride anhydrous powder ($CaCl_2$, ≥ 96.0%), titanium (IV) isopropoxide ($Ti(O^iPr)_4$, 97.0%), bis(cyclopentadienyl)titanium(IV) dichloride ($Cp_2TiCl_2$, 97%), potassium perrhuthenate ($KRuO_4$, ≥ 49%), lithium perchlorate ($LiClO_4$, 99.99%), acetonitrile ($CH_3CN$, ≥ 99.9%) and terpineol ($C_{10}H_{18}O$, ≥ 99.5%) were purchased from Merk. *N,N*-dimethylformamide (DMF, ≥ 99.8%), acetone (≥ 99.8%), methanol (MeOH, ≥ 99.8%), 2-propanol ($^iPrOH$, >99%), tetrahydrofuran (THF, ≥ 99.8%) and acetic acid ($CH_3COOH$, 99.7%) were purchased from Scharlab.

The synthesis of $[Ti_6O_6(O^iPr)_6(4\text{-tbbz})_6]$ (4-tbbz = 4-tert-butylbenzoic acid; $Ti_6$ cluster) was carried out according to a previously reported procedure.[1]

## S.1.2. PHYSICAL AND CHEMICAL CHARACTERIZATION

For the characterization of MUV-10(M) (M = Ca, Sr, Ba), each sample was characterized by PXRD, SEM-EDX, TGA, Gas Adsorption and ICP-MS:

**SEM-EDX:** Scanning Electron microscopy and single point energy-dispersive X-Ray analysis (EDX). Analysis was performed with a Hitachi S-4800 scanning electron microscope at an accelerating voltage of 20 keV, over metalized samples with a mixture of gold and palladium for 90 seconds. Mapping of Ti and M (M = Ca, Sr, Ba) confirm that metal distribution is homogeneous throughout the crystals.

**Routine PXRD patterns** were collected in a PANanlytical X'Pert PRO diffractometer using cooper radiation (Cu $K_\alpha$ = 1.5418 Å) with an X'Celerator detector, operating at 45 kV and 40 mA. Profiles were collected by using a Soller Slit of 0.04° and a divergence slit of 1/2 at room temperature in the 1.5°< 2θ < 40° range with a step size of 0.017°.

**Powder X-Ray Diffraction (XDR) patterns for LeBail refinement** were collected for polycrystalline samples using a 0.5 mm glass capillary mounted and aligned in a PANanlytical Empyrean diffractometer using copper radiation (Cu Kα = 1.5418 Å) with an PIXcel detector, operating at 40 mA and 45 kV. Profiles were collected by using a Soller Slit of 0.02° and a divergence slit of ¼ at room temperature in the angular range 1.5° < 2θ <60° range with a step size of 0.013°. LeBail profile fitting were carried out with TOPAS Academic v6 (http://www.topas-academic.net/).[2]

**Powder X-Ray Diffraction (XDR) patterns for Rietveld structural refinement** were collected for polycrystalline samples using a 0.5 mm glass capillary mounted and aligned in MSPD BL-04 of ALBA Synchrotron using a fixed wavelength (λ = 0.70874 Å). Profiles were collected at room temperature in the angular range 0.80°< 2θ < 60° with a step size of 0.006°. Rietveld structural refinements were carried out with TOPAS Academic v7 (http://www.topas-academic.net/).[2] Prior to Rietveld refinement, a full profile powder refinement was carried out with the LeBail method in all the samples to confirm the space group and phase purity. For the Rietveld refinement of MUV-10(Sr), we used the crystallographic model of MUV-10(Ba). The organic BTC linker was refined as a rigid body with initial bond distances were set as 1.38 Å (aromatic C-C), 1.45 Å (exocyclic C-C), 1.28 Å (carboxylic C-O), and H atoms were placed in ideal positions with a bond distance constrain of 0.98 Å (C-H). All the constrained bond distances were allowed to refine with the exception of the C-H bond with was fixed throughout the refinement. The residual electron density inside the pores was modelled with acetone (refined as rigid body) and water molecules, whose position and occupancies were allowed to refine throughout the refinement. The background was fitted with a 18-coefficient Chebyshev polynomial and peak-shapes were modelled with a Thompson-Cox- Hasting pseudo-Voigt profile function. The instrumental parameters were obtained from the measurement of a silicon standard.

**$N_2$ Gas Adsorption:** Surface area, pore size and volume values were calculated from nitrogen adsorption-desorption isotherms (77 K) recorded on a Micromeritics 3Flex apparatus. Samples were degassed overnight at 100 °C and $10^{-6}$ Torr prior to analysis. Brunauer-Emmett-Teller (BET) Surface area analysis were performed as recommended for microporous and mesoporous materials (see below).[3] Specific surface area (SA) was calculated by multi-point Brunauer-Emmett-Teller (BET) method. Total pore volume was taken at $P/P_0$=0.96. Pore size distribution was analyzed by using the Oxide Surface method for the micropore distribution and Multi-Walled NanoTubes (MWNT) method for the mesopore distribution, both methods use the Non-Local Density Functional Theory (NLDFT) for the adsorption branch by assuming a cylindrical pore model.

**$CO_2$ Gas Adsorption:** $CO_2$ at 273K, 283K and 298K for calculate thr heat of adsoption were recorded on an Advanced BELSORP MAX II. Samples were degassed overnight at 100 °C and $10^{-6}$ Torr prior to analysis.

**TGA** was carried out with a TGA 550 (Waters/TA Instruments) apparatus between 25 and 650 °C under ambient conditions (5 °C $min^{-1}$ scan rate and an air flow of 90 mL·$min^{-1}$). As-synthesized materials were washed with DMF, MeOH and acetone, and exchanged with acetone for 2 days prior to analysis.

**ICP-MS:** Inductively Coupled Plasma Mass Spectrometry measurements were carried out with an Agilent 7900 apparatus.

**UV-Vis Diffuse Reflectance Spectroscopy (DRS) measurements** were performed on a Jasco V-670 spectrophotometer using an integrated Labsphere in the range 200-800 nm.

**EPR spectra** were collected in a Bruker ELEXYS E580 spectrometer operating in X-band (~9.3 GHz) at 77 K.

**XPS spectra** were recorded using a SPECS spectrometer equipped with an MCD-9 detector using an Al X-ray source ($K_\alpha$= 1486.6 eV). The C 1s peak at 284.4 eV was set as reference binding energy. CASA software has been employed for spectra deconvolution.

**UPS spectra** were acquired using a SPECS GmbH photoelectron spectroscopy instrument equipped with an ultrahigh vacuum system and an energy analyzer PHOIBOS 150 9MCD was employed. He I (21.2 eV) was employed as photon source and with a pass energy of 2 eV.

**Electrochemical impedance spectroscopy (EIS) and photocurrent measurements** were carried out using a Gamry Instruments potentiostat (model Interface 5000E). A three-electrode cell was employed in a home-made quartz cell. A platinum disc was used as the counter electrode, and an Ag/Ag+ electrode in acetonitrile was used as the reference. The working electrode (WE) consisted of a conductive glass coated with FTO (2.0 x 1.0 cm) containing the MOF-based material deposited on a 0.5 $cm^2$ area. The experiment was conducted using a 0.1 M $LiClO_4$ solution as the electrolyte

# S.2. SYNTHESIS AND CHARACTERIZATION OF THE MUV-10(M) MATERIALS

## S.2.1. SYNTHESIS OF MUV-10(M) MATERIALS

### *S.2.1.1. SYNTHESIS OF MUV-10(Ca)*

Following a reported procedure by our group,[4] the synthesis of MUV-10(Ca) were carried out by dissolving 125.0 mg of benzene-1,3,5-tricarboxylic acid ($H_3$btc, 595 µmol), 26.8 mg of $CaCl_2·6H_2O$ (120 µmol) for MUV-10(Ca) in a mixture of 12 mL of *N,N*-dimethylformamide and 3.5 mL of AcOH in a 25 mL Schott bottle. Subsequently, 36 µL of $Ti(OiPr)_4$ (120 µmol) were added to the clear solution. The bottle was sealed and heated in an oven at 120 °C for 48 hours (heating rate: 5 °C·min$^{-1}$, cooling rate: 0.5 °C·min$^{-1}$). After cooling down to room temperature, the microcrystalline powder was recovered by centrifugation and rinsed with fresh DMF, water and MeOH several times. The solids were then allowed to dry under vacuum at room temperature.

### *S.2.1.2. SYNTHESIS OF MUV-10(Sr)*

The synthesis of MUV-10(Sr) were carried out by dissolving 125.0 mg of benzene-1,3,5-tricarboxylic acid ($H_3$btc, 595 µmol), 31.6 mg of $SrCl_2·6H_2O$ (120 µmol) for MUV-10(Sr) in a mixture of 12 mL of *N,N*-dimethylformamide and 7.5 mL of AcOH in a 25 mL Schott bottle. Subsequently, 36 mg of $Ti_6$ cluster (120 µmol) were added to the clear solution. The bottle was sealed and heated in an oven at 120 °C for 48 hours (heating rate: 5 °C·min$^{-1}$, cooling rate: 0.5 °C·min$^{-1}$). After cooling down to room temperature, the microcrystalline powder was recovered by centrifugation and rinsed with fresh DMF, water and MeOH several times. The solids were then allowed to dry under vacuum at room temperature.

### *S.2.1.3. SYNTHESIS OF MUV-10(Ba)*

The synthesis of MUV-10(Ba) were carried out by dissolving 125.0 mg of benzene-1,3,5-tricarboxylic acid ($H_3$btc, 595 µmol), 29.5 mg of $BaCl_2·4H_2O$ (120 µmol) for MUV-10(Ba) in a mixture of 12 mL of *N,N*-dimethylformamide and 7.0 mL of AcOH in a 25 mL Schott bottle. Subsequently, 36 mg of $Ti_6$ cluster (120 µmol) were added to the clear solution. The bottle was sealed and heated in an oven at 120 °C for 48 hours (heating rate: 5 °C·min$^{-1}$, cooling rate: 0.5 °C·min$^{-1}$). After cooling down to room temperature, the microcrystalline powder was recovered by centrifugation and rinsed with fresh DMF, water and MeOH several times. The solids were then allowed to dry under vacuum at room temperature.

**Table S1.** Elemental analysis of pristine MUV-10(M) materials.

| MOF | C (%) | | H (%) | | N (%) | |
|---|---|---|---|---|---|---|
| | Theor. | Exp. | Theor. | Exp. | Theor. | Exp. |
| MUV-10(Ca) | 34.6 | 33.8 ± 0.31 | 1.92 | 2.04 ± 0.15 | 0 | 1.02 ± 0.08 |
| MUV-10(Sr) | 31.1 | 30.3 ± 0.23 | 1.73 | 1.81 ± 0.12 | 0 | 0.87 ± 0.07 |
| MUV-10(Ba) | 28.0 | 26.9 ± 0.19 | 1.56 | 1.67 ± 0.09 | 0 | 1.04 ± 0.07 |

## S.2.2. CHARACTERIZATION OF MUV-10(Sr)

a)

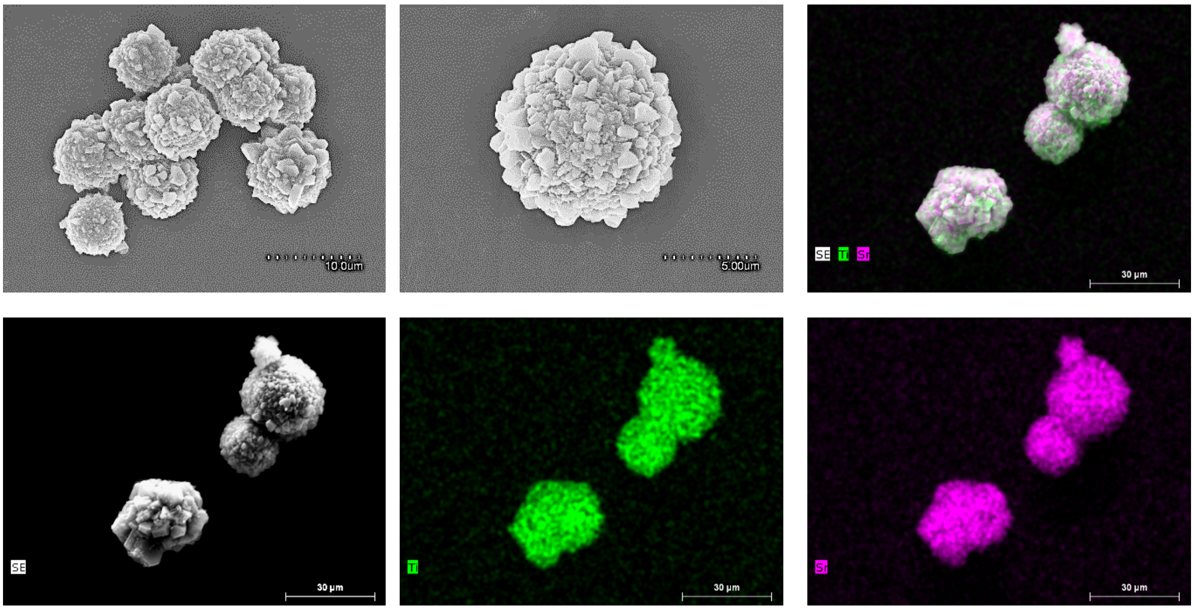


b)

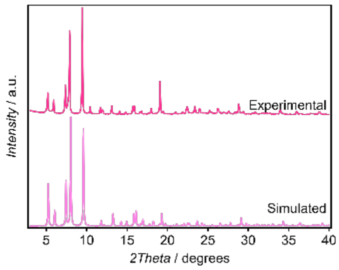


c)

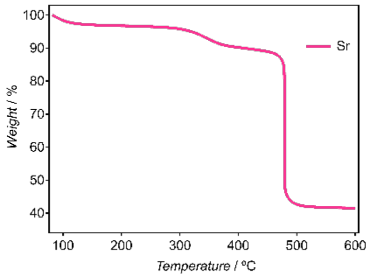


d)

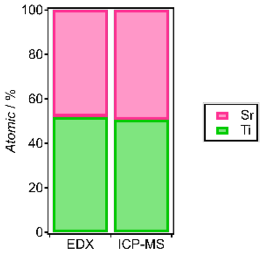


e)

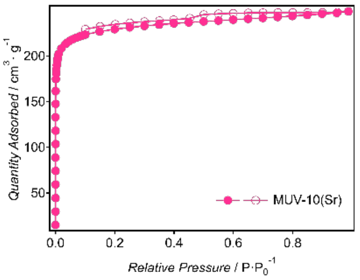


f)

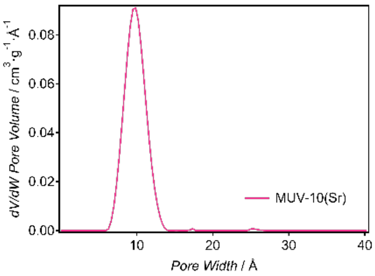


g)

| | |
|---|---|
| **BET Area ($m^2 \cdot g^{-1}$)** | 923.4 ± 2.2 |
| **Slope (g/cm³)** | 0.004713 ± 0.000011 |
| **Intercept (g/cm³)** | 0.000001 ± 0.000000 |
| **$R^2$** | 0.9999503 |
| **C** | 10417.498616 |
| **$V_m$ ($cm^3 \cdot g^{-1}$)** | 212.1589 |
| **$1/(\sqrt{C}+1)$** | 0.0097971 |

**Figure S01.** (top) Scanning Electron Microscopy (SEM) and Mapping showing Ti (green) and Sr (pink); a) Comparison of experimental and simulated PXRD measurements; b) TGA analysis; c) Comparison of metal content determined by EDX analysis and ICP-MS showing Ti (green) and Sr (pink); d) $N_2$ adsorption isotherm at 77 K; e) Pore Size Distribution calculated by Oxide Surface method (regularization = 0.1); f) main parameters calculated from the multi-point BET analysis.

## S.2.3. CHARACTERIZATION OF MUV-10(Ba)

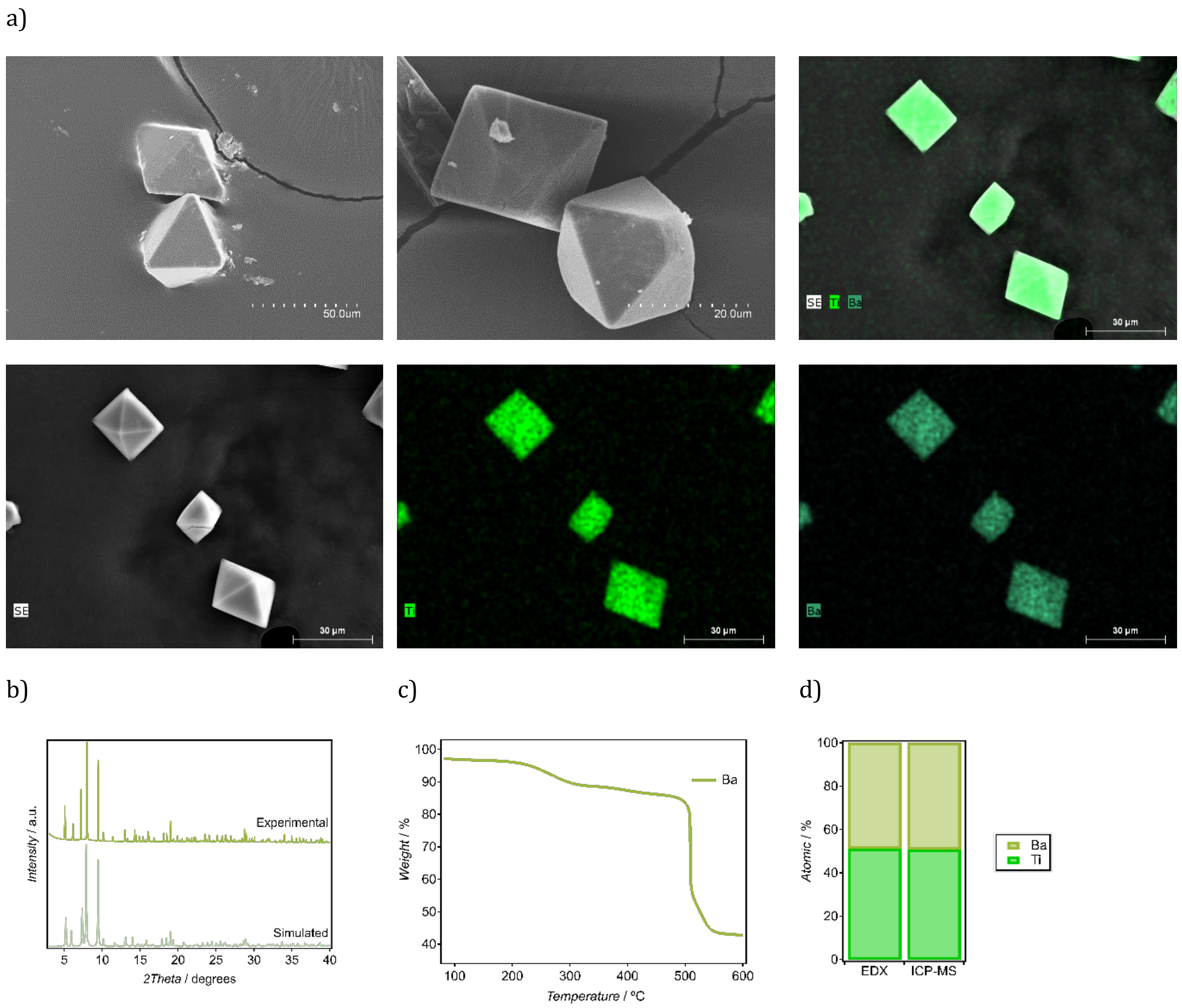


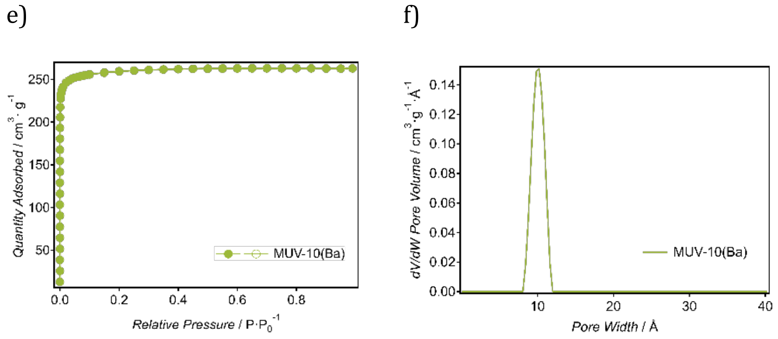


g)

| | |
|---|---|
| **BET Area ($m^2 \cdot g^{-1}$)** | 949.4 ± 1.4 |
| **Slope ($g/cm^3$)** | 0.004584 ± 0.000007 |
| **Intercept ($g/cm^3$)** | 0.000001 ± 0.000000 |
| **$R^2$** | 0.9999767 |
| **C** | 17265.110611 |
| **$V_m$ ($cm^3 \cdot g^{-1}$)** | 218.1280 |
| **$1/(\sqrt{C}+1)$** | 0.0076103 |

**Figure S02.** a) Scanning Electron Microscopy (SEM) and Mapping showing Ti (green) and Ba (light green); b) Comparison of experimental and simulated PXRD measurements; c) TGA analysis; d) Comparison of metal content determined by EDX analysis and ICP-MS showing Ti (green) and Ba (light green); e) $N_2$ adsorption isotherm at 77 K; f) Pore Size Distribution calculated by Oxide Surface method (regularization = 0.1); f) main parameters calculated from the multi-point BET analysis.

## S.2.4. COMPARISON OF MUV-10(M)

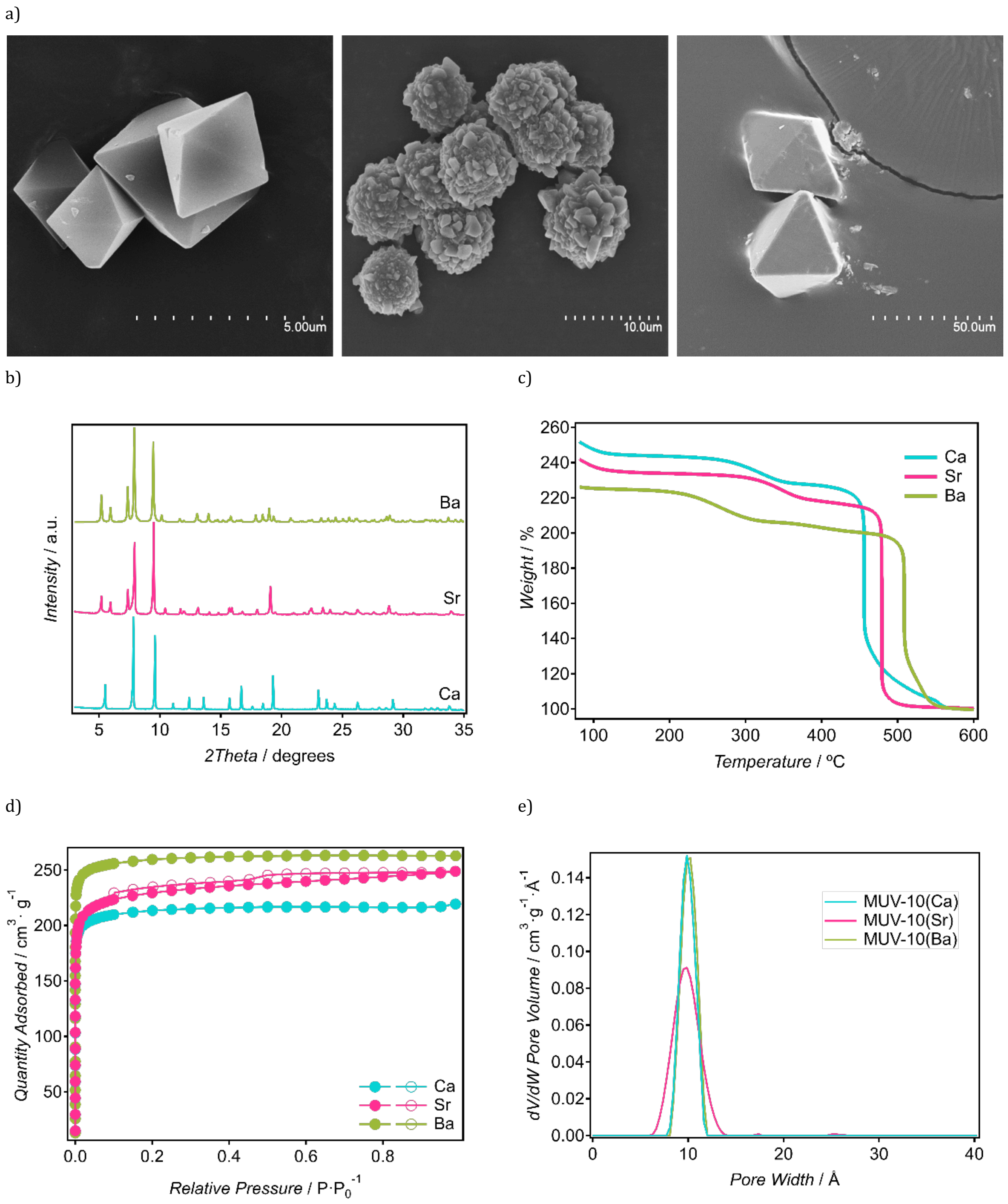


**Figure S03.** Summary and comparison of the characterization of MUV-10(M) family materials: a) Scanning Electron Microscopy (SEM) images of Ca (left), Sr (middle), and Ba (right); b) routine PXRD measurements; c) Thermo-Gravimetric Analysis; d) $N_2$ adsorption isotherms; e) Pore Size Distribution.

## S.2.5. X-RAY PHOTOELECTRON SPECTROSCOPY SPECTRA

MUV-10(Ca), MUV-10(Sr) and MUV-10(Ba) solids were characterized by XPS. C 1s XPS region shows characteristic features of trimesic organic ligand within MUV network, a main band at 284.4 eV due to aromatic C-C $sp^2$ bonds accompanied by a smaller band at 288.1 eV associated with carboxylate groups.[5] O 1s XPS region is associated to the presence of oxygen atoms present in carboxylate ligands (531.1 eV) together with metal-oxygen bonds (529.9 eV).[5] Ti 2p XPS shows the presence of two bands at about 458.4 and 463.7 eV attributable to Ti $2p_{3/2}$ and Ti $2p_{1/2}$ of Ti(IV), respectively, in MUV.[5] In the case of MUV-10(Ca), Ca 1s XPS region reveal the presence of two bands at 346.8 and 350.4 eV due to Ca $2p_{3/2}$ and $2p_{1/2}$ of Ca(II), respectively.[6] In the case of MUV-10(Sr), Sr XPS region can be deconvoluted into two bands at 133.1 and 134.7 eV due to Sr(II) species.[7] In the case of MUV-10(Ba), Ba XPS region shows two bands at 780.0 and 795.3 associated to Ba $3d_{5/2}$ and $3d_{3/2}$ of Ba(II) species, respectively.[8]

MUV-10(Ca)

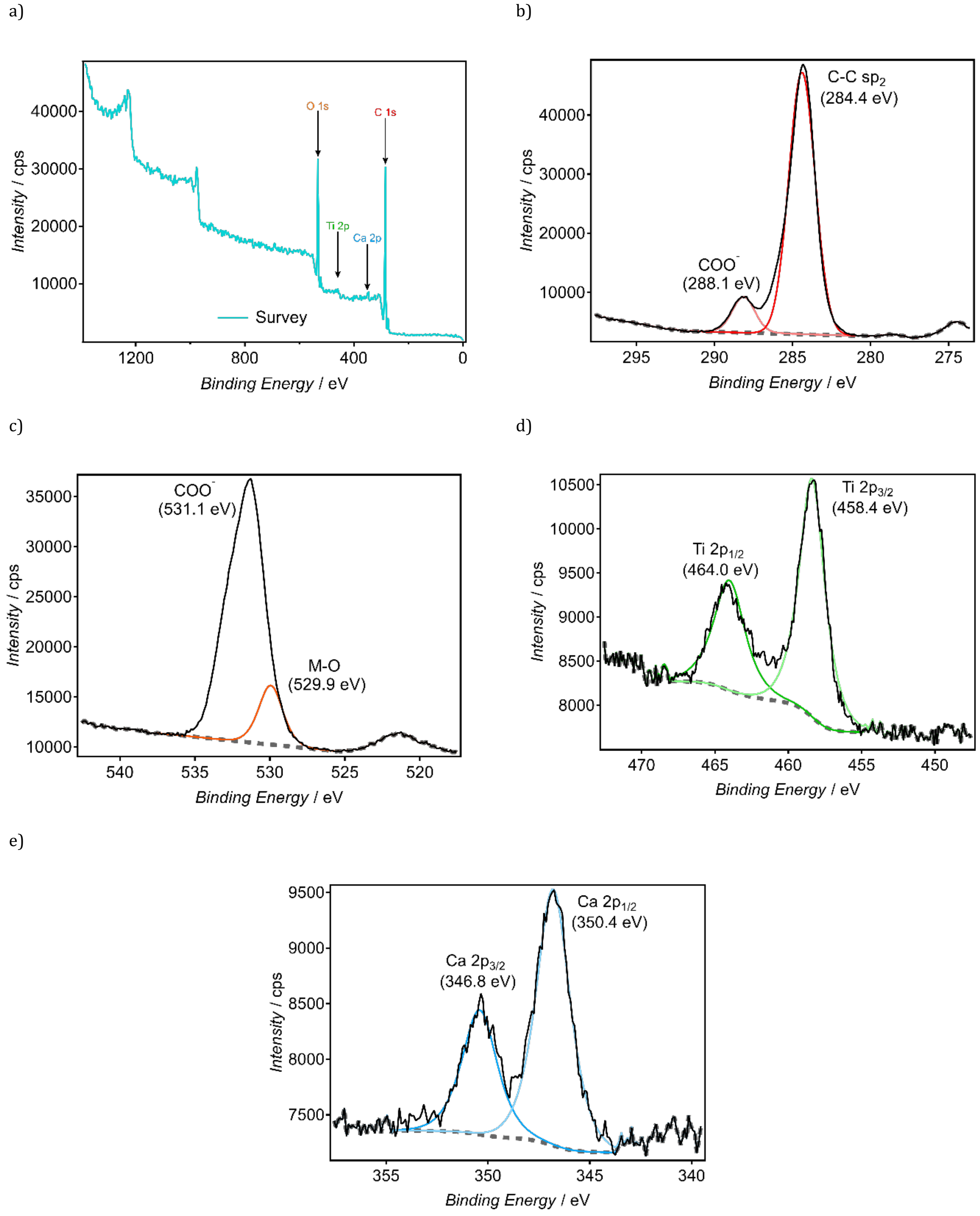


**Figure S04**. XPS a) Survey, b) C 1s, c) O 1s, d) Ti 2p, e) Ca 2p of MUV-10(Ca).

MUV-10(Sr)

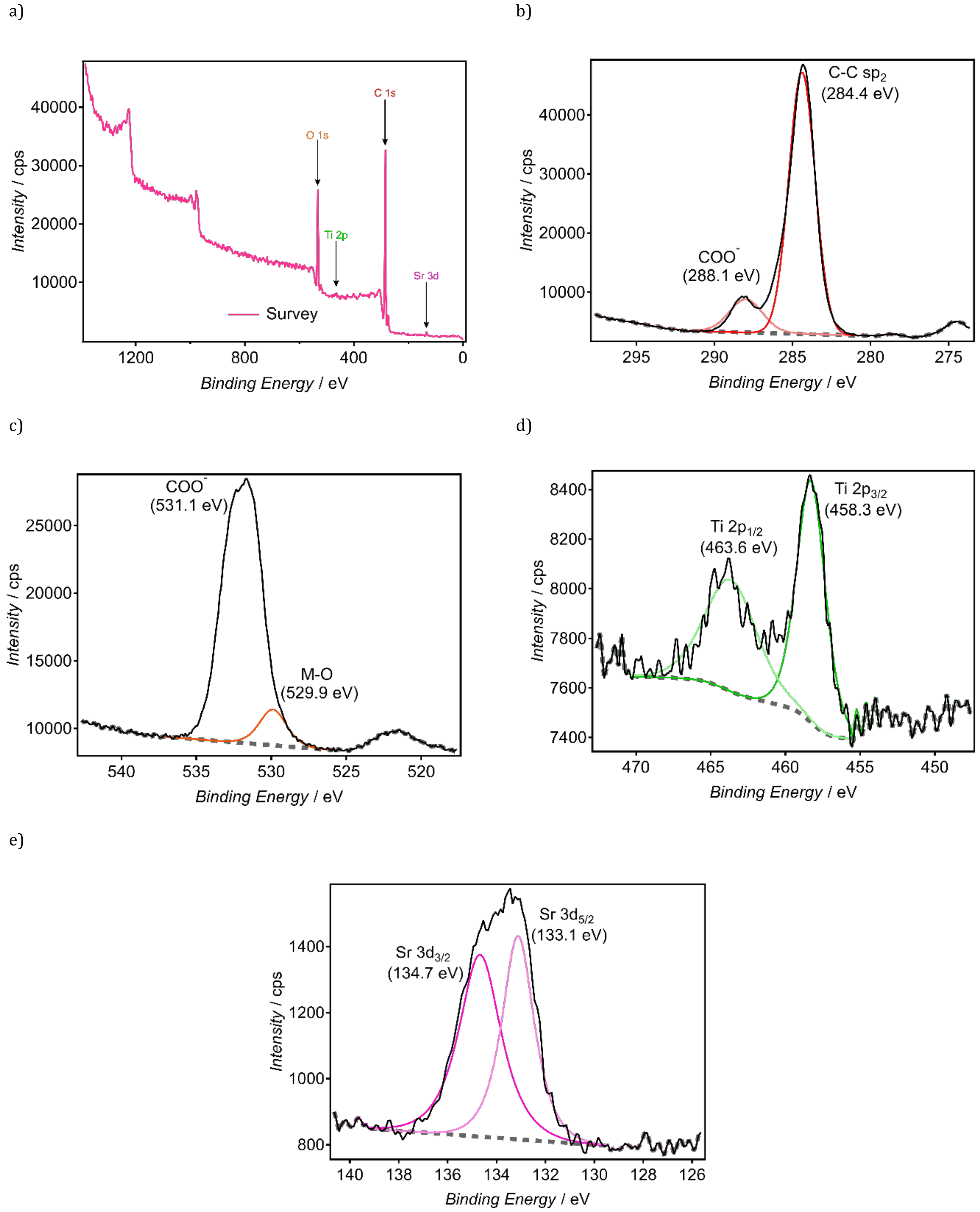


**Figure S05**. XPS a) Survey, b) C 1s, c) O 1s, d) Ti 2p, e) Sr 3d of MUV-10(Sr).

MUV-10(Ba)

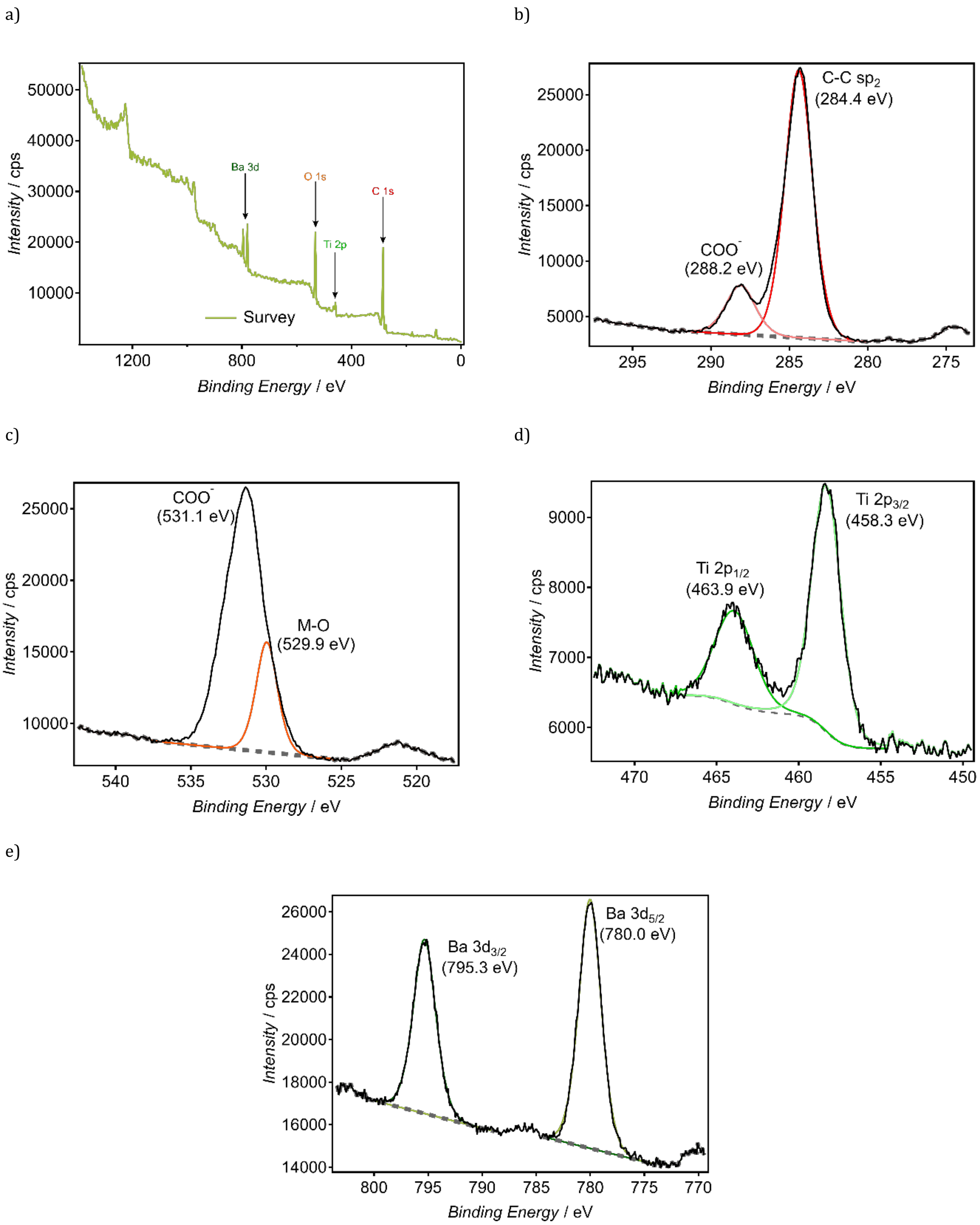


**Figure S06.** XPS a) Survey, b) C 1s, c) O 1s, d) Ti 2p, e) Ba 3d of MUV-10(Ba).

Comparison of MUV-10(M) (M = Ca, Sr or Ba) pristine materials.

a)

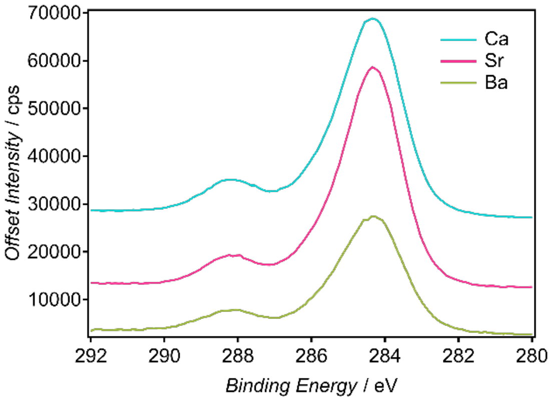


b)

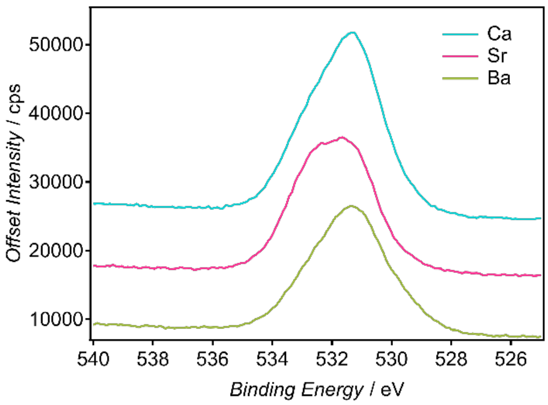


c)

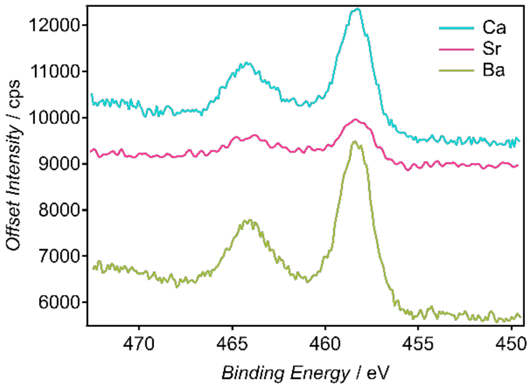


**Figure S07.** Comparison of XPS a) C 1s, b) O 1s, and c) Ti 2p, zones of MUV-10(M) pristine materials (M = Ca, Sr or Ba).

## S.2.6. $CO_2$ ADSORPTION AND ISOSTERIC ANALYSIS

The process to calculate adsorption heat from Clausius-Clapeyron equation is as follows. At least 2 different adsorption isotherms that were measured at different temperatures T1 and T2 are needed for the analysis. Qst at an adsorption amount can be calculated from the equation below with the difference between the 2 different pressures at the same adsorption amount. $Qst = \frac{R \cdot T1 \cdot T2}{T2 - T1} \cdot (\ln P2 - \ln P1)$. [9]

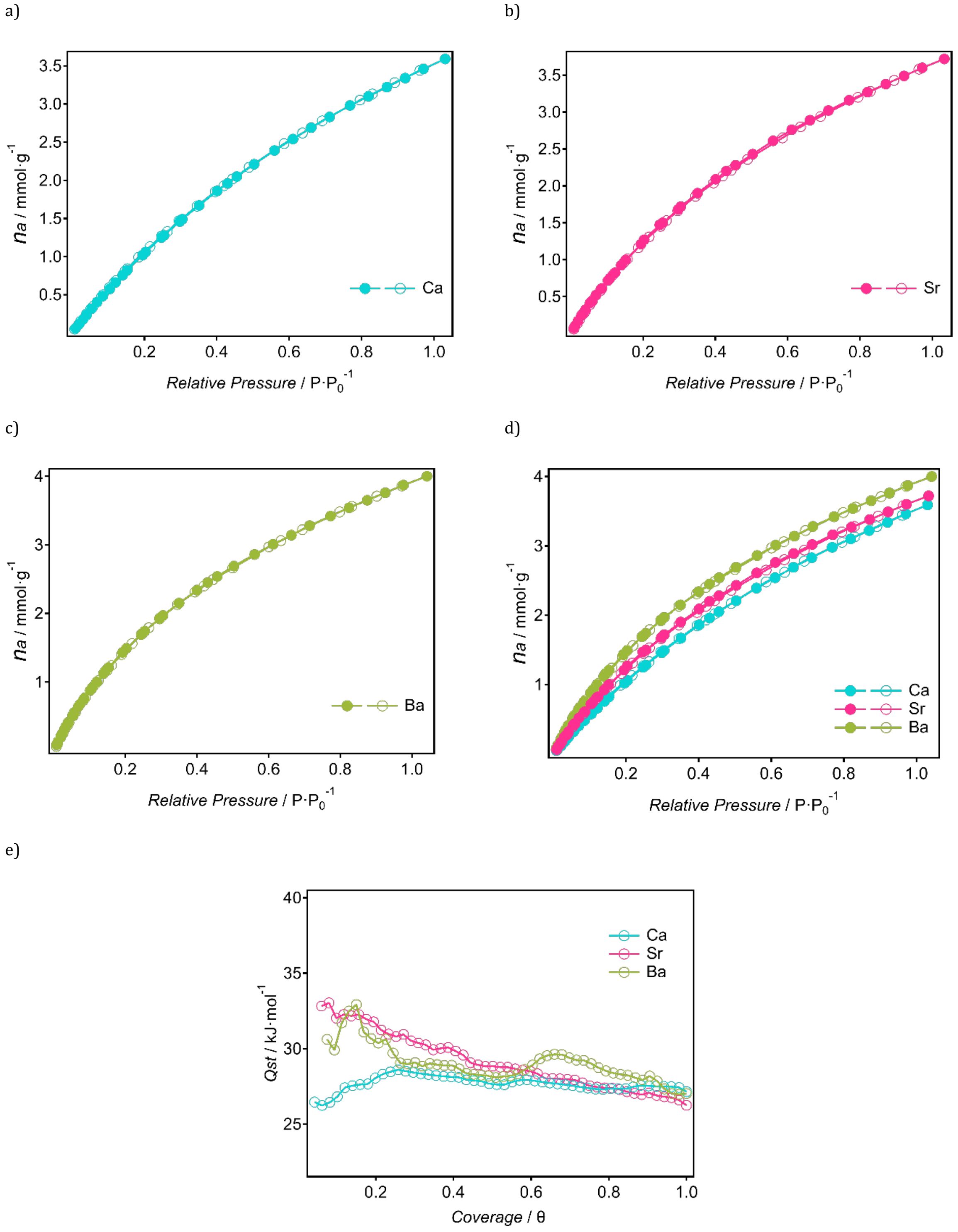


**Figure S08.** $CO_2$ at 298K adsorption measurements of MUV-10(M) family materials: a) Calcium, b) Strontium, c) Barium; d) Comparison of $CO_2$ adsorption at 298K; e) Heat of Adsorption of MUV-10(M) family calculated from $CO_2$ adsorption isotherms at 273K, 283K and 298K.

## S.2.7. LEBAIL REFINEMENT OF MUV-10(M) FAMILY

a)

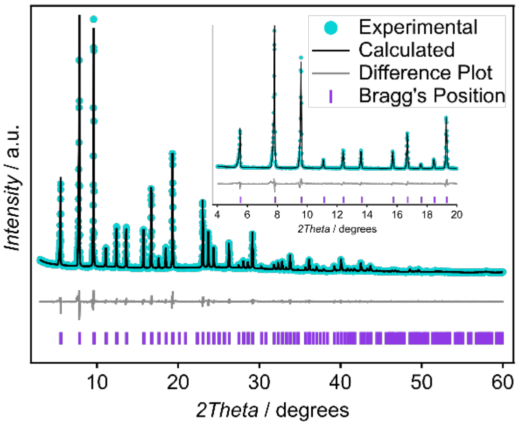


b)

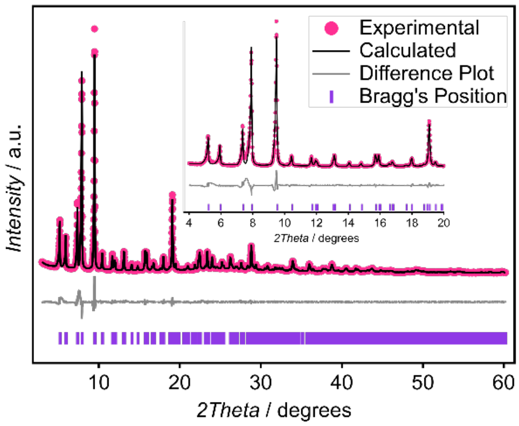


c)

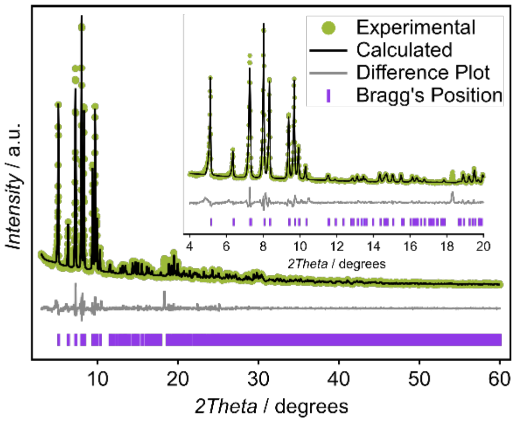


d)

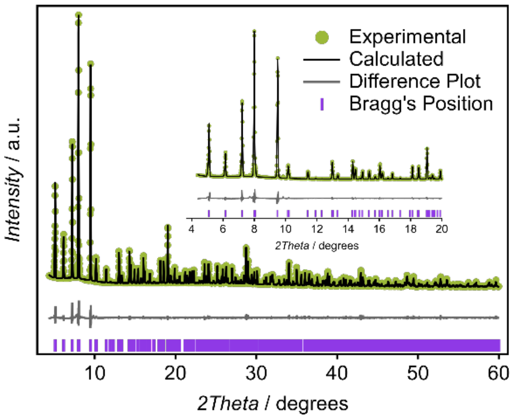


e)

| Material | a (Å) | b (Å) | c (Å) | a = β = γ (º) | Volume (Å$^3$) | Space Group | r_wp / r_exp (%) |
|---|---|---|---|---|---|---|---|
| **MUV-10(Ca) (a)** | 15.8889 (1) | | | 90 | 4011 | Pm-3 (200) | 5.63 / 3.34 |
| **MUV-10(Sr) (b)** | 23.8492 (13) | | 14.7601 (12) | 90 | 8496 | P4/mbm (127) | 7.73 / 3.28 |
| **MUV-10(Ba) Solvated (c)** | 24.0213 (18) | | 29.7346 (18) | 90 | 17153 | $P4_2$/mnm (136) | 6.39 / 4.67 |
| **MUV-10(Ba) Evacuated (d)** | 24.4815 (12) | | 14.3712 (13) | 90 | 8613 | P4/mbm (127) | 4.19 / 2.33 |

**Figure S09.** Plots of the LeBail refinements of MUV-10(M) family materials for: a) Calcium, b) Strontium, c) Barium solvated and d) Barium evacuated. Finally, e) Summary table with the most important parameters and errors of LeBail refinements.

## S.3. STRUCTURE ANALYSIS

### S.3.1. MUV-10(Sr)

**Table S2.** Crystallographic Information of MUV-10(Sr).

| Name | MUV-10(Sr) |
|---|---|
| **Empirical formula** | $[Ti_3Sr_3(\mu\text{-}O)_3(C_9H_3O_6)_4(H2O)_4]$ $20.22H_2O\cdot 2.24(C_3H_6O)$ |
| **Formula weight, g mol$^{-1}$** | 1849.1 |
| **Temperature, K** | 303 |
| **Crystal system** | Tetragonal |
| **Space group** | P4/mbm |
| **a, Å** | 23.7631(9) |
| **c, Å** | 14.7280(7) |
| **Volume, Å3** | 8316.7(8) |
| **Z** | 4 |
| **Number of structural variables/Number of total variables** | 65/85 |
| **Wavelength, Å** | 0.70874 |
| **2θ range, °** | 1.5 - 30 |
| **Rp, %** | 1.59 |
| **Rwp, %** | 2.73 |
| **Rexp, %** | 0.19 |
| **RBragg, %** | 1.12 |
| **GoF** | 14.70 |

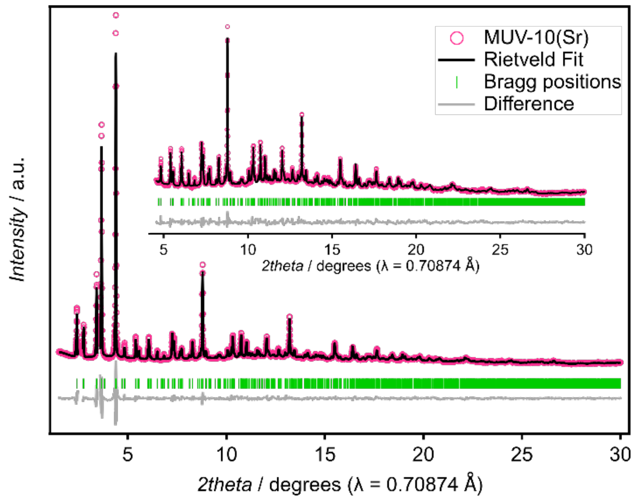


**Figure S10.** Rietveld refinement plot of MUV-10(Sr).

**Table S3.** Refined fractional atomic coordinates, isotropic displacement parameters ($B_{iso}$)**and site occupancy factors (Occ)** from the Rietveld refinement of **MUV-10(Sr)**

| Atom | x | y | z | Biso / Å$^2$ | Occ |
|---|---|---|---|---|---|
| **Sr1A** | 0.7448 (6) | 0.2448 (6) | 0.3586 (9) | 4.7 (3) | 0.5 |
| **Ti1A** | 0.6526 (12) | 0.3404 (12) | 0.5 | 1.0 (4) | 0.5 |
| **O1A** | 0.712 (3) | 0.301 (3) | 0.5 | 1.6 (6) | 0.5 |
| **O1Wa** | 0.588 (3) | 0.412 (3) | 0.5 | 1.6 (6) | 0.5 |
| **O2Wa** | 0.725 (2) | 0.225 (2) | 0.193 (5) | 7.0 (5) | 0.5 |
| **Sr1B** | 0.9328 (7) | 0.5672 (7) | 0 | 4.7 (3) | 0.5 |
| **Ti1B** | 0.9054 (10) | 0.4054 (10) | 0 | 1.0 (4) | 0.5 |
| **O1B** | 0.956 (2) | 0.456 (2) | 0 | 1.6 (6) | 0.5 |
| **O1Wb** | 0.840 (3) | 0.340 (3) | 0 | 1.6 (6) | 0.5 |
| **O2Wb** | 0.825 (3) | 0.675 (3) | 0 | 7.0 (5) | 0.5 |
| **C1** | 0.746 (7) | 0.450 (9) | 0.300 (14) | 3.2 (5) | 1.0 |
| **C2** | 0.785 (7) | 0.450 (9) | 0.234 (14) | 3.2 (5) | 1.0 |
| **C3** | 0.806 (7) | 0.499 (9) | 0.203 (15) | 3.2 (5) | 1.0 |
| **C4** | 0.787 (8) | 0.548 (9) | 0.238 (15) | 3.2 (5) | 1.0 |
| **C5** | 0.748 (8) | 0.547 (9) | 0.304 (15) | 3.2 (5) | 1.0 |
| **C6** | 0.728 (7) | 0.498 (9) | 0.335 (14) | 3.2 (5) | 1.0 |
| **H2** | 0.799 (7) | 0.415 (9) | 0.208 (14) | 4.8 (7) | 1.0 |
| **H4** | 0.802 (8) | 0.584 (9) | 0.216 (15) | 4.8 (7) | 1.0 |
| **H6** | 0.699 (7) | 0.498 (9) | 0.383 (14) | 4.8 (7) | 1.0 |
| **C7** | 0.724 (7) | 0.396 (9) | 0.334 (14) | 3.2 (5) | 1.0 |
| **O17** | 0.746 (7) | 0.348 (9) | 0.305 (14) | 3.2 (5) | 1.0 |
| **O27** | 0.683 (7) | 0.395 (8) | 0.392 (13) | 3.2 (5) | 1.0 |
| **C8** | 0.848 (7) | 0.500 (9) | 0.131 (15) | 3.2 (5) | 1.0 |
| **O18** | 0.866 (8) | 0.548 (9) | 0.098 (16) | 3.2 (5) | 1.0 |
| **O28** | 0.868 (7) | 0.453 (9) | 0.099 (15) | 3.2 (5) | 1.0 |
| **C9** | 0.727 (8) | 0.601 (9) | 0.343 (15) | 3.2 (5) | 1.0 |
| **O19** | 0.680 (8) | 0.602 (8) | 0.387 (15) | 3.2 (5) | 1.0 |
| **O29** | 0.757 (8) | 0.647 (9) | 0.334 (16) | 3.2 (5) | 1.0 |
| **O1W** | 0.117 (6) | 0.404 (6) | 0.5 | 12 | 0.82 (6) |
| **O2W** | 0.615 (3) | 0.526 (3) | −0.169 (5) | 12 | 0.82 (5) |
| **O3W** | 0.5 | 0.5 | 0 | 12 | 1.0 |
| **O5W** | 1.059 (4) | 0.955 (4) | 0.294 (3) | 12 | 1.00 (4) |
| **O6W** | 0.4150 (17) | 0.0850 (17) | 0.346 (4) | 12 | 0.92 (3) |
| **O8W** | 0.661 (5) | 0.814 (5) | 0.5 | 12 | 0.88 (6) |
| **O9W** | −0.057 (3) | 0.5 | 0.258 (5) | 12 | 0.59 (3) |
| **O11W** | 0.685 (3) | 0.525 (4) | 0 | 12 | 1.00 (6) |
| **C1ace1** | 0.33 (4) | −0.35 (3) | 0.14 (7) | 12 | 0.560 (11) |
| **O1ace1** | 0.29 (4) | −0.34 (3) | 0.19 (7) | 12 | 0.560 (11) |
| **C2ace1** | 0.35 (4) | −0.40 (3) | 0.11 (8) | 12 | 0.560 (11) |
| **C3ace1** | 0.37 (4) | −0.30 (3) | 0.12 (7) | 12 | 0.560 (11) |
| **H21ace1** | 0.32 (4) | −0.42 (3) | 0.07 (7) | 12 | 0.560 (11) |
| **H22ace1** | 0.39 (4) | −0.40 (4) | 0.08 (8) | 12 | 0.560 (11) |
| **H23ace1** | 0.35 (4) | −0.43 (3) | 0.16 (8) | 12 | 0.560 (11) |
| **H31ace1** | 0.36 (3) | −0.28 (3) | 0.07 (7) | 12 | 0.560 (11) |
| **H32ace1** | 0.37 (3) | −0.27 (4) | 0.17 (7) | 12 | 0.560 (11) |
| **H33ace1** | 0.41 (4) | −0.31 (4) | 0.12 (8) | 12 | 0.560 (11) |

## S.3.2. MUV-10(Ba)

**Table S4.** Crystallographic Information of MUV-10(Ba).

| Name | As-made | Evacuated |
|---|---|---|
| **Identification code** | 2473912 | 2473910 |
| **Empirical formula** | $C_{80}H_{24}Ba_6N_4O_{89}Ti_6$ | $C_{72}H_{24}Ba_6O_{66.33}Ti_6$ |
| **Formula weight** | 3576.47 | 3061.71 |
| **Temperature/K** | 293(2) | 273.15 |
| **Crystal system** | tetragonal | tetragonal |
| **Space group** | $P4_2/mnm$ | P4/mbm |
| **a/Å** | 24.03140(10) | 24.4272(7) |
| **b/Å** | 24.03140(10) | 24.4272(7) |
| **c/Å** | 29.69710(10) | 14.4709(9) |
| **α/°** | 90 | 90 |
| **β/°** | 90 | 90 |
| **γ/°** | 90 | 90 |
| **Volume/Å$^3$** | 17150.32(15) | 8634.6(7) |
| **Z** | 4 | 2 |
| **$\rho_{calc}$g/cm$^3$** | 1.385 | 1.178 |
| **μ/mm$^{-1}$** | 1.811 | 13.250 |
| **F(000)** | 6848.0 | 2909.0 |
| **Crystal size/mm$^3$** | 0.02 × 0.02 × 0.01 | 0.01 × 0.01 × 0.01 |
| **Radiation** | synchrotron (λ = 0.72932) | CuKα (λ = 1.54178) |
| **2Θ range for data collection/°** | 3.478 to 67.828 | 5.116 to 130.18 |
| **Index ranges** | $-36 \le h \le 36$, $-35 \le k \le 35$, $-45 \le l \le 45$ | $-24 \le h \le 21$, $-28 \le k \le 24$, $-16 \le l \le 14$ |
| **Reflections collected** | 318544 | 33502 |
| **Independent reflections** | 15826 [$R_{int}$ = 0.1166, $R_{sigma}$ = 0.0316] | 3970 [$R_{int}$ = 0.0726, $R_{sigma}$ = 0.0452] |
| **Data/restraints/parameters** | 15826/0/501 | 3970/0/233 |
| **Goodness-of-fit on F$^2$** | 1.076 | 1.183 |
| **Final R indexes [I>=2σ (I)]** | $R_1$ = 0.0710, $wR_2$ = 0.1871 | $R_1$ = 0.0616, $wR_2$ = 0.1945 |
| **Final R indexes [all data]** | $R_1$ = 0.0723, $wR_2$ = 0.1879 | $R_1$ = 0.0837, $wR_2$ = 0.2094 |
| **Largest diff. peak/hole / e Å$^{-3}$** | 4.42/-3.35 | 1.10/-0.92 |

## S.3.3. TOLERANCE FACTOR CALCULATIONS

The simplified Goldschmidt tolerance factor ($t$) was used as a comparative geometric descriptor to assess the degree of strain introduced by A-site cation substitution in the MUV-10(M) series. The formula used is:

$$t = \frac{\mathrm{rA} + rX}{\sqrt{2} \cdot (rB + rX)}$$

where $r_A$, $r_B$, and $r_X$ correspond to the ionic radii of the A-site cation ($Ca^{2+}$, $Sr^{2+}$, $Ba^{2+}$), the B-site cation ($Ti^{4+}$), and the bridging anion ($O^{2-}$), respectively.

Ionic radii were taken from Shannon's effective ionic radii,[10] based on the most representative coordination numbers in the MUV-10(M) structure:

| Ion | Oxidation state | Coordination number (CN) | Radius (Å) |
|---|---|---|---|
| $Ca^{2+}$ | 2+ | 7 | 1.06 |
| $Sr^{2+}$ | 2+ | 6 | 1.18 |
| $Ba^{2+}$ | 2+ | 9 | 1.47 |
| $Ti^{4+}$ | 4+ | 6 | 0.605 |
| $O^{2-}$ | 2- | 3 | 1.36 |

Compared to perovskite oxides, where the tolerance factor evaluates the packing of the entire crystal lattice, our simplification focuses on the internal geometry of the tetranuclear $Ti_2M_2$ cluster. In this node, two $Ti^{4+}$ and two $M^{2+}$ cations are bridged by two $\mu_3$-oxo ligands. Thus, the simplified t value here reflects the geometric compatibility between the A-site cation and the $\mu_3$-O–Ti–M bonding environment in the cluster. While this simplified factor does not account for the whole lattice, it provides a meaningful comparative metric for interpreting the observed trends in octahedral distortion and local strain within the inorganic node.

## S.3.4. ANGULAR DISTORTION METRICS ANALYSIS

Bond length deviation (Δd) and angular distortion (Δθ) from the ideal $TiO_6$ geometry were calculated for each crystallographically independent titanium center in the MUV-10(M) structures. The values reported in Figure 3b correspond to the average of the individual values. These calculations were performed using custom Python tools developed in-house (codename: *TopoTemp*).

Bond length distortion (Δd)

The deviation of the metal–ligand bond lengths from their average value and was computed as:

$$\Delta d = \frac{1}{n}\sum_{i=n}^{n} |d_i - \bar{d}|$$

Where:

$d_i$ is the distance between the titanium atom and the i-th coordinating oxygen atom,

$\bar{d}$ is the average Ti–O bond length,

n = 6 (the number of Ti–O bonds in the octahedron).

Angular distortion (Δθ)

The deviation of the O–Ti–O angles from the ideal 90° in a regular octahedron (cis angles only) was computed a:

$$\Delta\theta = \frac{1}{m}\sum_{j=1}^{m} |\theta_j - 90^o|$$

Where:

$\theta_j$ are the O–Ti–O angles involving paris of adjacent ligands, angles larger than 120° are excluded to consider only cis angles.

$m$ = 12 is the number of such angles.

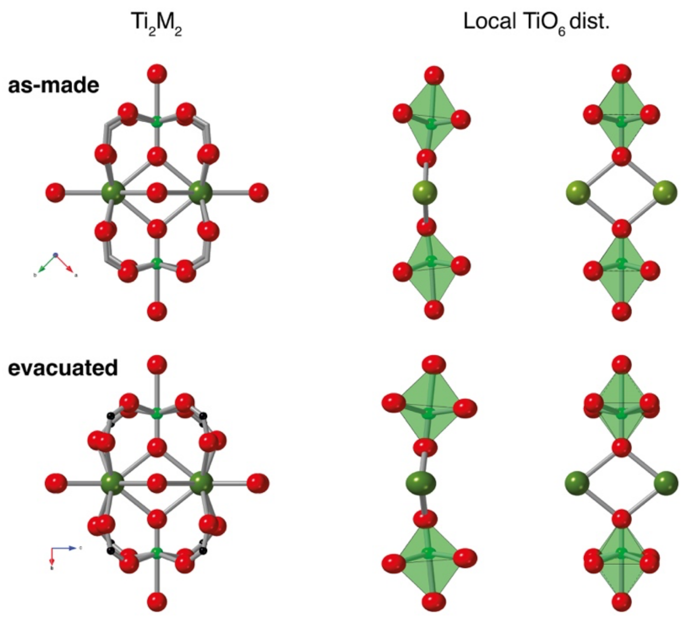


**Figure S11**. Simplified representation of the $Ti_2Ba_2$ cluster core (left) and local $TiO_6$ coordination environment (right) in MUV-10(Ba) as-synthesized (top) and after evacuation at 100 °C (bottom). Although minor variations in atomic positions are observed upon solvent removal, the distorted geometry around the Ti(IV) centers and the symmetry-lowering features are retained, confirming that the cluster strain originates primarily from the size of the A-site cation.

## S.4. PHOTOCHARACTERIZATION

### S.4.1. UV-VIS DIFFUSE REFLECTANCE SPECTROSCOPY

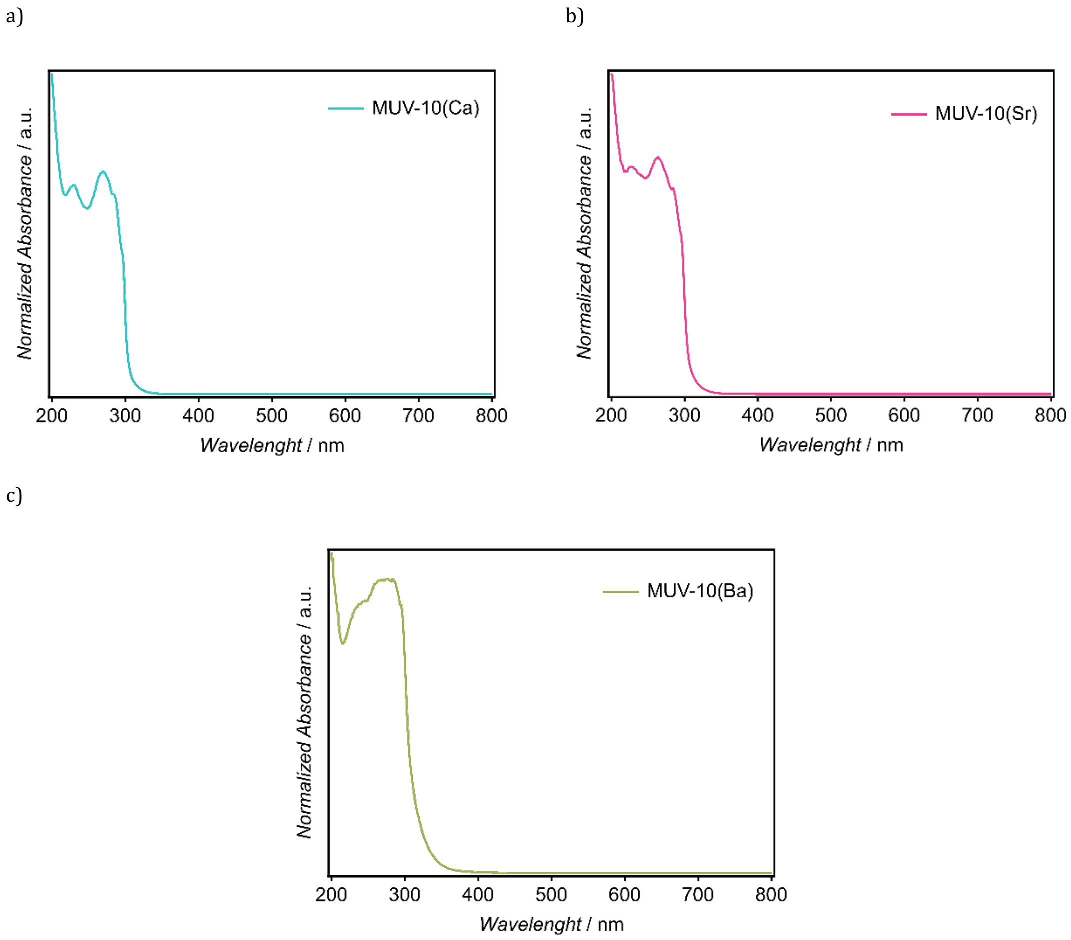


**Figure S12.** UV-VIS spectrum of all MUV-10(M) material family, As Made: a) MUV-10(Ca), b) MUV-10(Sr), c) MUV-10(Ba).

## S.4.2. EXPERIMENTAL OPTICAL BAND-GAP CALCULATION

The optical band gap was determined from diffuse reflectance data using the Tauc Method,[11] based on the assumption that the energy-dependent absorption coefficient α can be expressed by the following equation (1):

$$(\alpha \cdot hv)^{1/\gamma} = B(hv\text{-}E_g) \qquad \text{Equation (1)}$$

where $h$ is the Planck constant, $v$ is the photon's frequency, $E_g$ is the band gap energy, and $B$ is a constant.

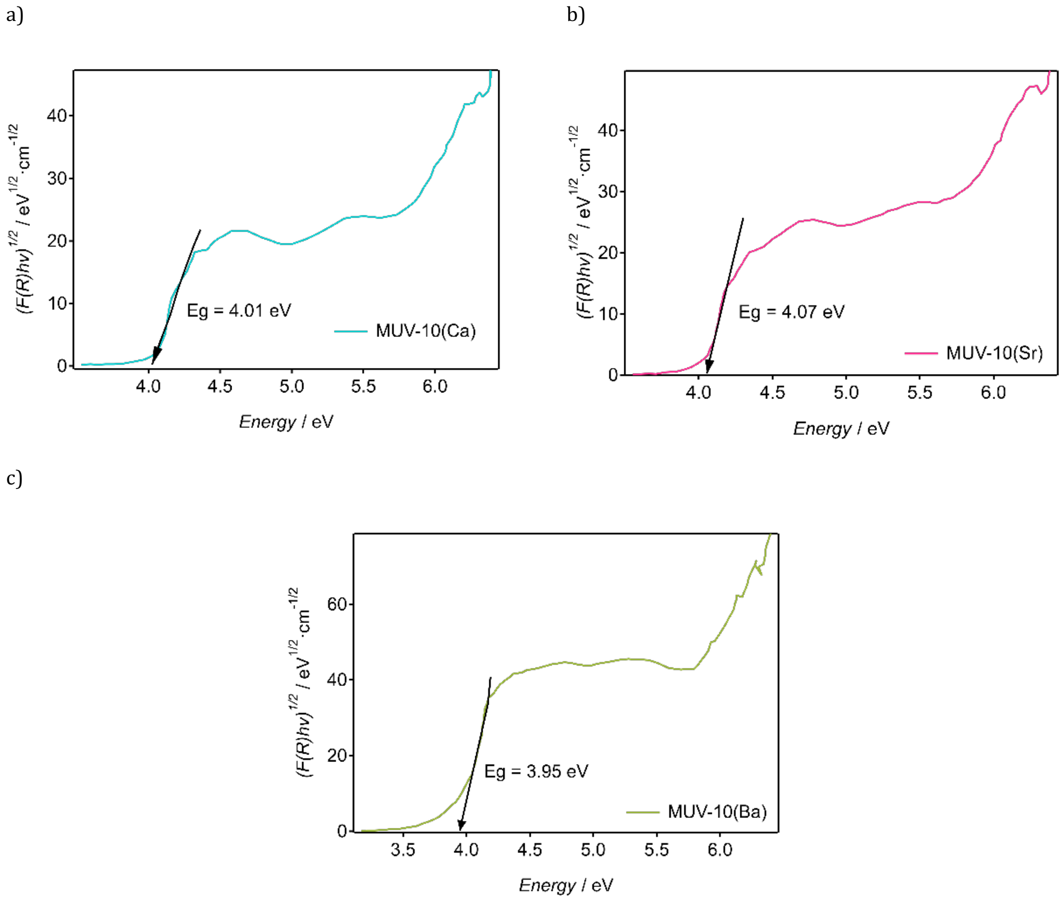


**Figure S13.** Tauc plot [F(R) x hv]$^2$ vs (hv)$^2$ for optical band gap energy (Eg) for materials a) MUV-10(Ca), b) MUV-10(Sr), c) MUV-10(Ba); where the black arrows correspond to the regression fitting of the linear part of the plot.

## S.4.3. ULTRAVIOLET PHOTOELECTRON SPECTROSCOPY SPECTRA.

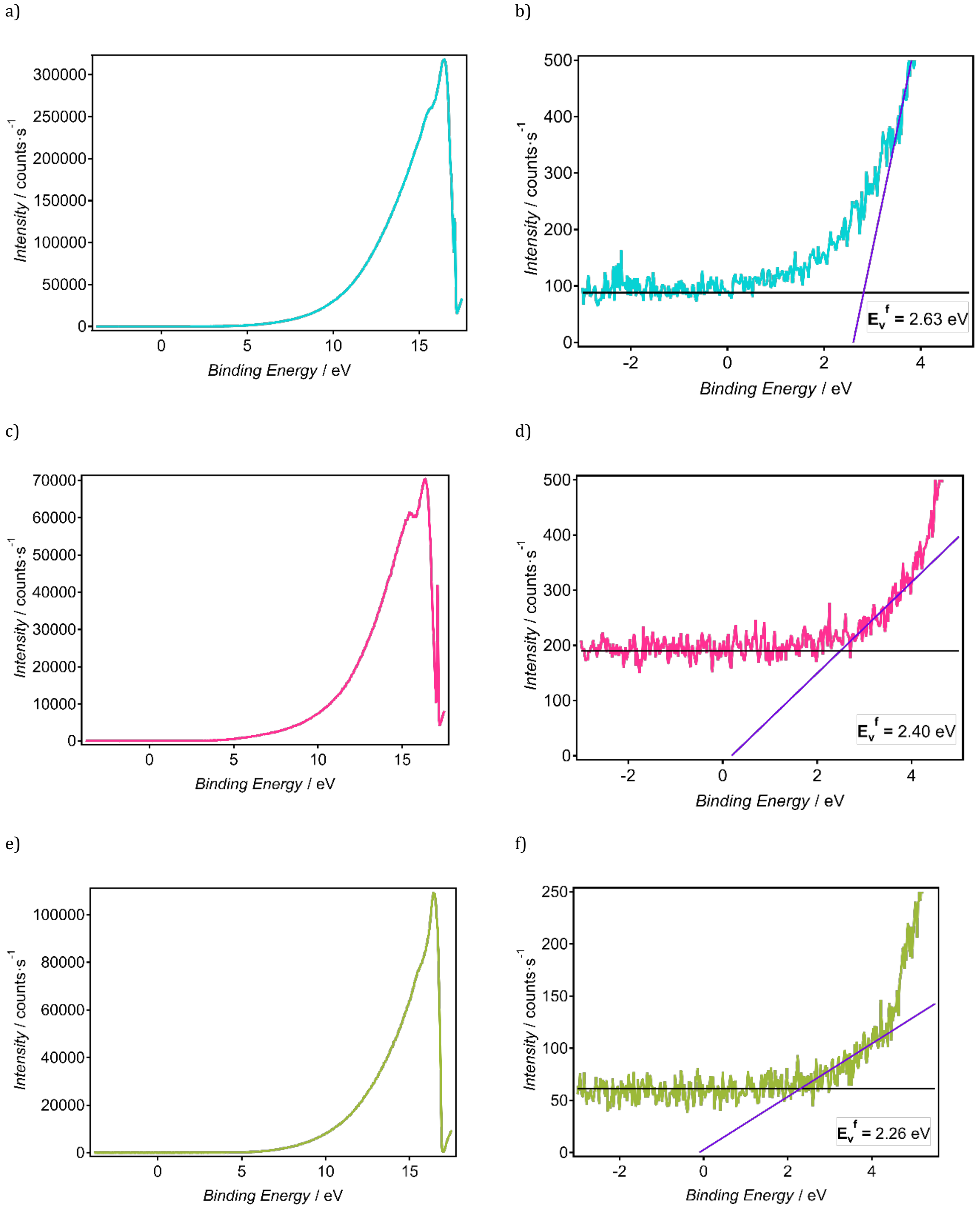


**Figure S14.** UPS of MUV-10(M) materials, a) and b) M = Ca, c) and d) M= Sr, e) and f) M = Ba.

## S.5. PHOTODEPOSITION OF RUTHENIUM

### S.5.1. PHOTODEPOSITION METHOD

$RuO_x$ NPs were deposited in MUV-10(M) (M = Ca, Sr or Ba) using the so-called photodeposition method.[5] Briefly, MUV-10 solid (50 mg) was placed into a quartz tube containing a mixture of Milli-Q water (8 mL) and methanol (13 mL). Then, the corresponding amount of $KRuO_4$ (1wt% Ru) dissolved in water (1 mL) was added to this tube and the suspension purged with Ar for 30 min. Subsequently, the system was irradiated with an UV-Vis lamp for 4 h. The resulting solid was recovered by filtration, washed several times with Milli-Q water and placed in an oven at 100 ºC overnight and, then, was submitted to vacuum at 150 ºC for 24 h to remove the solvents.

## S.5.2. CHARACTERIZATION AFTER DEPOSITION

To check the stability of these materials after the photodeposition process, a characterization of the materials is performed by SEM, PXRD, EDX and ICP-MS.

a)

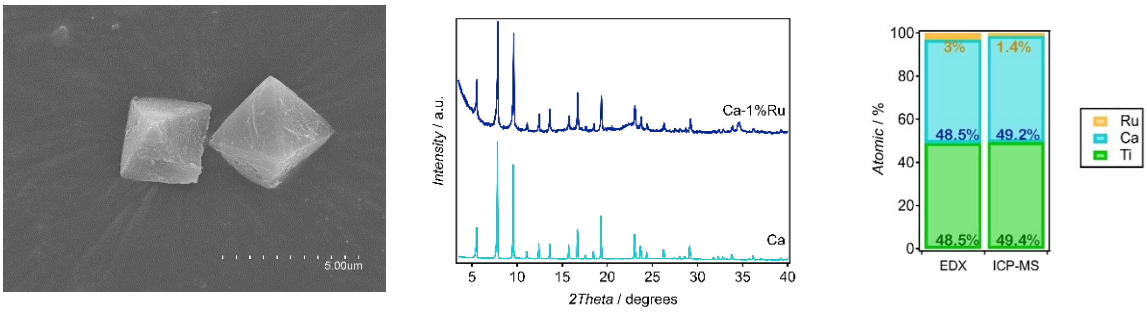


b)

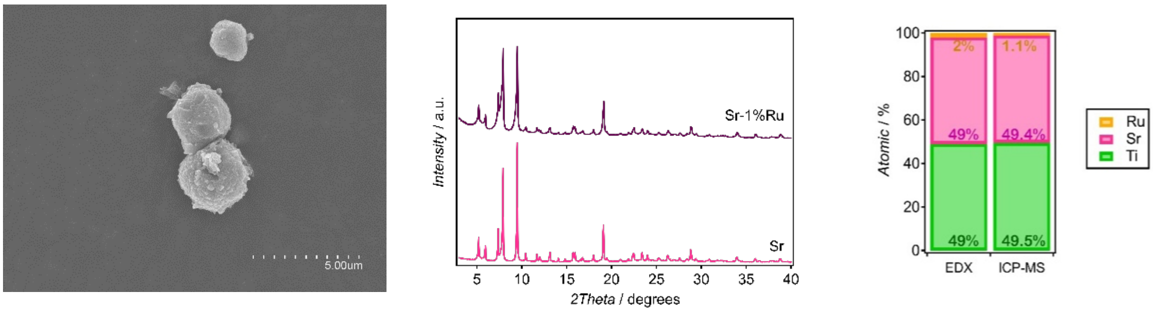


c)

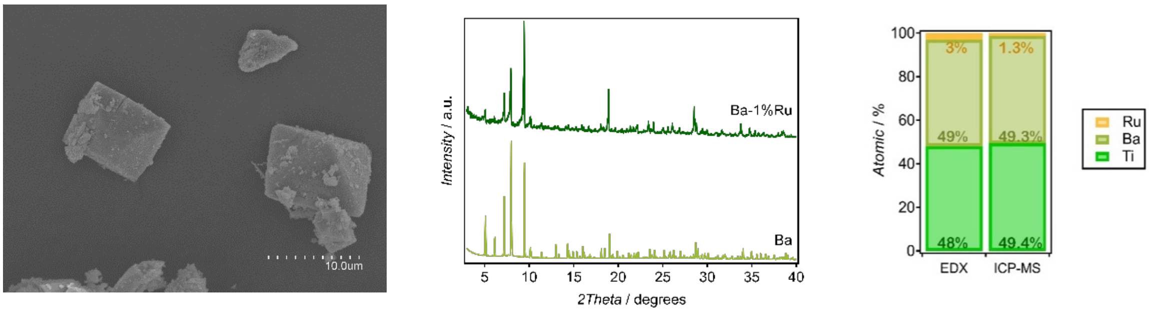


**Figure S15.** Characterization of the MUV-10(M) family of materials after $RuO_x$ deposition a) Ca, b) Sr, c) Ba; by SEM, PXRD, EDX and ICP-MS.

# S.6. PHOTOCHARACTERIZACION AFTER Ru PHOTODEPOSITION

## S.6.1. X-RAY PHOTOELECTRON SPECTROSCOPY SPECTRA

$RuO_x$@MUV-10(Ca), $RuO_x$@MUV-10(Sr) and $RuO_x$@MUV-10(Ba) were characterized by XPS. The most salient differences compared to pristine MUVs is the presence of new XPS signals associated with ruthenium species. Regardless of partially overlappin of Ru 3 d XPS region with C 1s spectrum, the small band at 282.1 eV can be associated with Ru $3d_{5/2}$. XPS Ru 3p region also partially overlaps with Ti 2p but two bands at 484.5 and 461.0 can be deconvoluted from XPS spectra associated with Ru $3p_{3/2}$ and $3p_{1/2}$ of oxidized RuOx species, respectively.

$RuO_x$@MUV-10(Ca)

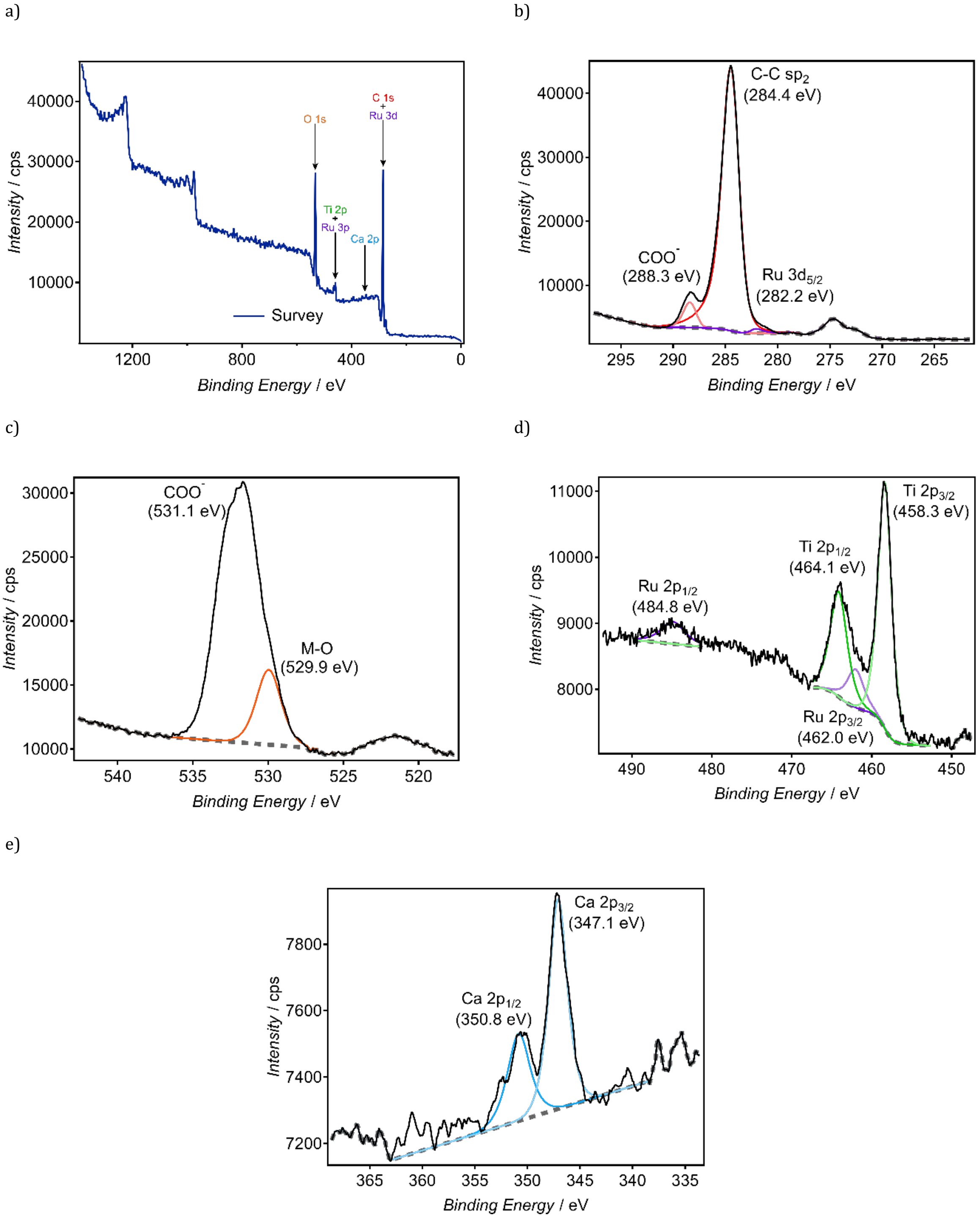


**Figure S16.** XPS a) Survey, b) C 1s + Ru 3d, c) O 1s, d) Ru 3p + Ti 2p, e) Ca 2p of $RuO_x$@MUV-10(Ca).

RuO$_x$@MUV-10(Sr)

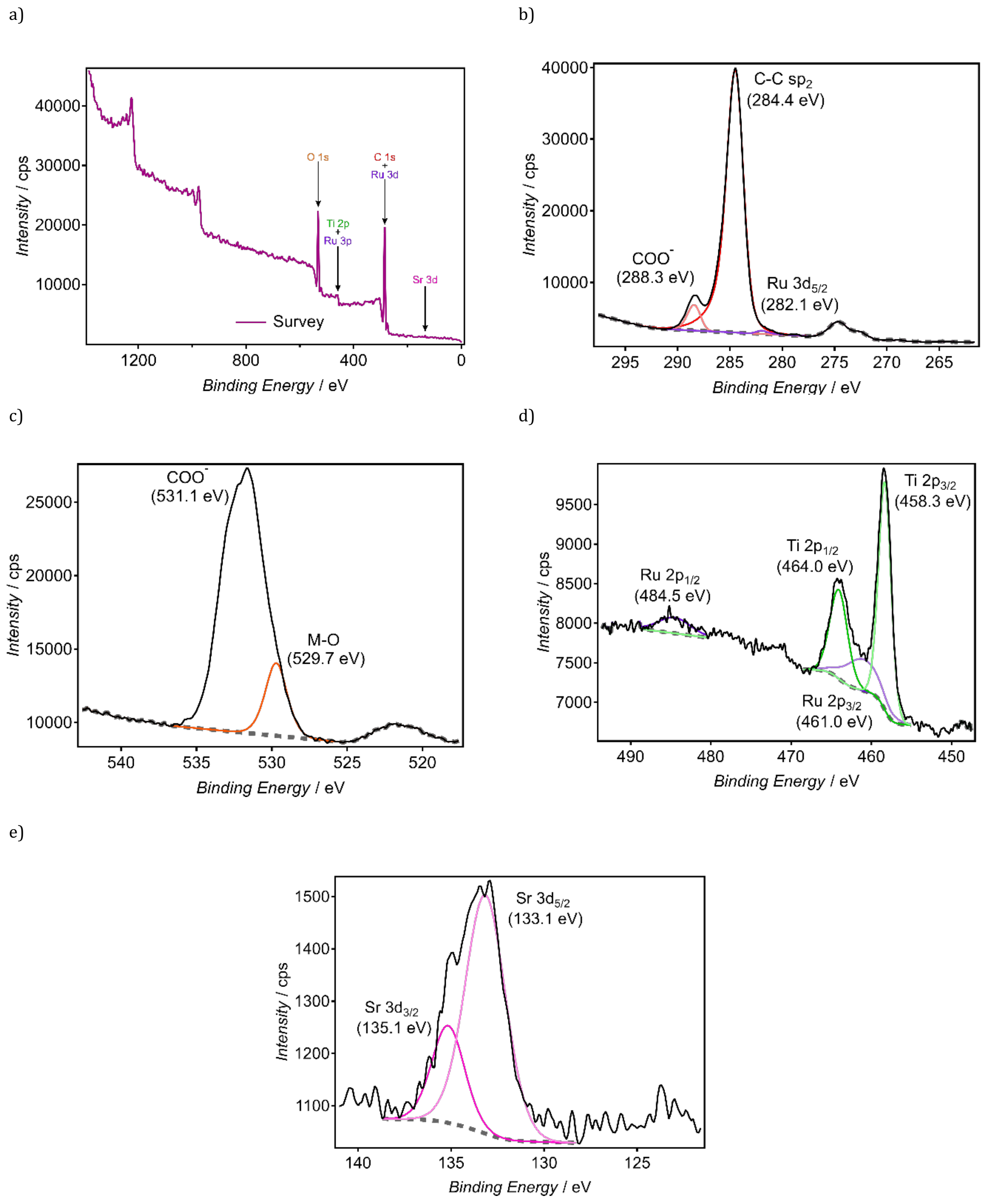


**Figure S17.** XPS a) Survey, b) C 1s + Ru 3d + Sr 3p, c) O 1s, d) Ru 3p + Ti 2p, e) Sr 3d of RuO$_x$@MUV-10(Sr)

RuO$_x$@MUV-10(Ba)

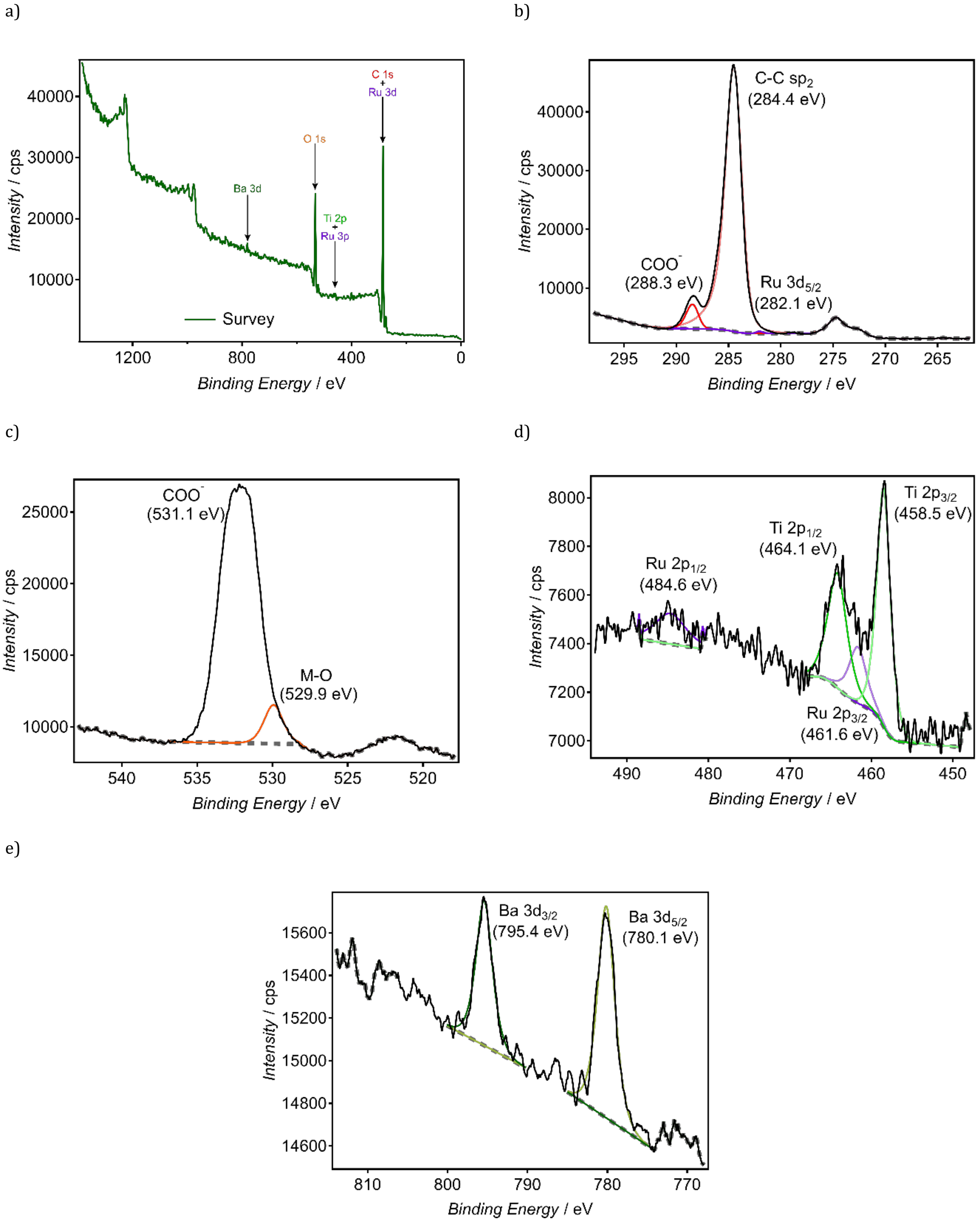


**Figure S18.** XPS a) Survey, b) C 1s + Ru 3d, c) O 1s, d) Ru 3p + Ti 2p, e) Ba 3d of RuO$_x$@MUV-10(Ba).

Comparison of RuOx@MUV-10(M) (M = Ca, Sr or Ba) materials.

a)

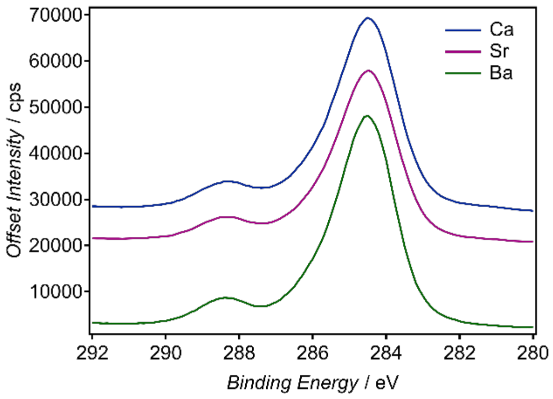


b)

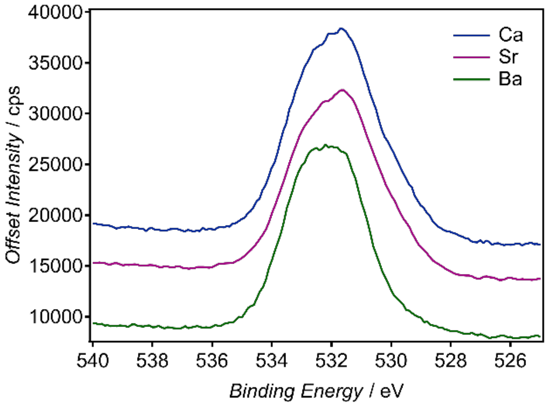


c)

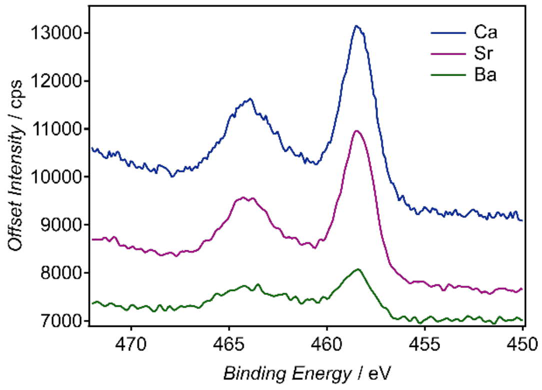


**Figure S19.** Comparison of XPS a) C 1s, b) O 1s, and c) Ti 2p, zones of $RuO_x$@MUV-10(M) materials (M = Ca, Sr or Ba).

## S.6.2. TRANSMISION ELECTRONIC MICROSCOPY

$RuO_x$@MUV-10(Ca)

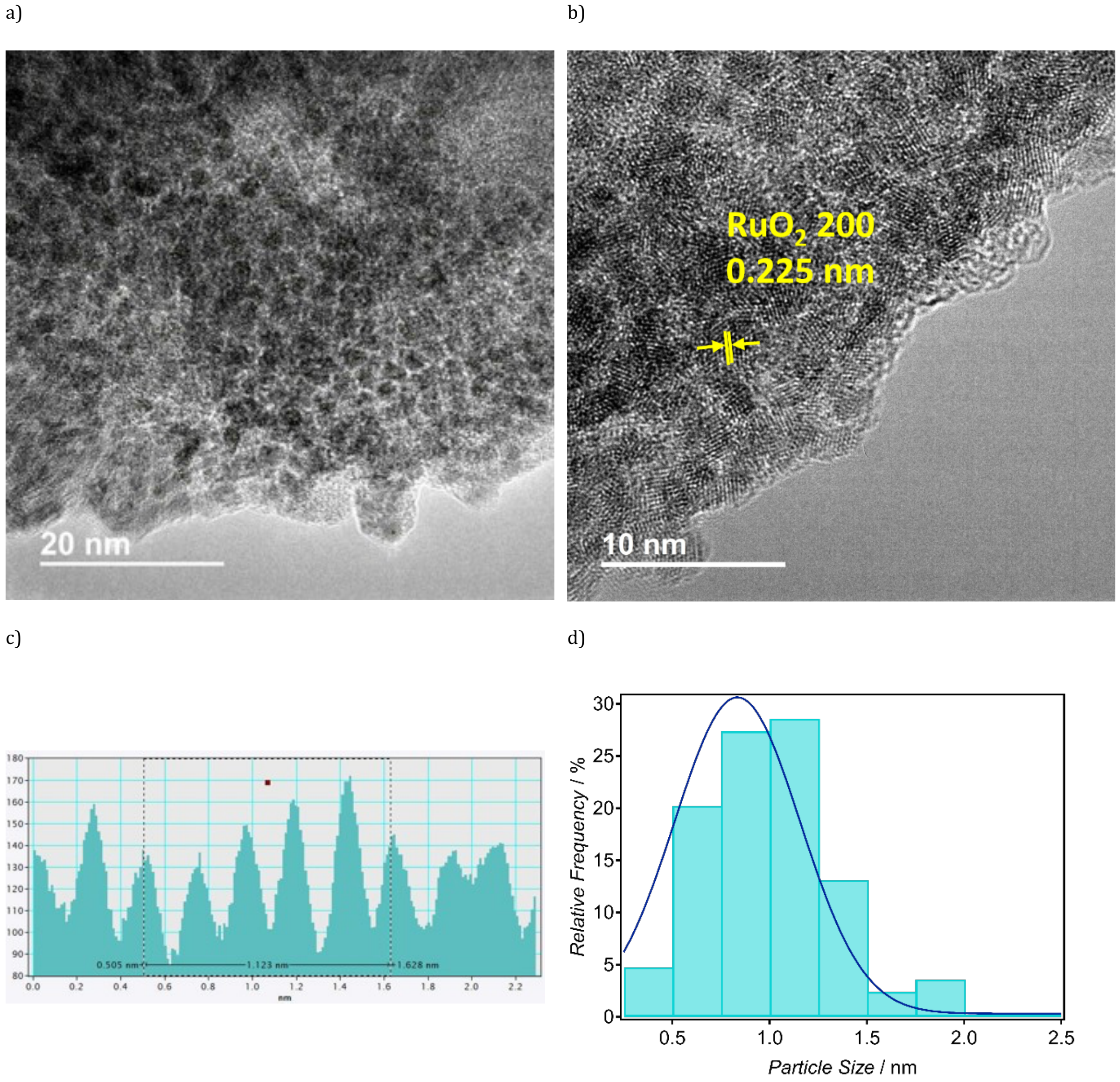


**Figure S20.** a and b) TEM image of $RuO_x$@MUV-10(Ca), with c) $RuO_x$ NPs interplane distance measurement, and d) size distribution.

RuO$_x$@MUV-10(Sr)

a)

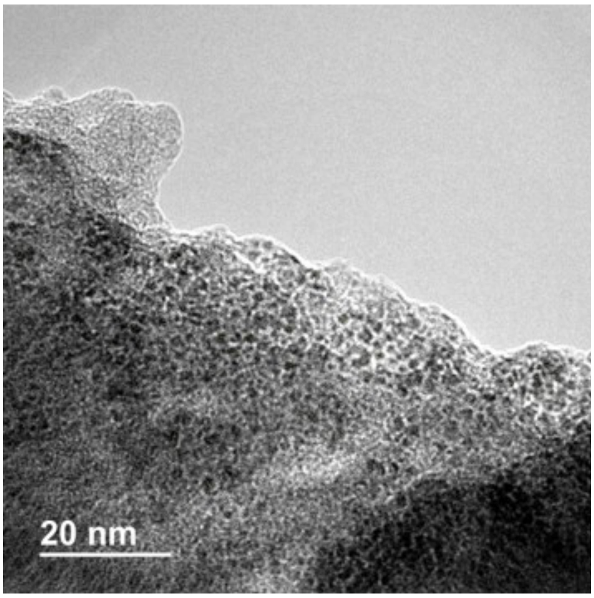


b)

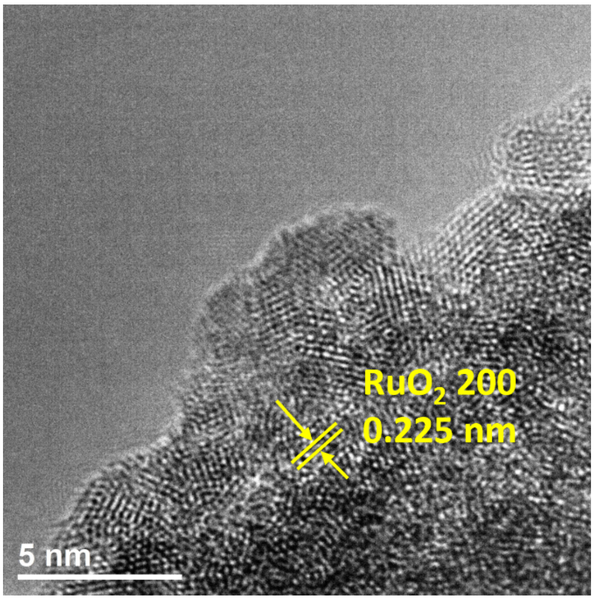


c)

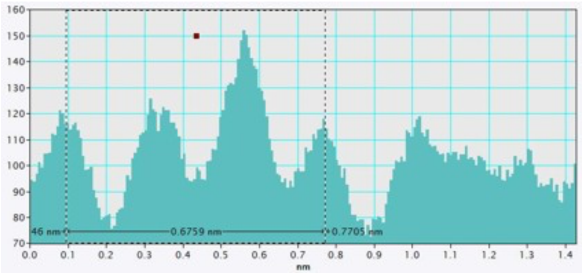


d)

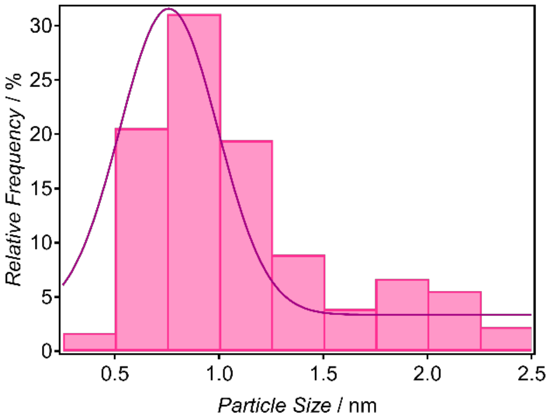


**Figure S21.** a and b) TEM image of RuO$_x$@MUV-10(Sr), with c) RuO$_x$ NPs interplane distance measurement, and d) size distribution.

RuO$_x$@MUV-10(Ba)

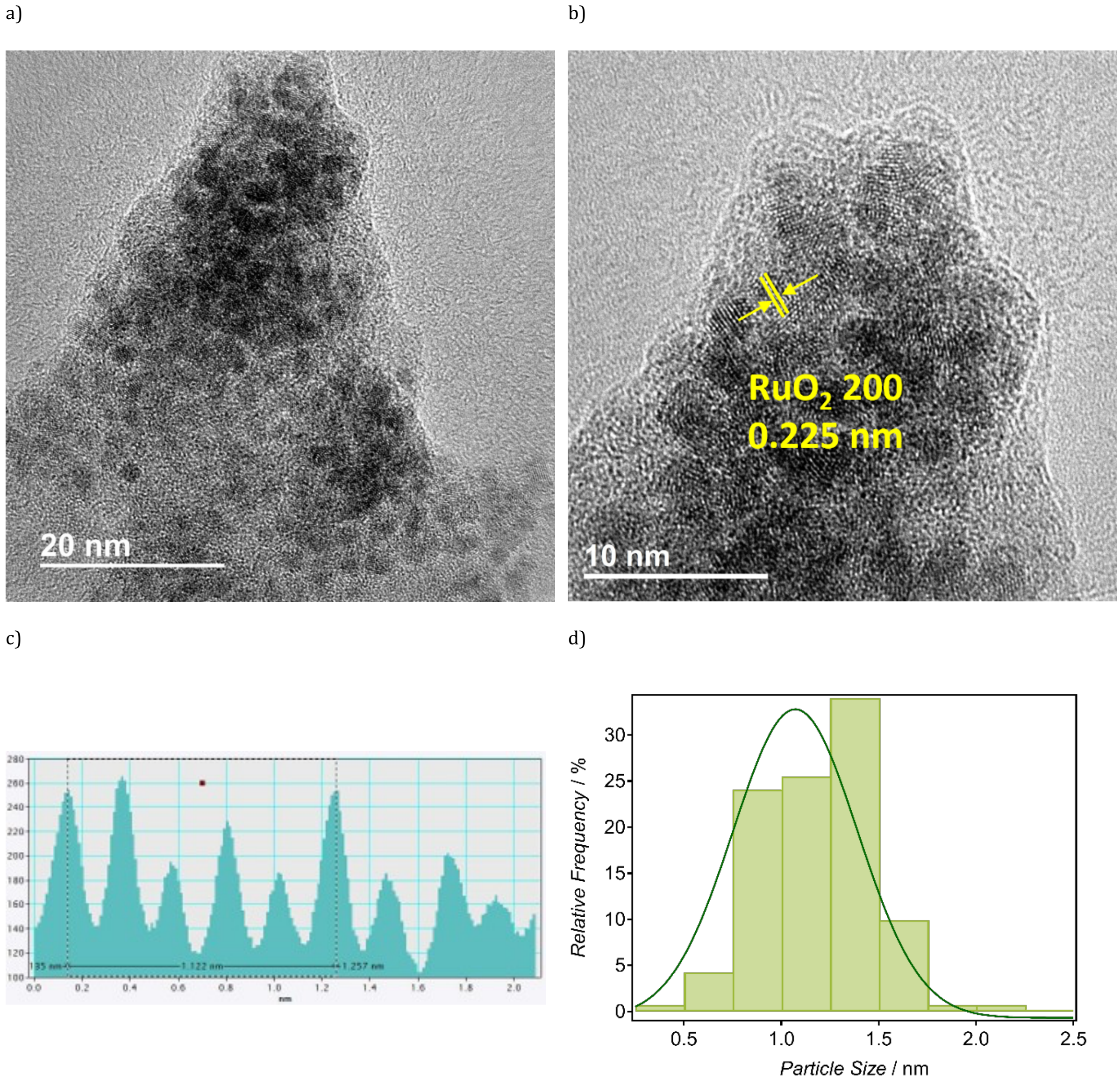


**Figure S22.** a and b) TEM image of RuO$_x$@MUV-10(Ba), with c) RuO$_x$ NPs interplane distance measurement, and d) size distribution.

## S.6.3. UV-VIS DIFFUSE REFLECTANCE SPECTROSCOPY

To study the interaction of these materials with light, the UV-Vis Diffuse Reflectance Spectroscopy of the $RuO_x$@MUV-10 materials is measured.

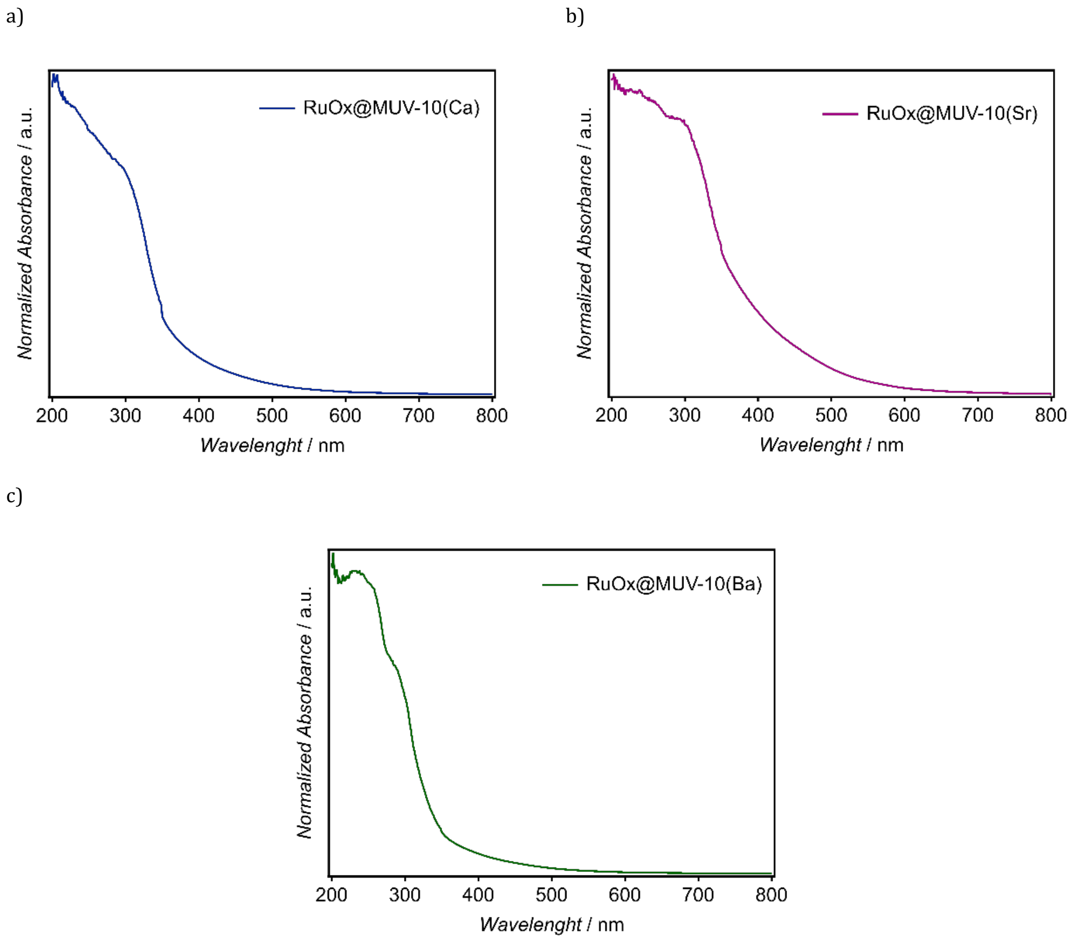


**Figure S23.** UV-VIS spectrum of all MUV-10(M) material family, after $RuO_x$ photodeposition: a) MUV-10(Ca), b) MUV-10(Sr), c) MUV-10(Ba).

## S.6.4. PHOTOCURRENT EXPERIMENTS

Photocurrent measurements were carried out using a Gamry Instruments potentiostat (model Interface 5000E). A three-electrode cell was employed in a home-made quartz cell. A platinum wire was used as counter electrode and a saturated Ag/AgCl electrode as the reference. The working electrode (WE) consisted of a conductive carbon Toray paper (2.0 x 1.0 $cm^2$) containing the MOF-based material. To prepare the WE, a disperson of MOF (20 mg), terpineol (0.2 mL) and acetone (0.5 mL) was prepared by magnetic stirring and, then incubated at 90 °C overnight. This mixture was cool down, spread (25 µL) onto the carbon paper and thermally treated at 150 °C for 2 h. EIS measurements were performed frequencies ranging from 0.1 Hz to 100 kHz at + 0.2 V. Prior to these measurements, the electrolyte solutions were purged with argon for 10 min. The obtained data was visualized in a Nyquist plot and fitted to a Randles equivalent circuit consisting of a solution resistance, a double layer capacitor and a charge transfer resistance (Rct). The photocurrent generated by the electrodes was measured by chopped linear sweep voltammetry (LSV) using a $LiClO_4$ acetonitrile solution (0.1 M). The applied potential was +0.2 V.

a)

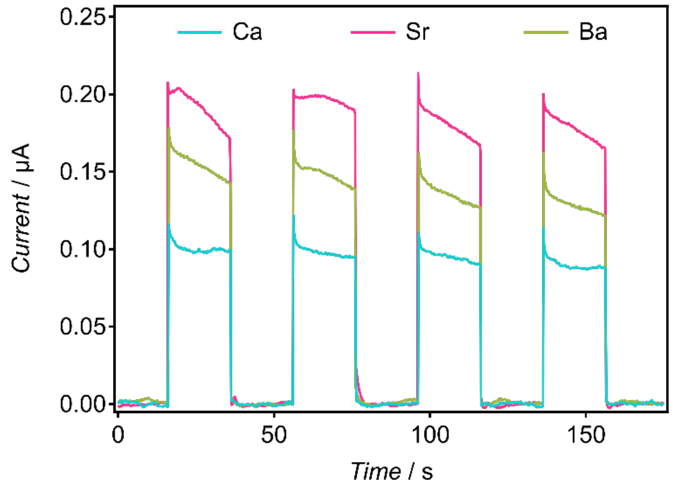


**Figure S24.** Photocurrent measurements of $RuO_x$@MUV-10(M) (M = Ca, Sr or Ba) materials.

## S.6.5. NYQUIST PLOT

a)

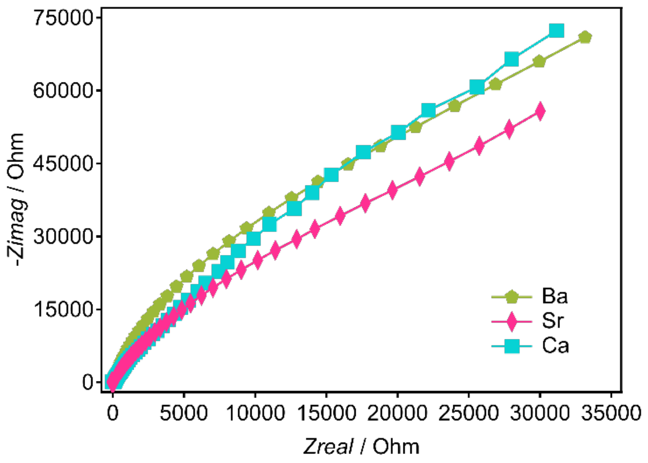


Figure S25. Nyquist Plot of $RuO_x$@MUV-10(M) (M = Ca, Sr, or Ba) materials after photocatalytic Sabatier reaction.

## S.7. COMPUTATIONAL METHODS

### S.7.1. ELECTRONIC STRUCTURE OF MUV-10(X)

From the experimental unit cell of MUV-10, cluster models were made to study the effect of alkaline earth cations on the electronic structure of MUV-10(M) derivatives. Single-point calculations were performed using the screened hybrid functional HSE06,[12] as implemented in the Vienna Ab Initio Simulation Package (VASP).[13,14] The orbital visualizations were generated using the VESTA (Visualization for Electronic and Structural Analysis) software, version 3.4.0.[15]

a) b) c)

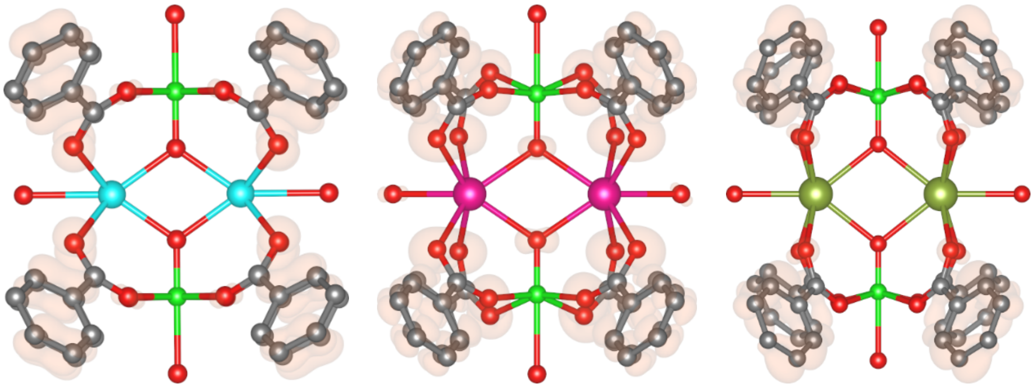

**Figure S26.** Orbitals contribution to the HOCO (orange) of MUV-10(M), M = a) Ca b) Ba and c) Sr.

**Table S5**. Calculated contribution of titanium and the trimesate ligand to the density of states at the conduction band (CB) of the MUV-10(M) series.

| MUV-10(M) | Contribution to CB (%) | |
|---|---|---|
| | Ti | Ligand |
| Ca | 55 | 45 |
| Sr | 70 | 30 |
| Ba | 67 | 33 |

## S.8. ELECTRON PARAMAGNETIC RESONANCE (EPR)

EPR spectra were recorded using 20 mg of solid material suspended in freshly distilled, deoxygenated acetonitrile (MeCN). Prior to measurement, the samples were degassed by three freeze–pump–thaw cycles and sealed under vacuum in quartz tubes using a flame. The sealed samples were then irradiated for 12 hours with a Kessil PR-160L lamp (370 nm, 100% LED intensity) at a distance of 0.5–1 cm from the tube surface.

EPR measurements were carried out using a Bruker ELEXYS E580 spectrometer operating in X-band (~9.3 GHz) at 77 K. To determine the g-factor values, the resonance field was directly measured and used in the following equation:

$$g = h\upsilon/(\mu_B B)$$

where $\upsilon = 9.47 \cdot 10^9$ Hz is the microwave frequency, $h$ is Planck's constant, $\mu_B$ is the Bohr magneton, and B is the magnetic field at resonance.

The following instrumental parameters were used for each EPR measurement: microwave power = 20 mW, modulation amplitude = 1 G. The specific resonance frequencies for each sample were as follows: MUV-10(Ba) = 9.473162 GHz; MUV-10(Ba) irradiated = 9.472541 GHz; MUV-10(Sr) = 9.472945 GHz; MUV-10(Sr) irradiated = 9.483300 GHz. All spectra were recorded at 77 K in quartz tubes. EPR spectrum of RuOx@MUV-10(Sr) was recorded on a Bruker EMX-12 instrument at 100 K operating in X band at 9.433 GHz, modulation amplitude of 1 G and modulation frequency of 100 kHz. Before measuring the EPR spectrum of this solid, a background signal was recorded using the EPR quartz tube. Then, RuOx@MUV-10(Sr) (10 mg) was placed in an EPR quartz tube (4 mm diameter) and purged with argon to remove oxygen. EPR measurements were performed under dark conditions.

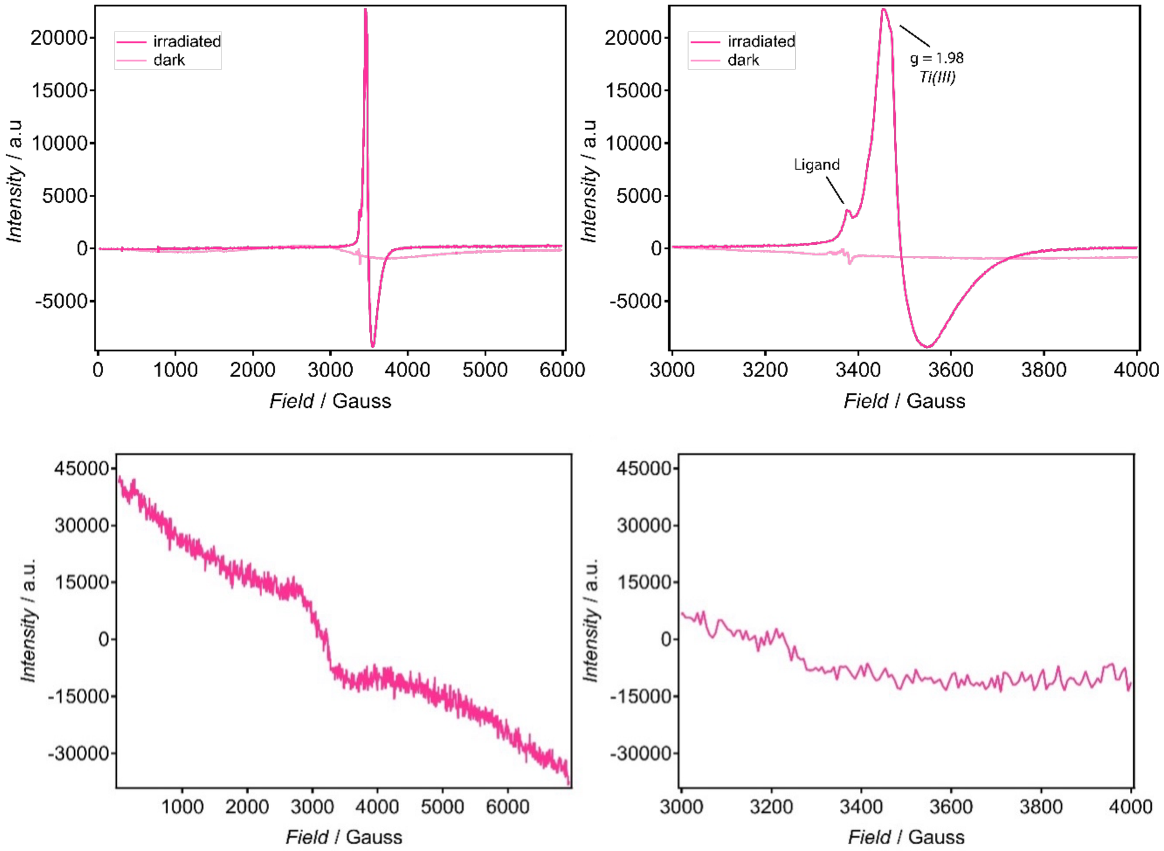


**Figure S27.** top) EPR spectra of MUV-10(Sr) before (light line) and after (dark line) irradiation with UVA light. Bottom) EPR spectrum of $RuO_x$@MUV-10(Sr) recorded under dark conditions at comparable regions, showing no detectable $Ti^{3+}$ signal. These results confirm that photogenerated $Ti^{3+}$ species originate from framework irradiation, not from the RuOx photodeposition process.

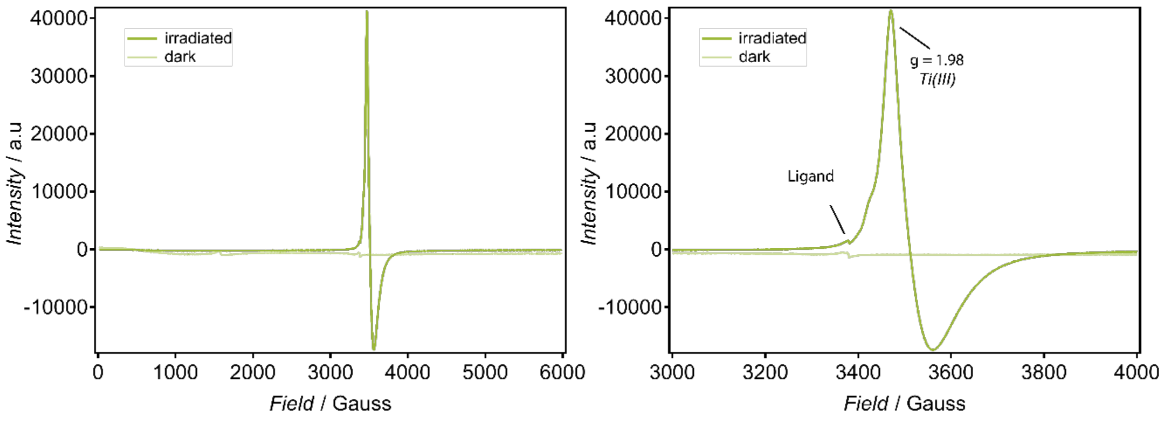


**Figure S28.** EPR spectra of MUV-10(Ba) before (dark line) and after (light line) irradiation with UVA light.

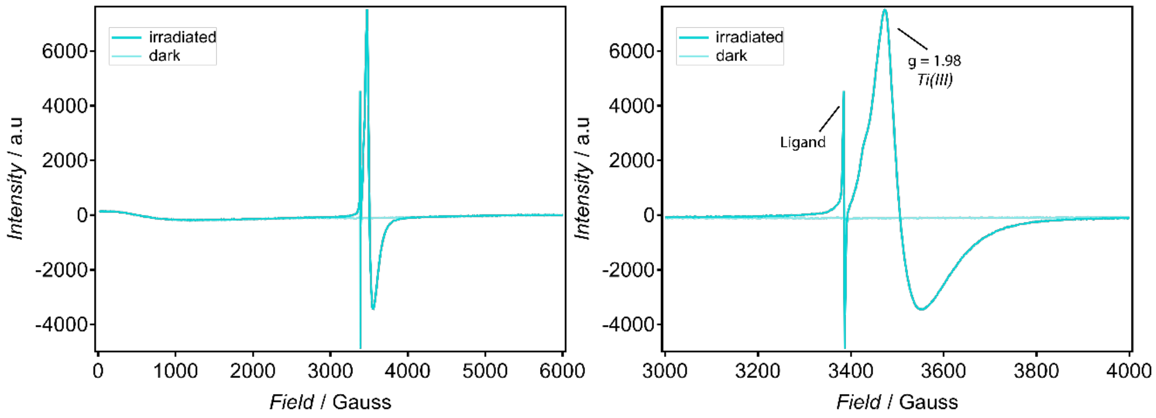


**Figure S29.** EPR spectra of MUV-10(Ca) before (dark line) and after (light line) irradiation with UVA light.

## S.9. PHOTOCATALYSIS EXPERIMENTS

### S.9.1.1 METHODOLOGICAL DETAILS

Photocatalytic $CO_2$ hydrogenation experiments were carried out using a quartz reactor (50 mL) placed in an aluminium heating block equipped with a thermocouple. The solid photocatalyst (15 mg) was placed at the bottom of the reactor. The system was purged with $H_2$ for 15 min and, then, pressurized to 1.2 bar before $CO_2$ (0.3 bar) was added. Simulated sunlight irradiations were performed using a Hg lamp (150 W, Hamamatsu ref. L8253; Hamamatsu spotlight source L9566-04 and light guide A10014-50–0110) equipped with an AM 1.5G type filter (Lasing ref. 81094). In another experiments, commercially available bandpass filters at 400 and 600 nm (Thorlabs, ref FBH400-10 and FBH600-10) were used to estimate the apparent quantum yields at these wavelengths.

### S.9.1.2 PRODUCT ANALYSIS

Photocatalytic product analysis was performed using an Agilent 490 MicroGC equipped with a thermal conductivity detector (TCD). Quantification was done using calibration plots with certified gas mixtures of $CO_2$, $H_2$, $CH_4$, and $C_2H_6$ (Linde Gas España, S.A.U.) in the range of 0.01 to 100 vol%. A Molsieve 5A column was used to quantify $H_2$, $O_2$, $N_2$, and CO, while a PoraPlot Q column was used for $CO_2$, $CH_4$, and hydrocarbons ($C_2$–$C_4$). $CH_4$ was the main product, accompanied by trace amounts of $C_2H_6$ (< 0.7%). No other gaseous species (e.g., CO, $C_2H_4$, $C_3H_8$, $C_3H_6$, $C_4H_{10}$) were detected.

To investigate the possible formation of liquid-phase products, the reaction was stopped after 22 h, the reactor cooled to room temperature, and the solid catalyst extracted with 2 mL of Milli-Q water. The resulting solution was filtered (nylon membrane, 0.45 µm) and analyzed by GC–FID for methanol and ethanol, and by HPLC with diode-array detection for formic acid (HCOOH) and acetic acid ($CH_3COOH$). No liquid-phase products were detected in any of the experiments.

Carbon mass balances were calculated based on inlet and outlet concentrations of carbon-containing species and were consistently above 98%.

## S.9.2. PHOTOCATALYTIC SABATIER REACTION RESULTS

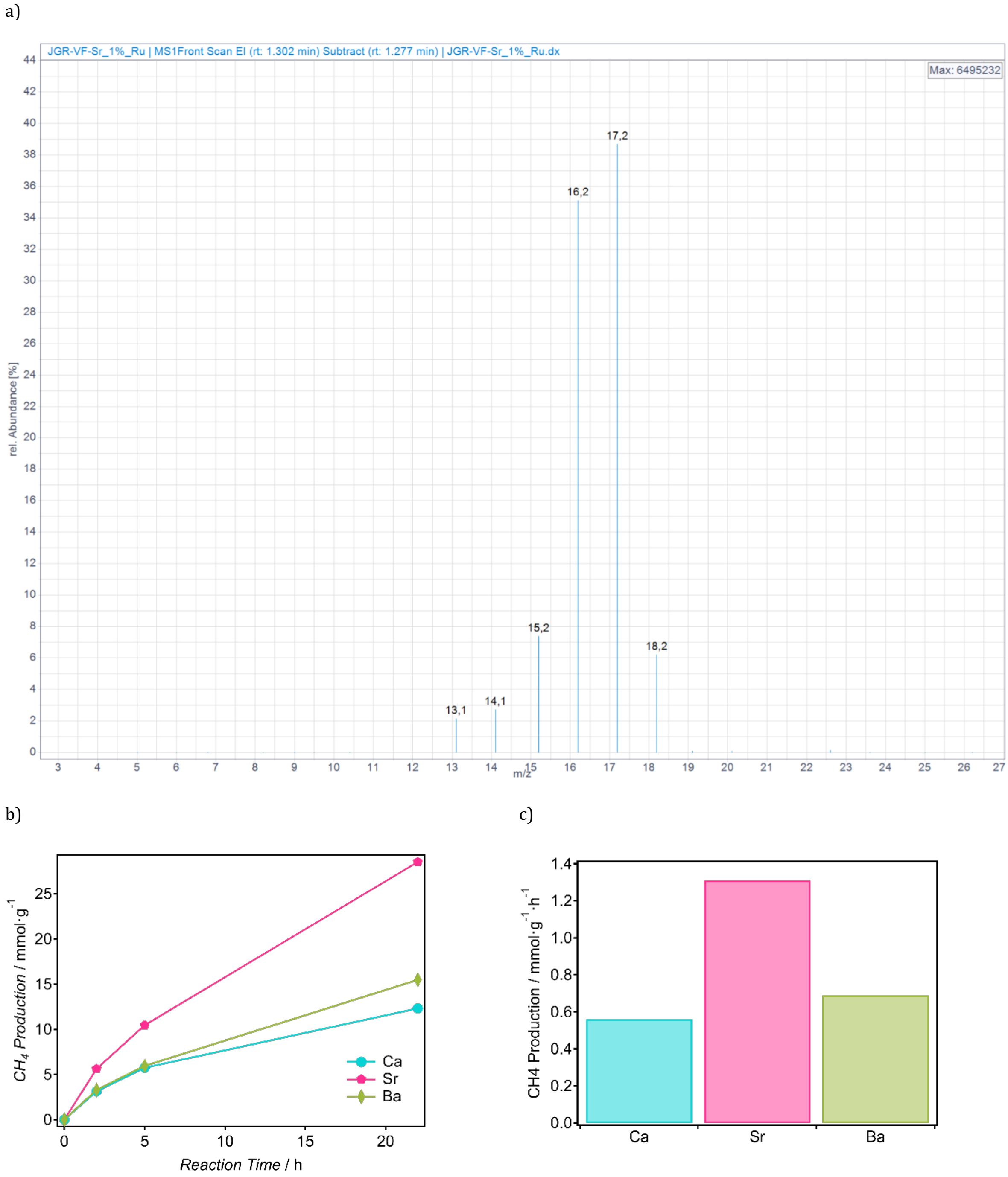


**Figure S30.** Results of Sabatier's Reaction: a) GC-MS result after photocatalytic $^{13}CO_2$ reduction using $RuO_x$@MUV-10(Sr) b) Methane Evolution measured at different times, and c) Yield rate obtained with each material in the Sabatier's reaction.

## S.9.3. CHARACTERIZATION AFTER PHOTOCATALYTIC EXPERIMENTS

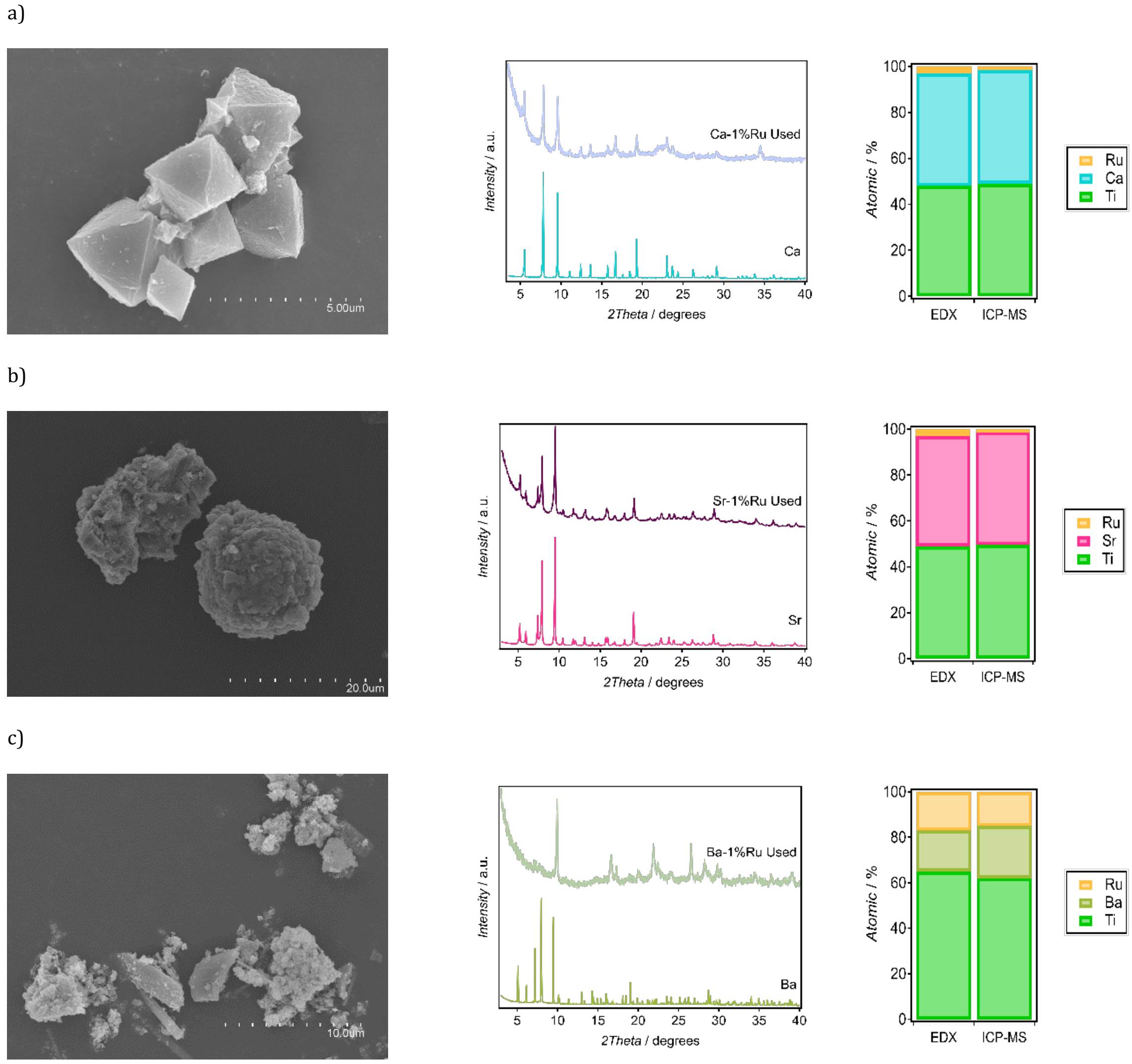


**Figure S31.** Characterization (SEM, PXRD, EDX and ICP-MS) of $RuO_x$@MUV-10(M) family a) Ca, b) Sr, c) Ba, after photocatalytic Sabatier reaction.

**Table S6.** BET surface area of MUV-10(M) materials: as-made, after $RuO_x$ photodeposition and post-catalysis. (Units: $m^2 \cdot g^{-1}$).

| MOF | As Made | After RuOx photodeposition | Post-Catalysis |
|---|---|---|---|
| MUV-10(Ca) | 1009.2 ± 1.3 | 899.0 ± 2.2 | 820.5 ± 1.5 |
| MUV-10(Sr) | 1095.8 ± 1.3 | 971.1 ± 1.0 | 824.1 ± 0.9 |
| MUV-10(Ba) | 1196.7 ± 1.8 | 606.6 ± 1.5 | 83.6 ± 0.3 |

## S.9.4. REUSES CYCLES

To assess the stability and reusability of the $RuO_x$@MUV-10(Sr) catalyst under photocatalytic $CO_2$ methanation conditions, we performed four consecutive catalytic cycles using the same material. After each cycle, no regeneration or additional treatment was applied. The only operation between runs consisted of purging the reactor with fresh reactants. Specifically, after cooling the system to room temperature, the reactor was purged with $H_2$ for 10 min, pressurized to 1.2 bar, and then filled with $CO_2$ to a total pressure of 1.5 bar. This protocol was repeated identically before each catalytic run. No loss in activity or selectivity was observed across the cycles, confirming the absence of catalyst poisoning or degradation during operation

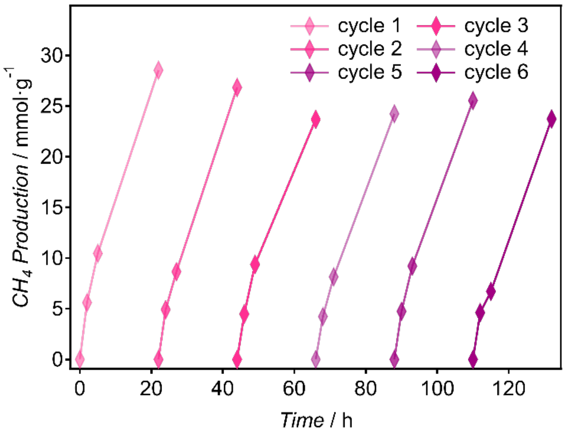


**Figure S32.** Results of different cycles of the Sabatier's reaction with $RuO_x$@MUV-10(Sr).

To evaluate whether repeated use induces changes in surface area or particle morphology, the recovered $RuO_x$@MUV-10(Sr) catalyst was characterized by PXRD, $N_2$ physisorption and SEM analysis after four full photocatalytic cycles. As shown in Figure S33, the photocatalyst remain essentially unchanged compared to the starting material.

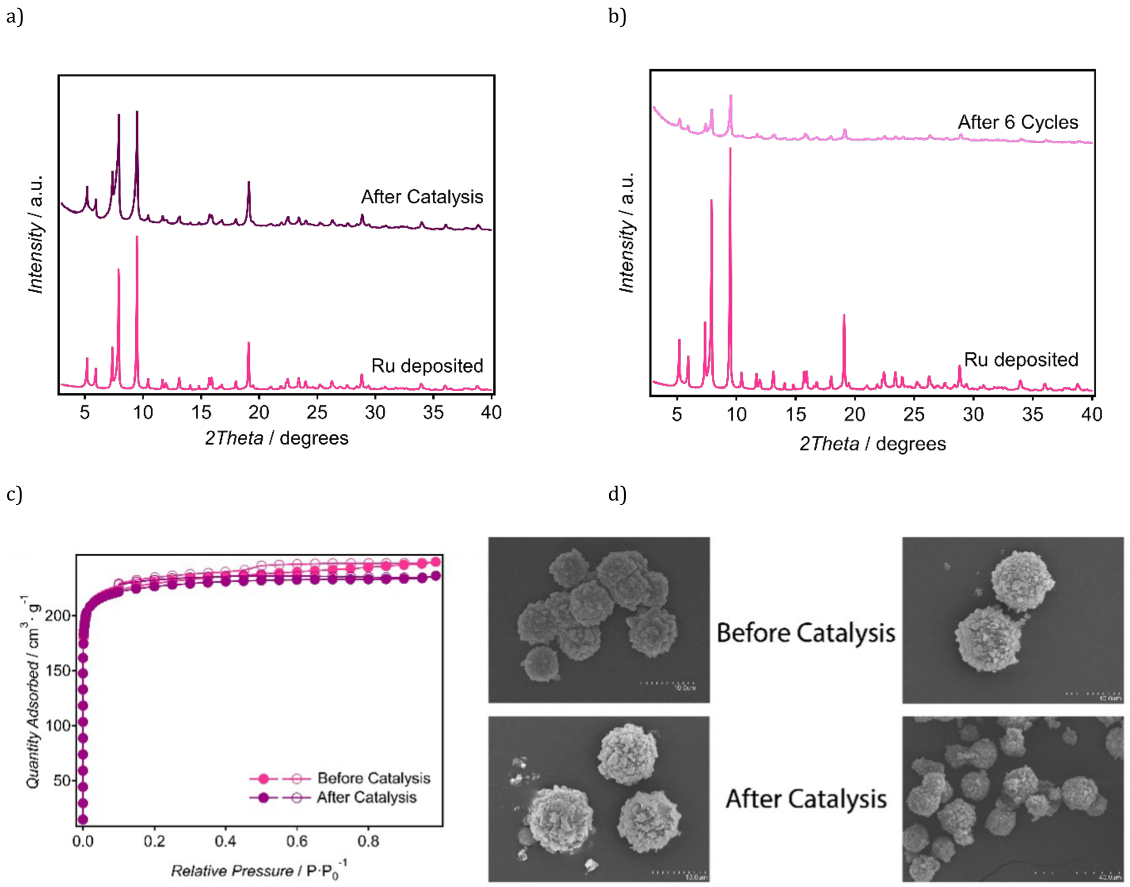

**Figure S33.** Comparison of PXRD of $RuO_x$@MUV-10(Sr), a) before (bottom) and after catalysis (top); b) before photocatalysis (bottom) and after 6 cycles of photocatalysis (top). c) N2 isotherms showing minimum changes before and after reuses. d) SEM imaging of the catalyst confirming retention of the morphology after reuses.

## S.9.5. XPS BEFORE AND AFTER PHOTOCATALYTIC SABATIER REACTION

$RuO_x$@MUV-10(Sr) Before Photocatalytic Sabatier Reaction.

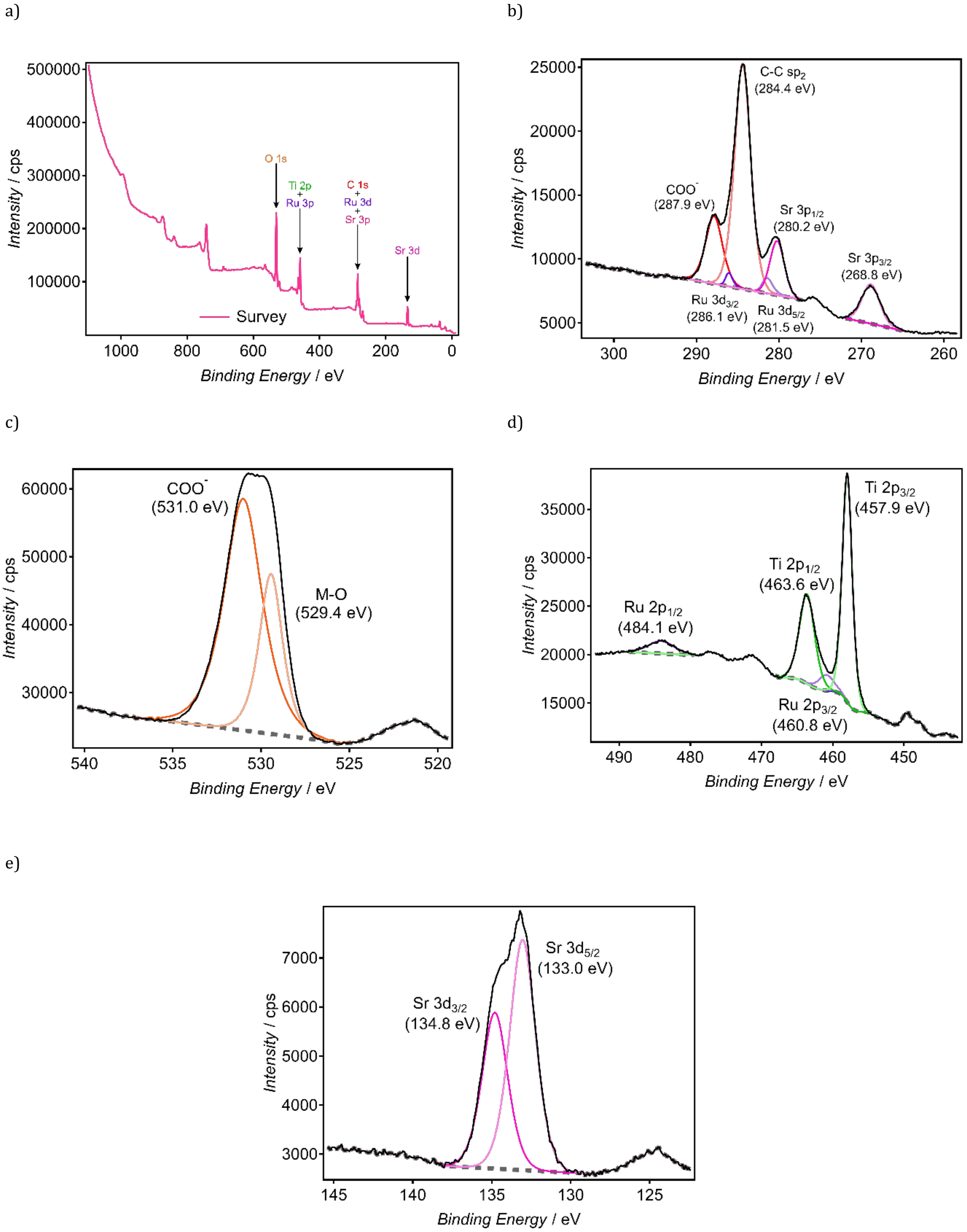


**Figure S34.** XPS a) Survey, b) C 1s + Ru 3d + Sr 3p, c) O 1s, d) Ru 3p + Ti 2p, e) Sr 3d of $RuO_x$@MUV-10(Sr) Before Photocatalytic Sabatier reaction.

$RuO_x$@MUV-10(Sr) After Photocatalytic Sabatier Reaction

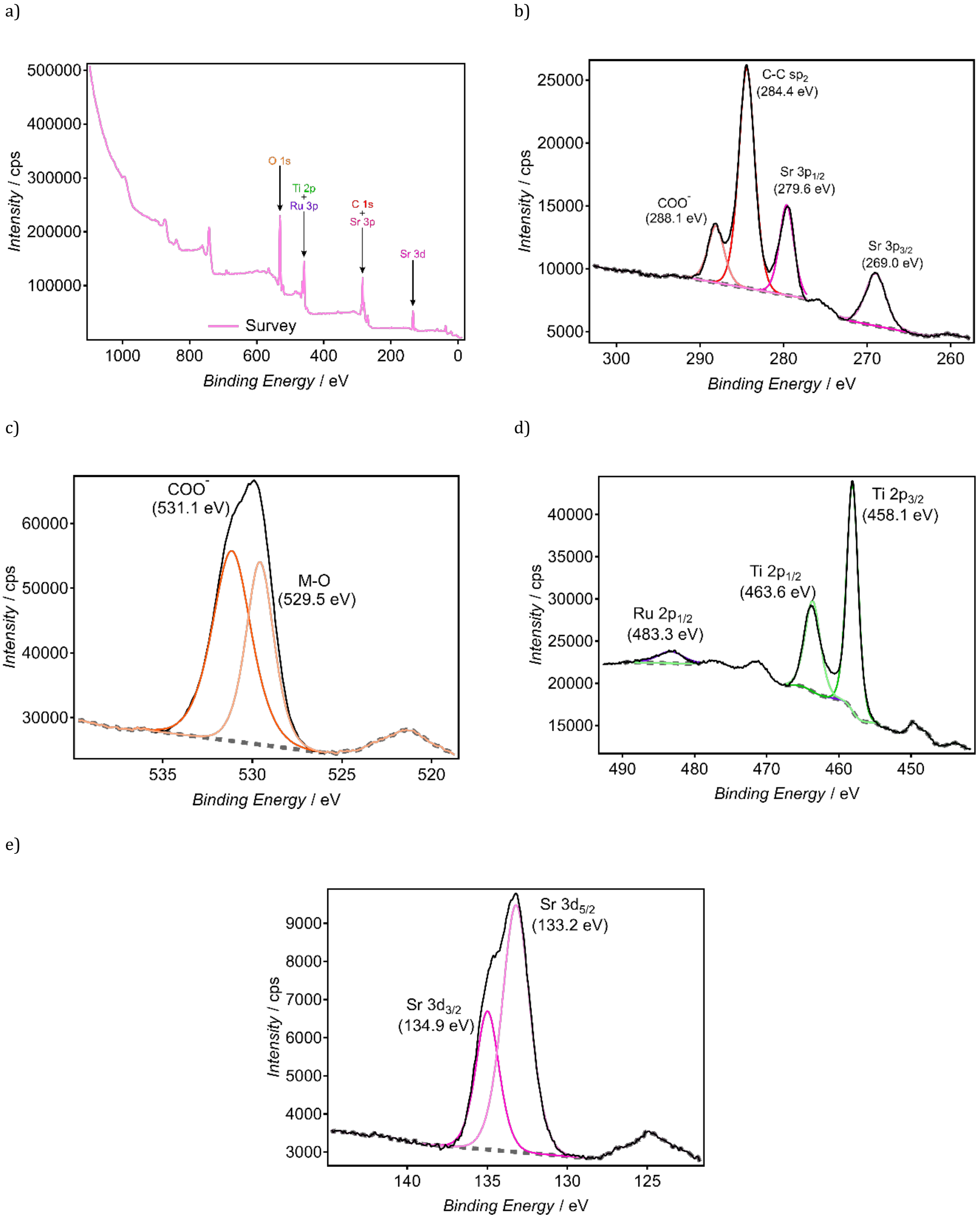


**Figure S35.** XPS a) Survey, b) C 1s + Ru 3d + Sr 3p, c) O 1s, d) Ru 3p + Ti 2p, e) Sr 3d of $RuO_x$@MUV-10(Sr) After Photocatalytic Sabatier reaction.

Comparison of $RuO_x$@MUV-10(Sr) Before and After Photocatalytic Sabatier Reaction

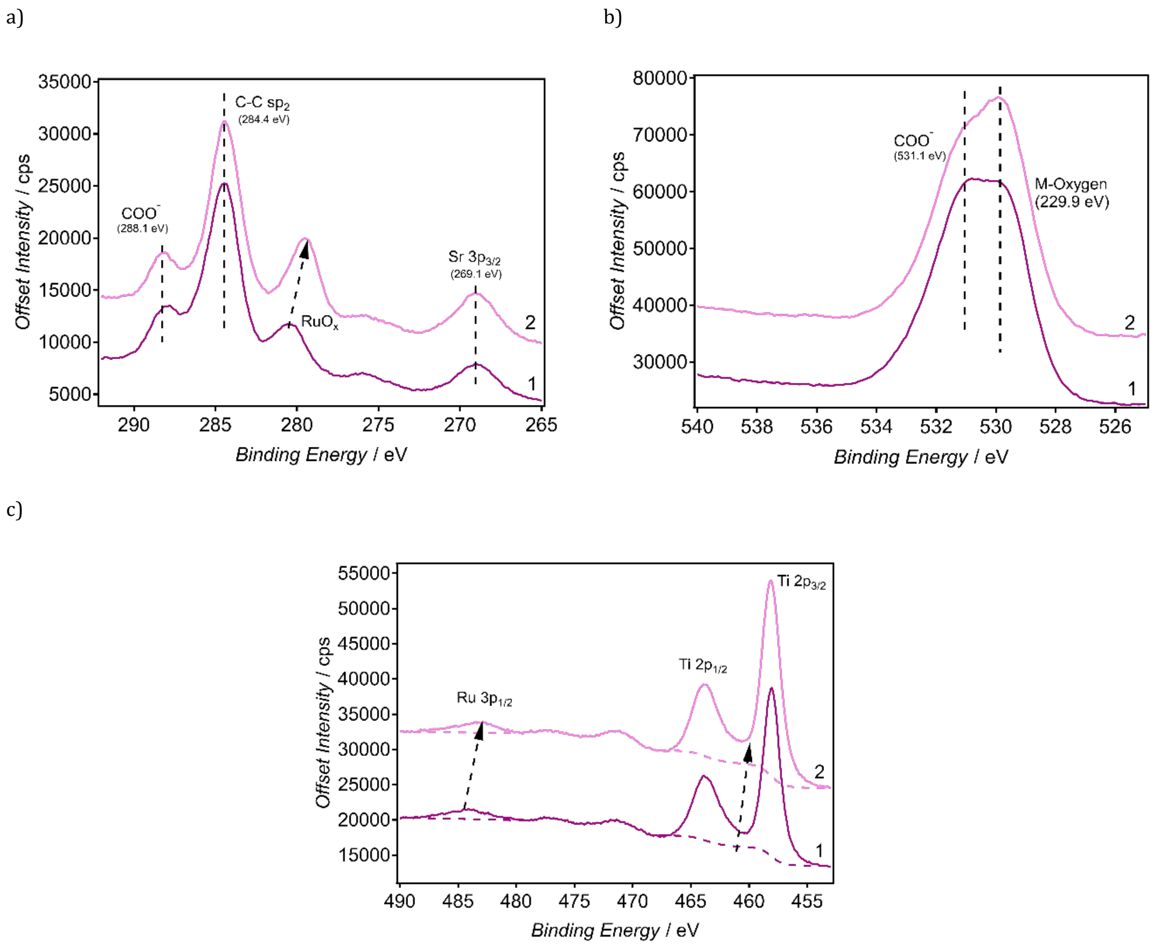


**Figure S36.** Comparison of XPS a) C 1s, b) O 1s, and c) Ti 2p, zones of $RuO_x$@MUV-10Sr) Before (1, dark pink) and After (2, light pink) Photocatalytic Sabatier reaction.

## S.9.6. *IN SITU* XPS BEFORE AND AFTER $H_2$ TREATMENT

$RuO_x$@MUV-10(Sr) Before $H_2$ treatment.

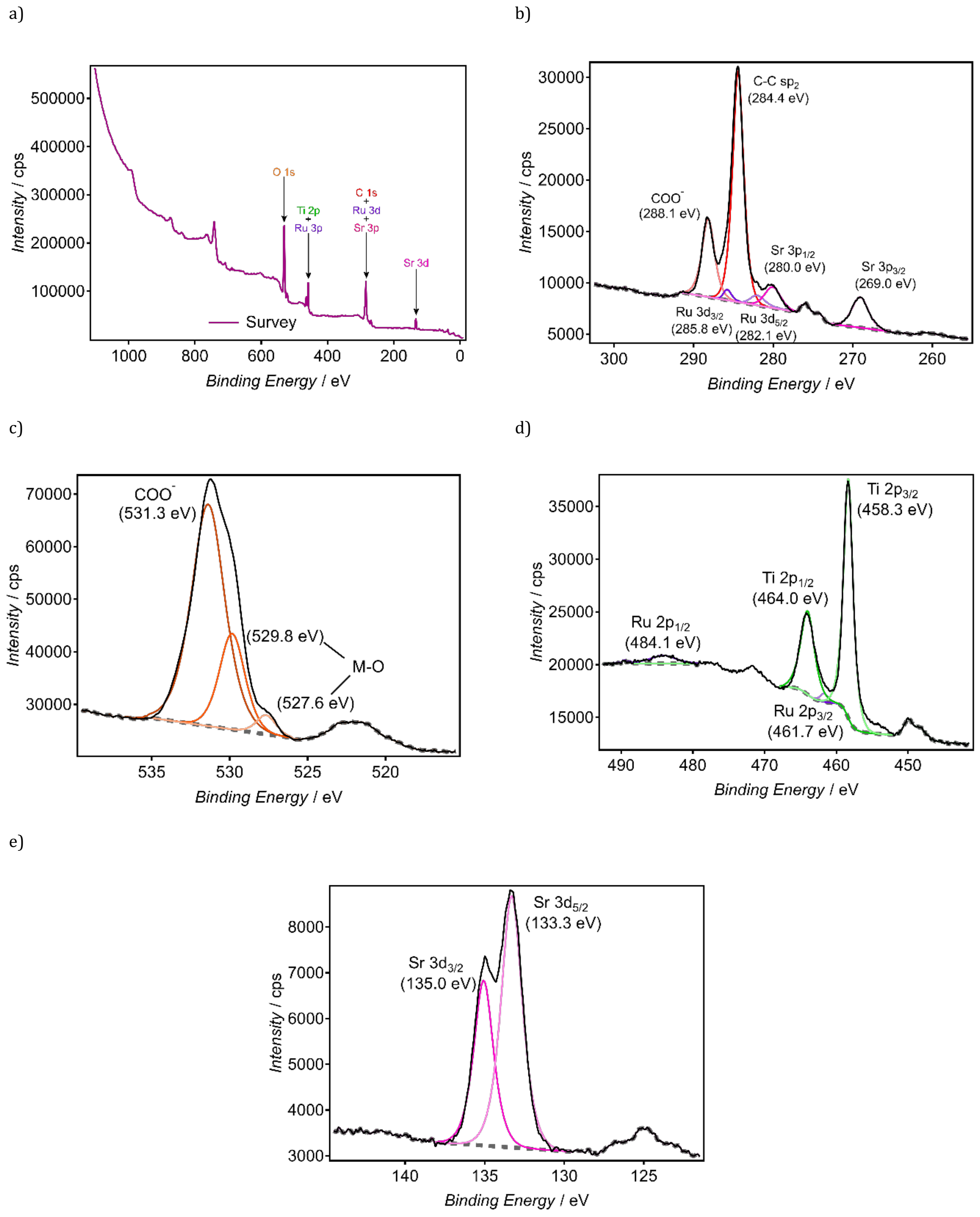


**Figure S37.** XPS a) Survey, b) C 1s + Ru 3d + Sr 3p, c) O 1s, d) Ru 3p + Ti 2p, e) Sr 3d of $RuO_x$@MUV-10(Sr) Before $H_2$ treatment.

$RuO_x$@MUV-10(Sr) After $H_2$ treatment.

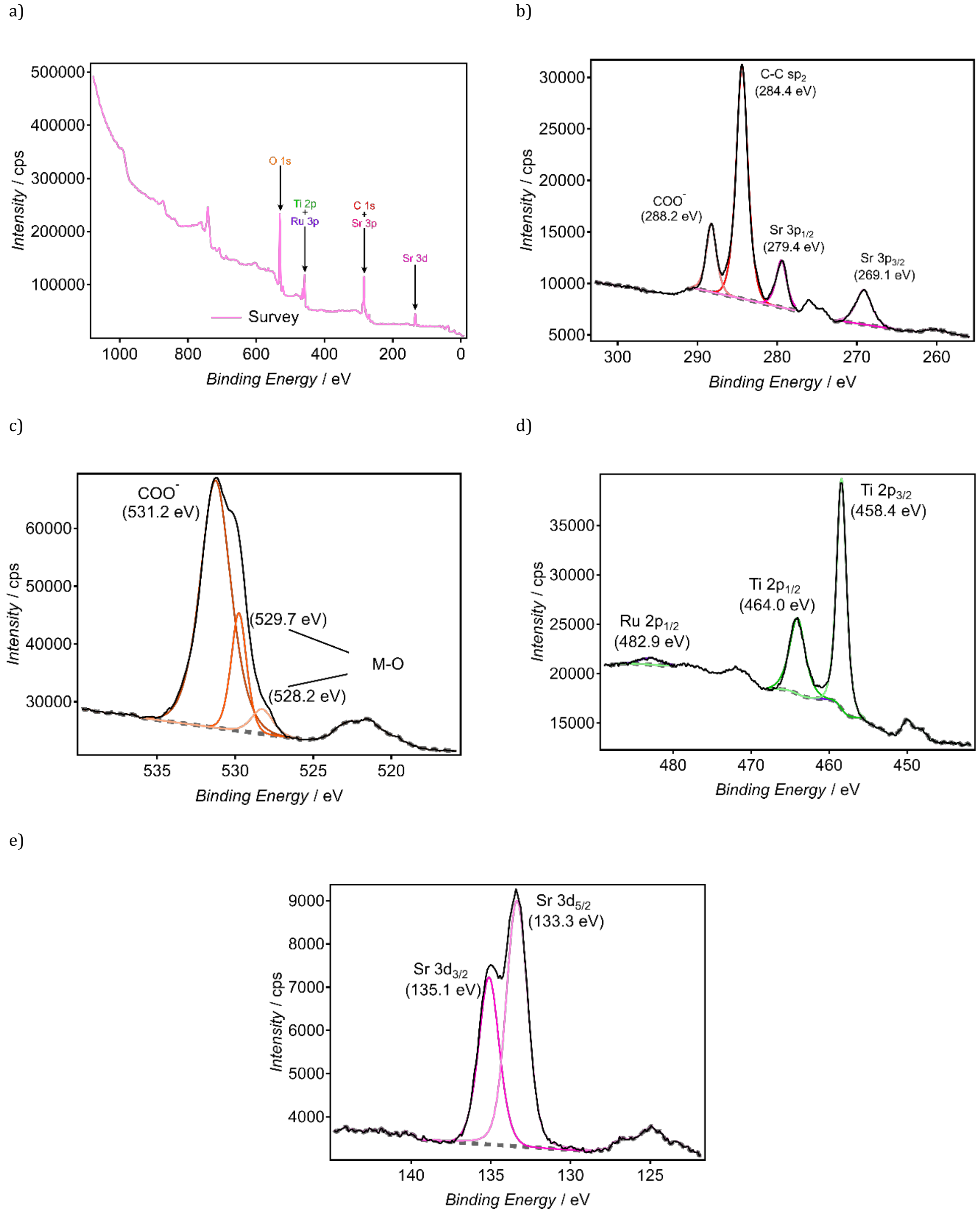


**Figure S38.** XPS a) Survey, b) C 1s + Ru 3d + Sr 3p, c) O 1s, d) Ru 3p + Ti 2p, e) Sr 3d of $RuO_x$@MUV-10(Sr) After $H_2$ treatment.

Comparison of $RuO_x$@MUV-10(Sr) Before and After $H_2$ treatment.

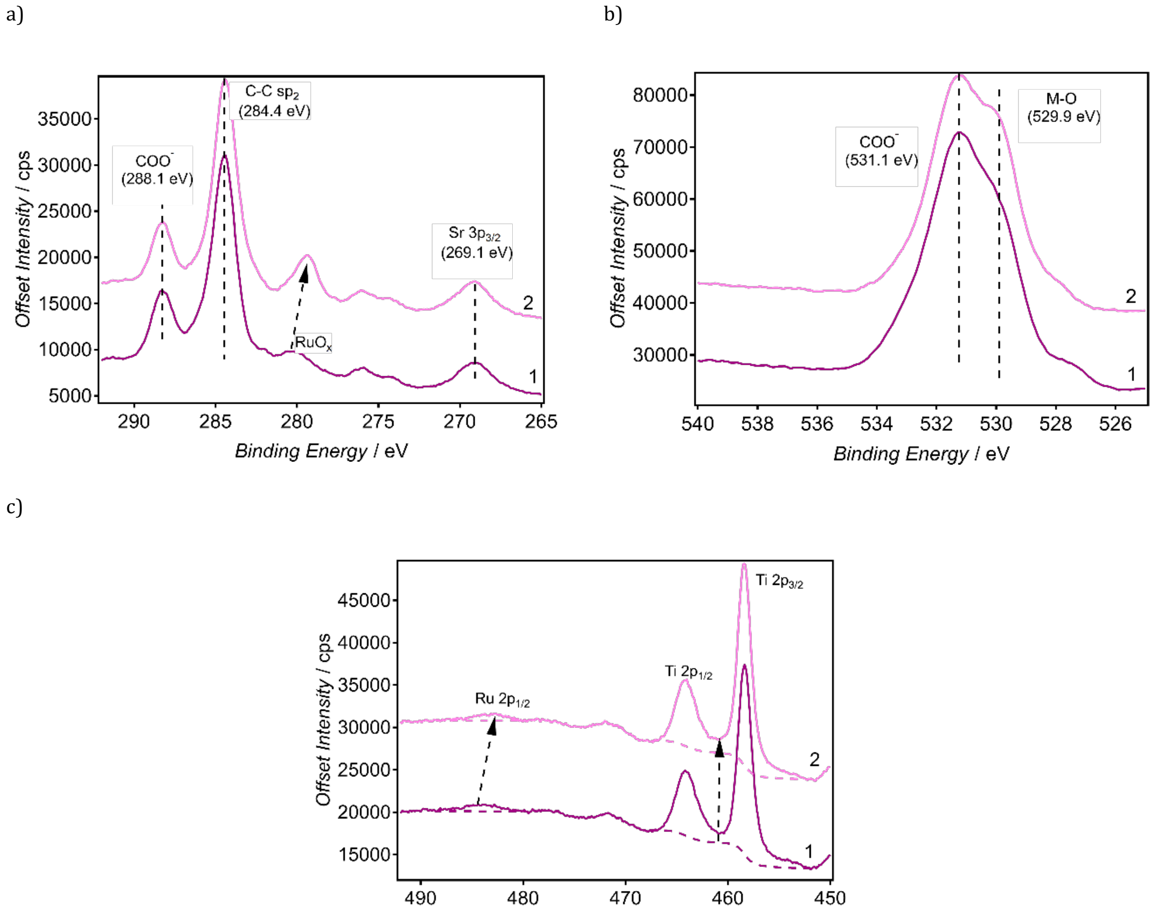


**Figure S39.** Comparison of XPS a) C 1s, b) O 1s, and c) Ti 2p, zones of $RuO_x$@MUV-10(Sr) Before (1, dark pink) and After (2, light pink) $H_2$ treatment.

## S.9.7. TRANSMISION ELECTRONIC MICROSCOPY AFTER REUSES CYCLES.

$RuO_x$@MUV-10(Sr) after reuses cycles.

a)

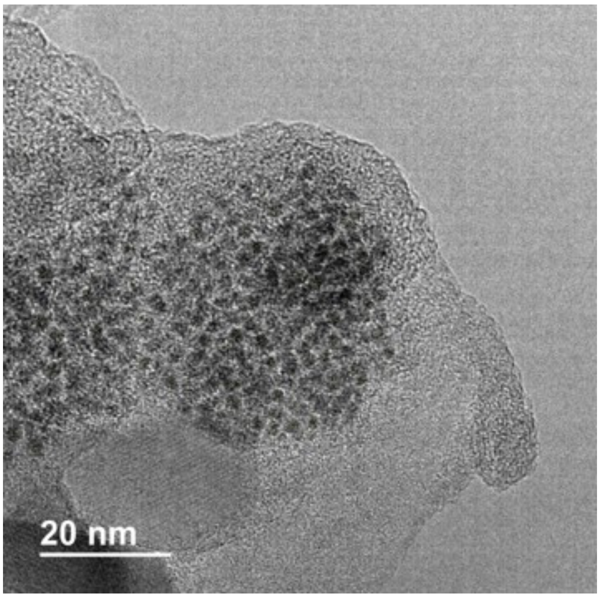


b)

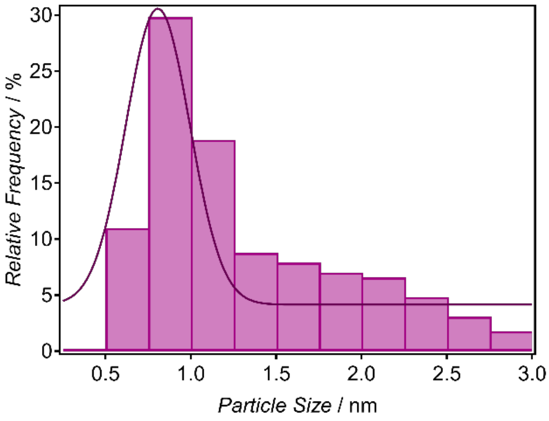


**Figure S40.** a) TEM image of $RuO_x$@MUV-10(Sr) after 6 cycles of Photocatalytic Sabatier reaction, with b) size distribution.

## S.9.8. REPRODUCIBILITY OF RuOx PHOTODEPOSITION AND PHOTOCATALYTIC PERFORMANCE

An additional $RuO_x$@MUV-10(Sr) sample prepared from a second, independent MUV-10(Sr) batch confirms excellent batch-to-batch reproducibility in terms of both microstructure and photocatalytic performance. The new sample displays an average $RuO_x$ particle size of 0.91 ± 0.14 nm, a $CO_2$ conversion of 66.2%, and a $CH_4$ selectivity of 99.4%, closely matching the values obtained with the first batch. This comparison confirms that the photodeposition process is consistent and does not introduce significant variability in structure or catalytic performance.

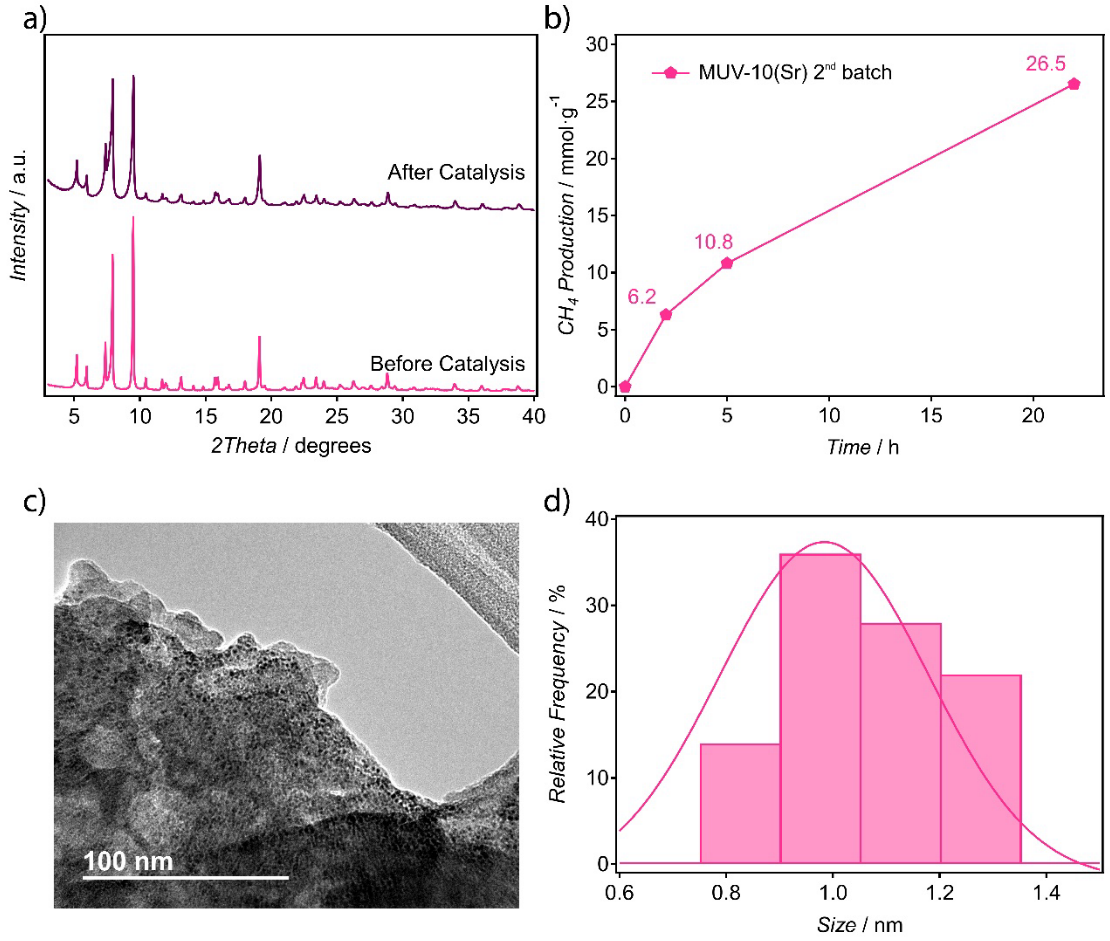


**Figure S41.** Characterization and catalytic performance of a $RuO_x$@MUV-10(Sr) sample prepared from a second, independent MUV-10(Sr) batch. (a) PXRD patterns of $RuO_x$@MUV-10(Sr) before and after catalysis. (b) Time evolution of $CH_4$ production over 20 h. (c) TEM image of $RuO_x$@MUV-10(Sr). (d) $RuO_x$ particle size distribution derived from TEM analysis.

## S.9.9. PHOTOCATALYTIC ACTIVITY OF PRISTINE MUV-10(M) FRAMEWORKS.

To decouple the intrinsic contribution of the MUV-10(M) framework from the role of the RuOx co-catalyst, we conducted photocatalytic tests using the pristine solids under standard reaction conditions. Figure S42 shows the time-dependent $CH_4$ production for MUV-10(Ca), MUV-10(Sr), and MUV-10(Ba). MUV-10(Sr) consistently outperformed the other two analogues, while MUV-10(Ca) and MUV-10(Ba) displayed lower and comparable activities.

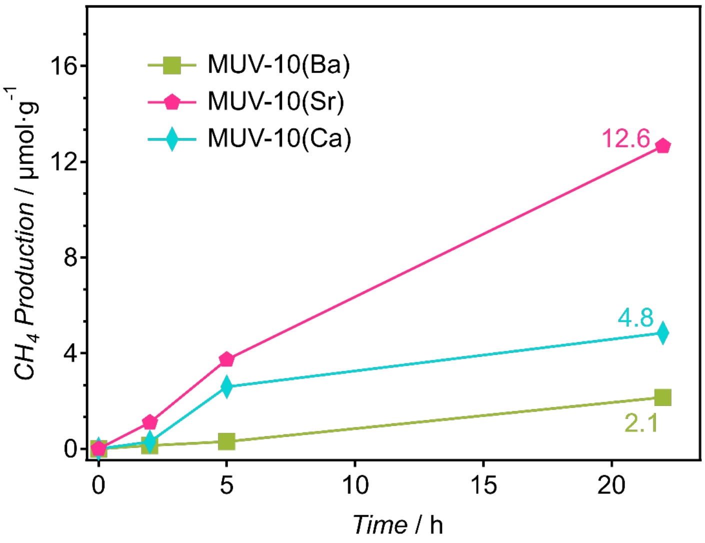


**Figure S42**. Photocatalytic activity of pristine MUV-10(Ca), MUV-10(Sr), and MUV-10(Ba) during the Sabatier reaction. Reaction conditions: catalyst mass = 15 mg; $P_{H2}$ = 0.3 bar, $P_{CO2}$ = 1.2 bar; temperature = 200 ºC; irradiation with 150 W Hg-Xe lamp equipped with AM 1.5G filter.

## S.9.10. STABILITY TESTS IN AIR AND WATER.

To rationalize the inferior photocatalytic performance of MUV-10(Ba), we conducted comparative stability tests for MUV-10(M) solids (M = Ca, Sr, Ba) upon 24 h exposure to air and immersion in Milli-Q water. PXRD, SEM and EDX analyses confirm the structural robustness of MUV-10(Ca) and MUV-10(Sr), while MUV-10(Ba) exhibits significant morphological degradation and partial Ba leaching as shown below.

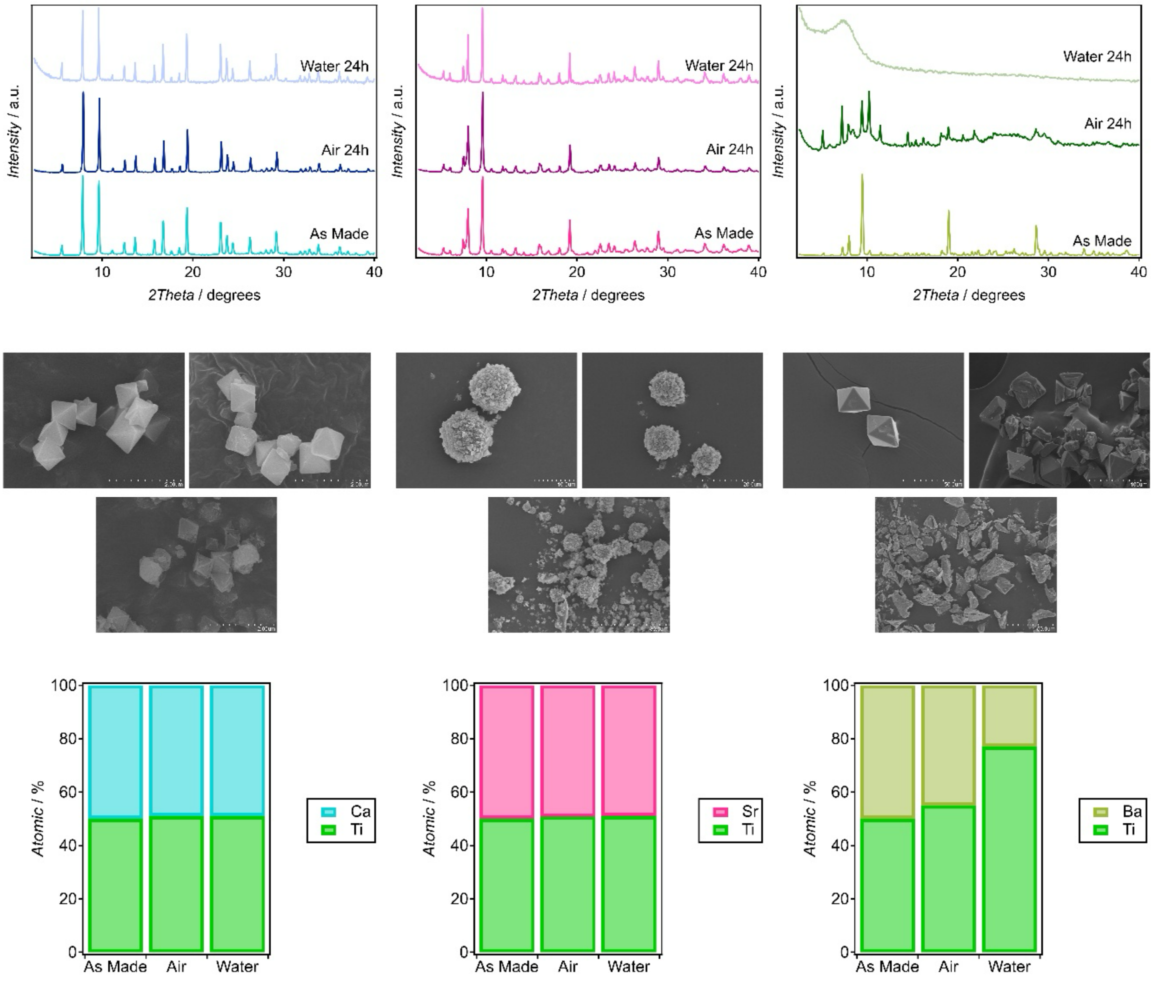


**Figure S43.** Stability comparison of MUV-10(M) frameworks (M = Ca, Sr, Ba) after 24-hour exposure to ambient air and immersion in Milli-Q water. (top) PXRD patterns of the as-made samples and after exposure to air and water, respectively. (middle) SEM images showing morphology evolution under the same conditions. (bottom) EDX-derived atomic compositions (Ti/M ratio) before and after exposure, revealing significant Ba leaching in MUV-10(Ba) upon water immersion, in contrast to the stability of Ca and Sr analogues.

## S.9.11. PARTICLE SIZE EFFECTS ON MUV-10(Sr) PHOTOCATALYTIC PERFORMANCE.

To explore the influence of particle size, we synthesized a nanosized version of MUV-10(Sr) with an average crystallite diameter of 50 ± 10 nm, compared to ~600 nm in the standard sample. RuOx nanoparticles were photodeposited using the same protocol, yielding similar Ru content (1 wt%) and nanoparticle size (0.96 ± 0.18 nm vs. 0.85 ± 0.47 nm).

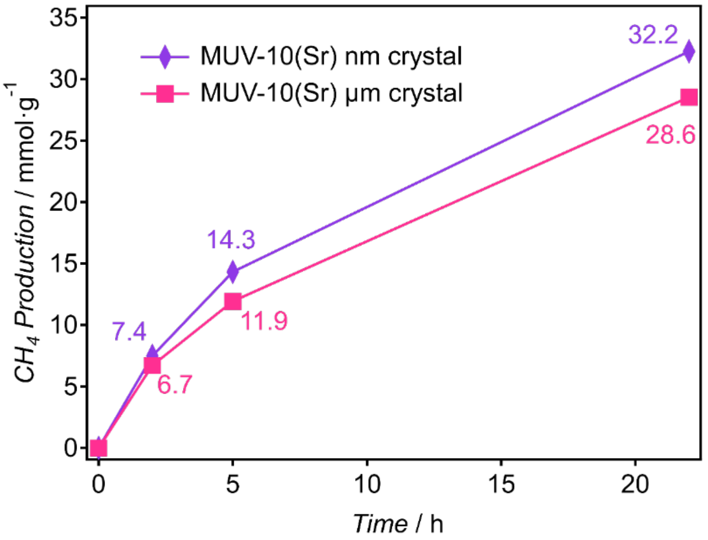


**Figure S44.** Comparison of photocatalytic $CH_4$ production during $CO_2$ hydrogenation over $RuO_x$@MUV-10(Sr) samples prepared with different particle sizes (~600 nm vs. ~50 nm).

## S.10. BENCHMARKING OF PHOTOCATALYTIC $CO_2$ METHANATION PERFORMANCE

To contextualize the activity of MUV-10(Sr) in $CO_2$ methanation, we compare its performance with a range of reported MOF-based photocatalysts employing ruthenium or other co-catalysts under similar reaction conditions.

**Table S7.** Summary of $CH_4$ production rates for MUV-10(M) photocatalysts compared with selected MOF-based systems reported in the literature. All reactions were carried out under comparable gas-phase conditions ($P_{H_2}$ ≈ 1.05 bar, $P_{CO_2}$ ≈ 0.25 bar, 200 °C, 22 h) unless otherwise specified.

| Entry | Photocatalyst | Co-catalyst | Irradiation source | Reaction conditions | Rate. µmol g$^{-1}$ h$^{-1}$ (Product) | Ref. |
|---|---|---|---|---|---|---|
| **1** | MUV-10(Sr) | $RuO_x$ (1 wt%) | Simulated solar light irradiation (150 W Hg-Xe lamp, AM 1.5 filter) | 200 °C, $PH_2$ = 1.05 bar, $PCO_2$ = 0.25 bar, 22h | 1296 ($CH_4$) | This work |
| **2** | MUV-10(Sr) | - | Simulated solar light irradiation (150 W Hg-Xe lamp, AM 1.5 filter) | 200 °C, $PH_2$ = 1.05 bar, $PCO_2$ = 0.25 bar, 22h | 0 ($CH_4$) | This work |
| **3** | MUV-10(Ca) | $RuO_x$ (1 wt%) | Simulated solar light irradiation (150 W Hg-Xe lamp, AM 1.5 filter) | 200 °C, $PH_2$ = 1.05 bar, $PCO_2$ = 0.25 bar, 22h | 540 ($CH_4$) | This work |
| **4** | MUV-10(Ca) | - | Simulated solar light irradiation (150 W Hg-Xe lamp, AM 1.5 filter) | 200 °C, $PH_2$ = 1.05 bar, $PCO_2$ = 0.25 bar, 22h | 0.50 ($CH_4$) | This work |
| **5** | MUV-10(Ba) | $RuO_x$ (1 wt%) | Simulated solar light irradiation (150 W Hg-Xe lamp, AM 1.5 filter) | 200 °C, $PH_2$ = 1.05 bar, $PCO_2$ = 0.25 bar, 200 °C, 22h | 690 ($CH_4$) | This work |
| **6** | MUV-10(Ba) | - | Simulated solar light irradiation (150 W Hg-Xe lamp, AM 1.5 filter) | 200 °C, $PH_2$ = 1.05 bar, $PCO_2$ = 0.25 bar, 22h | 0.66 ($CH_4$) | This work |
| **7** | UiO-66(Zr/Ti)-$NO_2$ | $RuO_x$ (1 wt%) | Simulated solar light irradiation (150 W Hg-Xe lamp, AM 1.5 filter) | 200 °C, $PH_2$ = 1.05 bar, $PCO_2$ = 0.25 bar, 22h | 230 ($CH_4$) | 5 |
| **8** | UiO-66 (Zr/Ce/Ti) | $RuO_x$ (1 wt%) | Simulated solar light irradiation (150 W Hg-Xe lamp, AM 1.5 filter) | 200 °C, $PH_2$ = 1.05 bar, $PCO_2$ = 0.25 bar, 22h | 82 ($CH_4$) | 16 |
| **9** | UiO-66 (Zr/Ce/Ti) | $RuO_x$ (1 wt%) | UV-Vis light irradiation (150 W Hg-Xe lamp) | 200 °C, $PH_2$ = 1.05 bar, $PCO_2$ = 0.25 bar, 22h | 130 ($CH_4$) | 16 |
| **10** | MIP-208(Ti) | $RuO_x$ (0.76 wt%) | Simulated solar light irradiation (150 W Hg-Xe lamp, AM 1.5 filter) | 200 °C, $PH_2$ = 1.05 bar, $PCO_2$ = 0.25 bar, 22h | 36 ($CH_4$) | 17 |
| **11** | MIL-125 (Ti)-$NH_2$ | $RuO_x$ (1 wt%) | Simulated solar light irradiation (150 W Hg-Xe lamp, AM 1.5 filter) | 200 °C, $PH_2$ = 1.05 bar, $PCO_2$ = 0.25 bar, 22h | 42 ($CH_4$) | 18 |
| **12*** | UiO-66-$NH_2$ | $TiO_2$ + Au (10wt%) | Simulated solar light irradiation (150 W-Xe lamp, 300-800nm) | 30ºC, $CO_2$:$H_2O$ = 24:1, flow = 0.3mL·min$^{-1}$, 5h | 36 ($H_2$) 26 ($CH_4$) | 19 |
| **13** | UiO-66 | $TiO_2$ (80wt%) | Simulated solar light irradiation (300 W-Xe lamp) | 0ºC, $CO_2$ saturated in $H_2O$, 1 bar, 1h | 18 ($CH_4$) 2 (CO) | 20 |
| **14** | UiO-66 | $Pd_3Cu$ | Simulated solar light irradiation (300 W-Xe lamp, 200mW·cm$^{-2}$) | 200 ºC, $PH_2$ = 9.4 bar, $PCO_2$ = 3.1 bar, 12h | 340 (MeOH) | 21 |
| **15** | Fe-POMOF | - | Simulated solar light irradiation (300 W-Xe lamp) | 200 ºC, $PCO_2$ = 1 bar, 28mL $H_2O$ + 2mL TEOA | 154 ($CH_4$) 4 (CO) | 22 |
| **16** | MOF-808-EDTA | $[Ru(bby)_3]Cl_2$ | Simulated solar light irradiation (300 W-Xe lamp, 420-760 nm) | 25 ºC, $PCO_2$ = 1 bar, 30 mL MeCN + 10mL $H_2O$ + 10mL TEOA | 23.4 ($CH_4$) 3.4 (CO) 167.1 (HCOOH) | 23 |
| **17** | MOF-808-CuNi | $[Ru(bby)_3]Cl_2$ | Simulated solar light irradiation (300 W-Xe lamp, 420-760 nm) | 25 ºC, $PCO_2$ = 1 bar, 30 mL MeCN + 10mL $H_2O$ + 10mL TEOA | 158.7 ($CH_4$) 2.3 (CO) 0.2 (HCOOH) | 23 |

## S.11. REFERENCES


(1) Hong, K.; Bak, W.; Chun, H. Robust Molecular Crystals of Titanium(IV)-Oxo-Carboxylate Clusters Showing Water Stability and $CO_2$ Sorption Capability. *Inorg. Chem.* **2014**, *53* (14), 7288–7293. https://doi.org/10.1021/ic500629y.

(2) Coelho, A. A. *TOPAS* and *TOPAS-Academic* : An Optimization Program Integrating Computer Algebra and Crystallographic Objects Written in C++. *J Appl Crystallogr* **2018**, *51* (1), 210–218. https://doi.org/10.1107/s1600576718000183.

(3) Howarth, A. J.; Peters, A. W.; Vermeulen, N. A.; Wang, T. C.; Hupp, J. T.; Farha, O. K. Best Practices for the Synthesis, Activation, and Characterization of Metal–Organic Frameworks. *Chem. Mater.* **2017**, *29* (1), 26–39. https://doi.org/10.1021/acs.chemmater.6b02626.

(4) Castells-Gil, J.; Padial, N. M.; Almora-Barrios, N.; Albero, J.; Ruiz-Salvador, A. R.; González-Platas, J.; García, H.; Martí-Gastaldo, C. Chemical Engineering of Photoactivity in Heterometallic Titanium–Organic Frameworks by Metal Doping. *Angew. Chem. Int. Ed.* **2018**, *57* (28), 8453–8457. https://doi.org/10.1002/anie.201802089.

(5) Rueda-Navarro, C. M.; Khalil, Z. A.; Melillo, A.; Ferrer, B.; Montero, R.; Longarte, A.; Daturi, M.; Vayá, I.; El-Roz, M.; Martínez-Martínez, V.; Baldoví, H. G.; Navalón, S. Solar Gas-Phase $CO_2$ Hydrogenation by Multifunctional UiO-66 Photocatalysts. *ACS Catal.* **2024**, *14* (9), 6470–6487. https://doi.org/10.1021/acscatal.4c00266.

(6) Mondal, P.; Brahma, B. K.; Vali, D. K.; Ray, J.; Kasu, J. V. N.; Gangopadhyay, A.; Laha, S.; Adhikari, U. Calcium-Based Metal-Organic Framework: Detection and Idiosyncratic Removal of Copper by Nano-Particle Deposition. *Chem. A Eur. J.* **2024**, *30* (35), e202400587. https://doi.org/10.1002/chem.202400587.

(7) Ibrahim, A. A.; Ali, S. L.; Adly, M. S.; El-Hakam, S. A.; Samra, S. E.; Ahmed, A. I. Green Construction of Eco-Friendly Phosphotungstic Acid Sr-MOF Catalysts for Crystal Violet Removal and Synthesis of Coumarin and Xanthene Compounds. *RSC Adv.* **2021**, *11* (59), 37276–37289. https://doi.org/10.1039/d1ra07160b.

(8) Sompalli, N. K.; Li, Y.; Li, J.; Kuppusamy, S. An Innovative Triple Interface Reinforced Photocatalytic System Based on BiOCl/BaTiO3@Co-BDC-MOF Composite for the Simultaneous Detoxification of Cr(VI) and Sulfamethoxazole. *Environ. Res.* **2024**, *259*, 119532. https://doi.org/10.1016/j.envres.2024.119532.

(9) Clausius, R. Ueber Die Bewegende Kraft Der Wärme Und Die Gesetze, Welche Sich Daraus Für Die Wärmelehre Selbst Ableiten Lassen. *Ann. Phys.* **1850**, *155* (3), 368–397. https://doi.org/10.1002/andp.18501550306.

(10) Shannon, R. D. Revised Effective Ionic Radii and Systematic Studies of Interatomic Distances in Halides and Chalcogenides. *Acta Crystallogr. Sect. A: Cryst. Phys., Diffr., Theor. Gen. Crystallogr.* **1976**, *32* (5), 751–767. https://doi.org/10.1107/s0567739476001551.

(11) Fabrizio, K.; Lazarou, K. A.; Payne, L. I.; Twight, L. P.; Golledge, S.; Hendon, C. H.; Brozek, C. K. Tunable Band Gaps in MUV-10(M): A Family of Photoredox-Active MOFs with Earth-Abundant Open Metal Sites. *J. Am. Chem. Soc.* **2021**, *143* (32), 12609–12621. https://doi.org/10.1021/jacs.1c04808.

(12) Heyd, J.; Scuseria, G. E.; Ernzerhof, M. Hybrid Functionals Based on a Screened Coulomb Potential. *J. Chem. Phys.* **2003**, *118* (18), 8207–8215. https://doi.org/10.1063/1.1564060.

(13) Kresse, G.; Furthmüller, J. Efficiency of Ab-Initio Total Energy Calculations for Metals and Semiconductors Using a Plane-Wave Basis Set. *Comput. Mater. Sci.* **1996**, *6* (1), 15–50. https://doi.org/10.1016/0927-0256(96)00008-0.

(14) Kresse, G.; Furthmüller, J. Efficient Iterative Schemes for *Ab Initio* Total-Energy Calculations Using a Plane-Wave Basis Set. *Phys. Rev. B* **1996**, *54* (16), 11169–11186. https://doi.org/10.1103/physrevb.54.11169.

(15) Momma, K.; Izumi, F. VESTA 3 for Three-Dimensional Visualization of Crystal, Volumetric and Morphology Data. *J. Appl. Crystallogr.* **2011**, *44* (6), 1272–1276. https://doi.org/10.1107/s0021889811038970.

(16) Cabrero-Antonino, M.; Melillo, A.; Montero-Lanzuela, E.; Álvaro, M.; Ferrer, B.; Vayá, I.; Baldoví, H. G.; Navalón, S. Solar-Driven Gas Phase Photocatalytic CO2 Methanation by Multimetallic UiO-66 Solids Decorated with RuOx Nanoparticles. *Chem. Eng. J.* **2023**, *468*, 143553. https://doi.org/10.1016/j.cej.2023.143553.

(17) Wang, S.; Cabrero-Antonino, M.; Navalón, S.; Cao, C.; Tissot, A.; Dovgaliuk, I.; Marrot, J.; Martineau-Corcos, C.; Yu, L.; Wang, H.; Shepard, W.; García, H.; Serre, C. A Robust Titanium Isophthalate Metal-Organic Framework for Visible-Light Photocatalytic CO2 Methanation. *Chem* **2020**, *6* (12), 3409–3427. https://doi.org/10.1016/j.chempr.2020.10.017.

(18) Cabrero-Antonino, M.; Ferrer, B.; Baldoví, H. G.; Navalón, S. Toward Solar-Driven Photocatalytic CO2 Methanation under Continuous Flow Operation Using Benchmark MIL-125(Ti)–NH2 Supported Ruthenium Nanoparticles. *Chem. Eng. J.* **2022**, *445*, 136426. https://doi.org/10.1016/j.cej.2022.136426.

(19) Duflot, M.; Marchal, C.; Caps, V.; Artero, V.; Christoforidis, K.; Keller, V. Optimization of NH2-UiO-66/TiO2/Au Composites for Enhanced Gas-Phase CO2 Photocatalytic Reduction into CH4. *Catal. Today* **2023**, *413*, 114018. https://doi.org/10.1016/j.cattod.2023.01.025.

(20) Ma, Y.; Tang, Q.; Sun, W.-Y.; Yao, Z.-Y.; Zhu, W.; Li, T.; Wang, J. Assembling Ultrafine TiO2 Nanoparticles on UiO-66 Octahedrons to Promote Selective Photocatalytic Conversion of CO2 to CH4 at a Low Concentration. *Appl. Catal. B: Environ.* **2020**, *270*, 118856. https://doi.org/10.1016/j.apcatb.2020.118856.

(21) Ling, L.; Yang, W.; Yan, P.; Wang, M.; Jiang, H. Light-Assisted CO2 Hydrogenation over Pd3Cu@UiO-66 Promoted by Active Sites in Close Proximity. *Angew. Chem. Int. Ed.* **2022**, *61* (12), e202116396. https://doi.org/10.1002/anie.202116396.

(22) Huang, Q.; Niu, Q.; Li, X.-F.; Liu, J.; Sun, S.-N.; Dong, L.-Z.; Li, S.-L.; Cai, Y.-P.; Lan, Y.-Q. Demystifying the Roles of Single Metal Site and Cluster in CO2 Reduction via Light and Electric Dual-Responsive Polyoxometalate-Based Metal-Organic Frameworks. *Sci. Adv.* **2022**, *8* (49), eadd5598. https://doi.org/10.1126/sciadv.add5598.

(23) Li, J.; Huang, H.; Xue, W.; Sun, K.; Song, X.; Wu, C.; Nie, L.; Li, Y.; Liu, C.; Pan, Y.; Jiang, H.-L.; Mei, D.; Zhong, C. Self-Adaptive Dual-Metal-Site Pairs in Metal-Organic Frameworks for Selective CO2 Photoreduction to CH4. *Nat. Catal.* **2021**, *4* (8), 719–729. https://doi.org/10.1038/s41929-021-00665-3.
